# Recurrent intra-tumour heterogeneity is a hallmark of metastatic prostate cancer

**DOI:** 10.1038/s41467-026-74334-z

**Published:** 2026-06-19

**Authors:** Sirui Weng, Lachlan Cain, James Comben, Yangyi Zhang, Timothy Semple, Sara Alaei, David Yoannidis, Luciano Martelotto, Catherine Mitchell, Anupama Pasam, Richard J. Young, Benjamin Blyth, Joy Hendley, Yuzhou Feng, Heather Thorne, Roslyn Wallace, Joanna Chan, Julia Como, Lisa Devereux, Himisha Beltran, Daniel Wetterskog, Scott Williams, Anthony T. Papenfuss, Belinda Parker, Paul Neeson, Gerhardt Attard, David Quigley, David L. Goode, Richard B. Pearson, Luc Furic, Anna S. Trigos, Shahneen Sandhu

**Affiliations:** 1 Peter MacCallum Cancer Centre, Melbourne, VIC Australia; 2 Sir Peter MacCallum Department of Oncology, The University of Melbourne, Parkville, VIC Australia; 3 St. Vincent’s Institute of Medical Research, Fitzroy, VIC Australia; 4 Australian Regenerative Medicine Institute, Monash University, Melbourne, VIC Australia; 5 Adelaide Centre for Epigenetics, The University of Adelaide, Adelaide, SA Australia; 6 South Australian immunoGENomics Cancer Institute, The University of Adelaide, Adelaide, SA Australia; 7 The University of Adelaide, Adelaide, SA Australia; 8 Department of Clinical Pathology, Faculty of Medicine, Dentistry and Health Sciences, The University of Melbourne, Melbourne, VIC Australia; 9 Department of Medical Oncology, Dana-Farber Cancer Institute, Harvard Medical School, Boston, MA USA; 10 University College London Cancer Institute, London, UK; 11 The Walter and Eliza Hall Institute of Medical Research, Parkville, VIC Australia; 12 Department of Medical Biology, University of Melbourne, Parkville, VIC Australia; 13 Department of Urology, University of California San Francisco, San Francisco, CA USA; 14 Department of Epidemiology & Biostatistics, University of California San Francisco, San Francisco, CA USA; 15 Helen Diller Family Comprehensive Cancer Center, University of California San Francisco, San Francisco, CA USA; 16 Department of Biochemistry and Molecular Biology, Monash University, Clayton, VIC Australia; 17 Department of Biochemistry and Molecular Biology, The University of Melbourne, Parkville, VIC Australia

**Keywords:** Metastasis, Tumour heterogeneity, Cancer genomics, Prostate cancer

## Abstract

The evolution to metastatic disease is a major determinant of cancer mortality. Cancer evolution involves a complex interplay between intrinsic genetics and transcriptional alterations and the microenvironment. To define mechanisms underpinning metastatic heterogeneity in late-stage disease, we focus on metastatic castration-resistant prostate cancer and employed single-cell multi-omics and whole-genome sequencing to deeply profile 34 metastatic lesions obtained from 9 patients through rapid autopsy. We find evolutionary convergence of intra-tumour heterogeneity, characterised by recurrent tumour populations acting as critical functional components of the tumour ecosystem, irrespective of clonal and microenvironmental backgrounds. We find little evidence of the microenvironment driving transcriptional heterogeneity, but there are signatures of co-adaptation between the microenvironment and tumour cells. In contrast, clonal evolution primarily foster widespread transcriptional changes that did not result in de novo functional states. Intra-patient functional convergence of tumour ecosystems across metastases indicates system-level selection pressures that drive the heterogeneity landscape of metastatic castration-resistant prostate cancer. Our findings reveal functional evolutionary convergence of metastatic disease into distinct intra-tumour subpopulations, identifying critical determinants for therapeutic targeting.

## Abstract (web summary)

The evolutionary elements contributing to end-stage prostate cancer are still unclear. Here, single-cell multi-omics and whole-genome sequencing of 34 metastatic lesions obtained from 9 patients by rapid autopsy reveal recurring tumour populations and similar tumour ecosystems across sites, with minimal microenvironmental impact.

## Introduction

Cancer is driven by an evolutionary process involving clonal selection, adaptation and expansion by both genetic and non-genetic factors^1–3^. Epigenetic factors, the surrounding microenvironment and endogenous tumour plasticity are recognised as significant contributors alongside clonal evolution^4–7^. This intricate interplay poses challenges in defining tumour subtypes, predicting tumour evolution, identifying biomarkers of response, and understanding treatment resistance. Currently, our understanding of each of these factors is derived from studies profiling genomic, epigenomic, and microenvironmental landscapes in tumours separately^6,8–10^, with limited research investigating their cooperation to shape cancer evolution. For example, while metastatic disease is linked with complex clonal evolutionary histories, tumour non-genetic factors and local microenvironments are thought to play a role in the development of metastatic heterogeneity, but the exact contribution of these factors remains unknown. Understanding how a diversity of tumour cell types derived from clonal and epigenetic evolution and plasticity, and cells of the microenvironment interrelate with each other is critical to further our understanding of cancer ecosystems and their evolution. In prostate cancer (PC), intrinsic genetic and epigenetic factors together with extrinsic microenvironment and treatment pressures lead to disease heterogeneity^11–16^. In patients with metastatic castration-resistant prostate cancer (mCRPC), cancers have progressed despite androgen deprivation therapy (ADT) and, in many cases, androgen receptor pathway inhibitors (ARPIs), leading to poor clinical outcomes^17–19^. Resistance is often enabled by genetic alterations of the androgen receptor (*AR*) gene and its regulatory elements, with resistant clones seeding metastases, leading to limited clonal metastatic heterogeneity^10,13,20^. Lineage plasticity also plays a pivotal role in resistance, where transition from an adenocarcinoma (AD) phenotype with *AR* expression to a neuroendocrine (NE) phenotype lacking *AR* expression^21^ occurring in around 20% of CRPC patients^22,23^. Furthermore, the mCRPC tumour microenvironment has been shown to play a pivotal role in tumour plasticity^24^. Elucidating the synergistic interactions among key drivers of heterogeneity that underlie resistance in metastatic disease is essential for informing the development of treatment strategies.

In this work, we aim to investigate how clonal evolution, plasticity, and the microenvironment work synergistically to shape the evolution and heterogeneity of mCRPC tumour ecosystems, and their role in treatment resistance. For this, we profile multiple metastatic lesions from individual patients collected via rapid autopsy using matched single-nucleus multiomics and whole-genome DNA sequencing data. This enables an in-depth analysis of intra-lesion tumour heterogeneity within and between patients, and its association with the genetic background of individual cells and the composition of the surrounding microenvironment. We find heterogeneity in metastatic disease was primarily characterised by the recurrent emergence of cell populations exhibiting specific transcriptional profiles independent of genetic background and the surrounding microenvironment. Surprisingly, there is striking consistency across metastatic lesions. Our findings shed light on how transcriptional changes underlie this recurrent intra-tumour plasticity in end stage prostate cancer. This enables defining heterogeneity-driven mechanisms of resistance to standard of care therapies in mCRPC and highlights potential strategies for treating resistant disease.

## Results

### Cohort overview

**Fig. 1:**
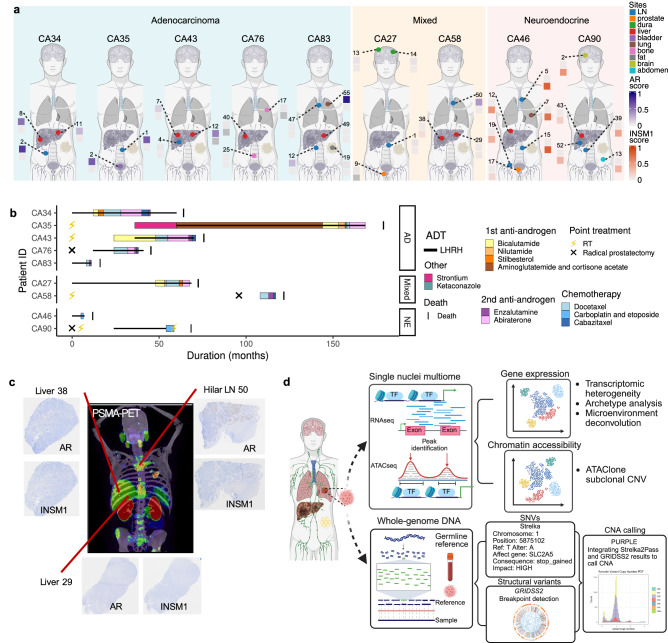
Study overview. **a** Cohort Overview. Metastatic lesions were categorised as adenocarcinoma (AD) or neuroendocrine (NE) according to AR and INSM1 staining, respectively, with most patients presenting exclusively with either AD or NE lesions. Patients CA27 and CA58 were categorised as mixed. CA27 exhibited low levels of AR and INSM1 across lesions; however, no evidence of small-cell morphology was observed. In patient CA58, two liver lesions exhibited elevated INSM1 levels and lacked AR expression, while the hilar lymph node demonstrated greater intensity and a higher proportion of AR staining, with no INSM1 staining present. Created in BioRender. Weng, S. (2026) https://BioRender.com/f6gruh9. **b** Treatment history of patients (*n* = 9). All patients received abiraterone or enzalutamide, except those with neuroendocrine disease, who received platinum-based chemotherapies. **c** Metastatic intra-patient heterogeneity in patient CA58 observed by histopathology and PET imaging. Liver samples showed high expression INSMA1 but not AR, while the hilar lymph node sample had an AD pathology, with high AR and no INSM1. **d** Overview of experimental workflow. 34 samples across 9 patients were selected based on quality and to represent various organs. Samples from CA27, CA34, CA35, CA43, CA46, CA58, and CA76 were processed for 10X Chromium multiome (ATAC+gene expression), CA83 and CA90 for 5’ Single Nuclei RNA Sequencing. Whole-genome sequencing data was at 60X coverage with matching germline reference at 30X. Created in BioRender. Weng, S. (2026) https://BioRender.com/f6gruh9.

Metastatic tissue was collected through the rapid-autopsy CASCADE (CAncer tiSsue Collection After DEath) program at the Peter MacCallum Cancer Centre in Melbourne, Australia^23^ between 2013 and 2019 from nine donors who died from mCRPC. All nine patients were diagnosed with adenocarcinoma (AD) at primary stage, two of which subsequently developed NE disease (Fig. 1a). Patients received ARPI such as abiraterone or enzalutamide, except patients with NE disease, who received platinum-based chemotherapies (Fig. 1b). Histopathology review of the lesions collected at autopsy corroborated the clinical information (Supplementary Data 1, Supplementary Fig. 1). Additionally, we identified that two AD patients displayed inter-lesional heterogeneity, with a combination of AD and NE pathology (Fig. 1a, c). We selected 2–6 samples per patient for a total of 34 samples for deep single-nuclei RNAseq or single-nuclei multiome analyses (Supplementary Data 1) for a total of 177,907 cells across all samples after stringent quality control, with an average of 5391 high-quality cells per sample (Fig. 1d). Matched whole-genome sequencing (WGS) data was generated for all tumour samples and germline references (Fig. 1d). Overall, our cohort represents the main pathological subtypes of mCRPC and across multiple organs from individual patients.

### Clonal evolution leads to transcriptional noise but not de novo functional states

**Fig. 2:**
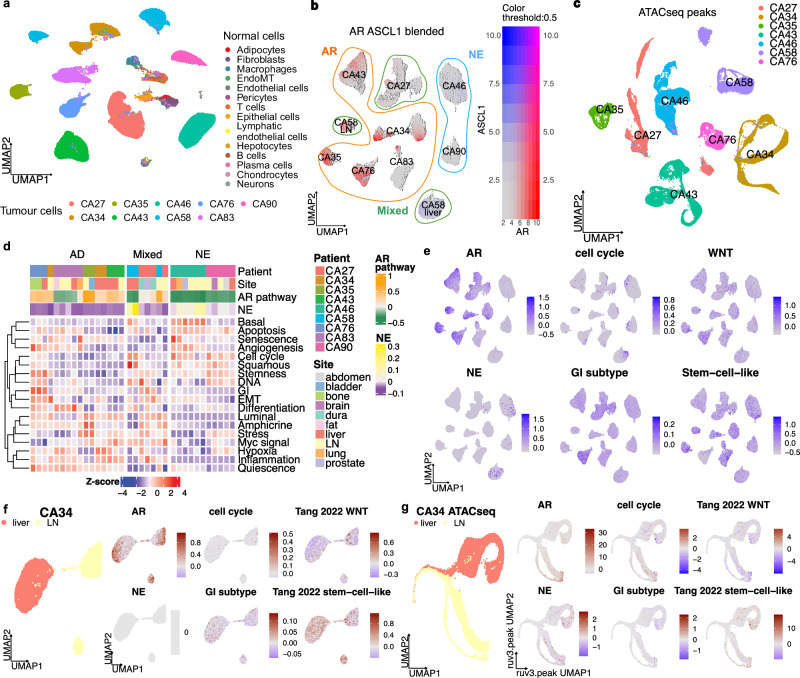
Typical prostate cancer signatures highlight inter-patient heterogeneity in late stage mCRPC. **a** Single-nuclei RNAseq data integrated across all 34 samples (*n* = 185,588 cells). Tumour cells of the same patient cluster together, except for mixed patient CA58, where cells formed two distinct clusters. **b** Expression of *AR* and *ASCL1* across single-nuclei RNAseq data of integrated tumor cells across 34 samples. Cells cluster based on patients and pathologies. There are 78,296 AD cells, 51,639 Mixed cells, 34,791 NE cells. However, multiple sub-clusters are identified for patients CA34, CA83 and CA58. There is large mutual exclusivity between markers. There is no evidence of AR^+^ cells in any of the CA90 and CA46 lesions, despite these patients being initially diagnosed with AD, indicating that only NE cells survived treatment. Both positive *AR* and *ASCL1* are observed in Mixed samples. **c** Single-cell ATACseq data integrated across 24 samples, where available (*n* = 120,708 cells). Similar to the integrated RNAseq data, cells are clustered primarily by patients. Cells from patients CA27 and CA58 separate into multiple clusters, corresponding to different samples. **d** Average expression of mCRPC gene signatures across 34 samples. AR and NE signatures agree with the pathology classification. Signature levels capture inter-patient heterogeneity and were strongly associated with AD and NE classification but are not linked with disease sites. **e** Integrated tumour cells (*n* = 164,726 cells) across the cohort (*n* = 34 samples) showing expression signatures of the AR pathway, NE phenotype, cell cycling, GI subtype and the WNT and stem-cell-like subtypes. There is some intra-patient heterogeneity of AR and NE signatures, including cell populations showing low expression of either AR or ASCL1. **f** Intra-patient gene expression heterogeneity of CA34 (*n* = 3 samples). Distinct clusters can be observed, but distinct phenotypes defined by the signatures do not explain the heterogeneity observed. There are 12,752 cells in two liver and 5555 cells in the LN sample. **g** UMAP of the same three samples of CA34 built based on open peaks and colored by inferred gene activity (*n* = 12427 liver cells, *n* = 5423 cells). Similar heterogeneity is observed in the gene activity comparing to that shown in (**f**).

To characterise transcriptional phenotypes in the tumour ecosystems in our cohort, we integrated the single-nuclei RNAseq data of all samples. Tumour cells comprised over 90% of cells in our dataset, yielding a total of 156,964 tumour cells (Fig. 2a). The tumour cells of the same patient generally clustered together, suggesting that the greatest variability was between patients with no strong effect linked to the organ of origin. In contrast, normal cells clustered according to cell type, independent of patient identity (Fig. 2a).

Focusing on tumour cell composition, we extracted tumour cells and re-integrated the data from all patients for both the RNAseq and ATACseq datasets. A similar clustering by patient was observed in both datasets (Fig. 2b,c). Regional separation of clusters based on pathology classification was evident when using the expression data (Fig. 2b). As expected, AD samples had high *AR* expression, while *ASCL1*, a key transcription factor in the NE phenotype^25^, was present in NE samples (Fig. 1, Fig. 2b, Supplementary Fig. 1). Notably, despite all cells of CA83 expressing weak *AR* without *ASCL1* expression, one cluster had markedly high levels of *AR*, in contrast to CA35, where homogenous high *AR* expression was observed (Fig. 2b, Supplementary Fig. 2). Cells from the liver lesions of CA58 had high expression of *ASCL1* and low *AR*, whereas most cells of the CA58 LN 50 sample had high expression of *AR*, and no *ASCL1* (Fig. 2b, Supplementary Fig. 2, Supplementary Data 1). Interestingly, CA27 cells sat in between AD and NE samples, and included cell populations with high *AR*, low *AR*, high *ASCL1* expression showing intra-tumour heterogeneity (Fig. 2b,e). These patients highlight distinct states of AD/NE intra-patient heterogeneity, where either a particular pathology dominates a lesion (patient CA58 and CA83) or there is intra-tumour heterogeneity of canonical PC markers (patient CA27). We evaluated various established gene expression signatures to further elucidate tumour heterogeneity (Fig. 2d,e, Supplementary Data 2). However, signatures mostly highlighted inter-patient differences with little intra-patient heterogeneity beyond what could be already uncovered by AD and NE signatures, and there was no significant association with disease site, as determined by ANOVA tests (Fig. 2d,e, Supplementary Data 3), consistent with previous findings^26^. Tumour cells from different lesions of the same patient showed limited integration in both the gene expression and chromatin accessibility profiles, indicating significant inter-lesion heterogeneity, captured by both data sources (Fig. 2f,g, Supplementary Figs. 2,3). We often observed subclusters when using the gene expression data, which either contained cells of single lesions, or a mixture of cells belonging to different sites. However, neither signatures from the RNAseq gene expression or from gene activity derived from the chromatin accessibility data could explain this inter-lesion or intra-lesion heterogeneity, nor the organ where the lesion was located (Fig. 2f,g, Supplementary Fig. 2,3). Compared to the level of clustering and number of clusters obtained with the gene expression data, chromatin accessibility showed less heterogeneity, especially between lesions.

**Fig. 3:**
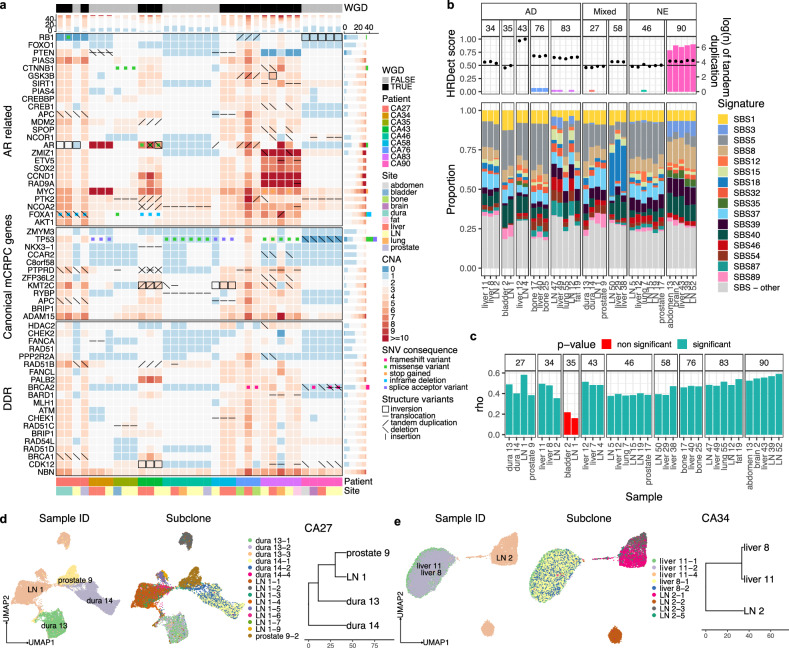
Limited association between transcriptional heterogeneity, genetic clonal evolution and the microenvironment in late stage mCRPC. **a** Oncoplot of somatic SNVs, SVs, and CNVs in mCRPC-related genes of 34 tumours. While inter-patient differences in somatic variants are large, intra-patient differences between lesions are relatively limited. Compared to Mixed and AD tumours, NE tumours have more loss-of-function alterations (e.g. large deletions, STOP gains, frameshifts). Nevertheless, some variants display inter-lesion heterogeneity which may underly transcriptional differences. For example, amplification of *AR* in the CA83 lung sample could be associated with high *AR* expression in this lesion compared to others of the same patient (Supplementary Fig. 2)**. b** Per-lesion HRDetect scores, number of tandem duplication and proportions of COSMIC mutational signature assignments. Lesions of the same patient share similar mutational signature patterns. Patient CA90 has a high HRD score, consistent with the alterations found in the *BRCA2* gene (**a**). The excessive tandem duplication in CA43 can be explained by the mutations on *CDK12* shown in (**a**). **c** Spearman correlation between CN and average expression of the genes of individual samples. All samples, except those of CA35, are significantly correlated, with rho values ranging from 0.36 to 0.60. *P* values were adjusted with Bonferroni method. **d**, **e** Gene expression integration of all samples of patients CA27 (*n* = 20,585 cells: dura = 2 samples of 10,131 cells [6 subclones], LN = 8834 cells [8 subclones], prostate = 1620 cells[1 subclone]) and CA34 (*n* = 16,073 cells: liver = 11,078 cells [4 subclones], LN = 4995 cells [4 subclones]), respectively. The multiple clusters reveal intrapatient, inter-lesion transcriptional heterogeneity. The clustering is consistent with the presence of distinct subclones. The clustering relationship agrees with the distance shown in the phylogenetic trees, e.g. the two dura samples of CA27 (**d**) cluster separately on the UMAP, consistent with a large phylogenetic distance between them; the liver samples of CA34 (**e**) clustered together, and they are phylogenetically closely related. There is additional subclustering of the CA27 LN1 and CA34 LN2 samples, which correspond to further CN subclones within those samples. Rest results for other samples can be found in Supplementary Fig. 7.

We next hypothesised that genetic differences between patients and lesions might drive inter-patient and inter-lesion transcriptional heterogeneity. Analysis of variants in key mCRPC oncogenes and tumour suppressors, along with COSMIC mutational signatures (Fig. 3a,b, Supplementary Fig. 4), indicated minimal intra-patient heterogeneity but considerable inter-patient heterogeneity, aligning with prior findings of monoclonal evolution in mCRPC^27,28^ (Fig. 2d). Since copy number variants (CNV) are known to impact gene expression levels, we assessed the correlation between copy number and binned gene expression (Fig. 3c). Our analysis indicated that there was high correlation between the copy number (CN) of the chromosome segment and the average gene expression of the genes in the segment (rho of 0.36–0.60) in the sample (Fig. 3c). This finding suggests that CN changes play a substantial role in the transcriptional variation observed across different sites and patients. However, our analysis revealed no significant pathway enrichment in the differentially expressed genes (DEGs) of the regions with CN difference between lesions from patients (Supplementary Data 4). This suggests that although CN alterations may contribute to transcriptional heterogeneity, there was no evidence of coordinated upregulation or downregulation of gene expression programs, aligning with the absence of lesion-specific expression signatures.

We next examined the potential of these changes to account for the inter-lesion and intra-tumour transcriptional heterogeneity observed. Lesions from the same patient largely shared the majority of somatic variants, consistent with the derivation from a common ancestral metastatic clone^29^, as can be observed in our phylogenetic tree analysis (Fig. 3b,d,e, Supplementary Fig. 5). Tumours from the same lesions were usually more closely related, such as the two liver lesions in CA34 (Fig. 3e). However, unique sample-specific variants were present and likely arose during the course of metastatic evolution across sites, such as those observed in CA27 (Fig. 3d, Supplementary Fig. 5). We also derived intra-tumour CN subclones with ATAClone^30^, which infers CN subclones from ATACseq data (Supplementary Fig. 6). A CN subclone was defined as a group of cells with the same CN profile and with at least one CN segment different from the other cells in the sample. This approach was preferred over approaches that infer CN from gene expression, given the potential for confounding between gene expression levels and CN levels. In total, 75 subclones were identified across our cohort, with the per-sample subclone number ranging from one (e.g. CA27 prostate 9) to eight (e.g. CA27 LN 1) (Supplementary Fig. 7). While most intra-tumour CN subclones shared mutual variants, cells forming separate clusters were often from a distinct subclone, consistent with a large effect of copy-number on gene expression profiles (Fig. 3d,e, Supplementary Fig. 7)

In summary, we find high correlation between copy number alterations and gene expression changes (Fig. 3c), which resulted in distinct tumour clusters linked with copy-number subclones (Fig. 3d, e, Supplementary Fig. 7, 12), and a lack of functional enrichment analysis of differentially expressed genes between these clusters (Supplementary Data 5). Overall, this suggest that transcriptional heterogeneity is linked to large-scale copy number clonal evolution, consistent with CNVs affecting multiple genes as well are regulatory regions. On a global level, largely contributes to inter- and intra-patient heterogeneity by introducing what could be considered “transcriptional noise”, increasing expression variation to cells, without resulting in the emergence of cell functional states.

### Limited association between the local microenvironment and inter-lesion heterogeneity in advanced mCRPC

**Fig. 4:**
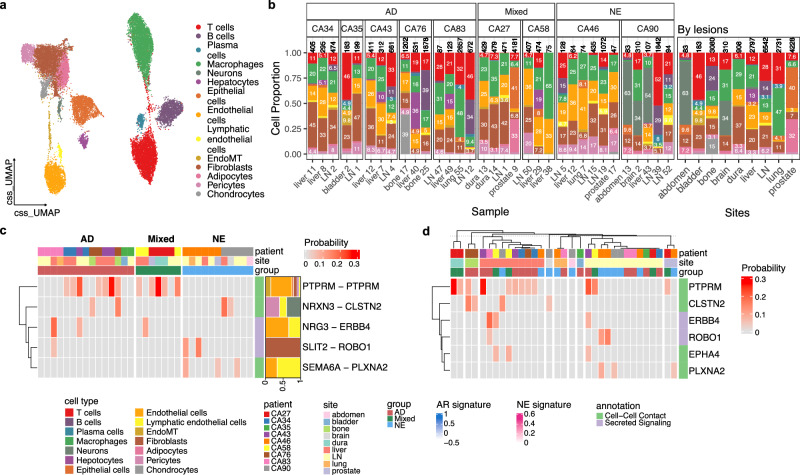
Diverse composition of tumour microenvironments across metastases, with endothelial cells primarily leading the tumour-microenvironment interactions with tumour cells primarily led by endothelial cells. **a** Normal cells (*n* = 36,108 cells; *n* = 33 samples; *n* = 9 patients) across the entire cohort. **b** Normal cell composition across sample (*n* = 33) and organ (*n* = 9) classifications. There is no clear pattern between patients and samples but expected cell types are found in corresponding organs. The same conclusion was found for further normal subtypes (Supplementary Note 1). **c** Ligand-receptor interactions involved in the communication from cells of the microenvironment to tumour cells. Most interactions are found in both adenocarcinoma and NE samples. The average probability of the interaction was taken across cell types in individual samples. The cell types enriched with the interaction are shown as a proportion in the bar on the right. Results with adjusted *p* values < 0.05, probability greater than 0.05 through CellChat’s permutation test, with more than 10 source cells, and recurrent in at least 3 samples and 2 patients are shown. **d** Tumour receptors in the interactions enriched in (**c**) split by site of metastasis. Tumour receptors are expressed across samples, with limited association with organ of metastasis.

Next, we examined the role of the microenvironment of the organ of metastasis in contributing to inter-lesion heterogeneity. We hypothesised that if the microenvironment of the organ drove tumour heterogeneity, there would be transcriptional differences between the metastases located in distinct organs. Nevertheless, the analysis of differential expression across sites, accompanied by pathway enrichment analysis, did not indicate any biologically significant enrichment or signatures unique to the organ of tumour origin (Supplementary Data 6). We also investigated whether patterns could be identified between the cell composition of the microenvironment and pathology, organ or patient. Our dataset comprised 36,108 normal cells, encompassing 14 distinct cell types from immune, stromal, epithelial, and organ-specific categories (Fig. 4a) (Supplementary Note 1). As anticipated, linear modelling showed cell types were enriched according to organ: hepatocytes in liver samples, epithelial cells in lung, B cells in LN and neurons in brain (Fig. 4b, Supplementary Data 7). Even so, the composition of the microenvironment was tied to individual patients. There was enrichment of neurons (ANOVA *p* value = 0.049) in CA90 samples irrespective of organ of metastasis (Fig. 4b). However, the average immune cell proportions between histological classes showed no significant difference, and cell types weakly varied across pathology groups (Supplementary Fig. 8).

To further investigate the microenvironmental modulation on tumour heterogeneity, cell-cell communication was inferred using CellChat, which identifies enriched ligand-receptor (LR) pairs between tumour and non-tumour cells (Fig. 4c, d). This analysis estimates the probability of interactions between LR pairs based on their expression levels on source and receiver cell types^31^. For this analysis, we focused on the signals sent by microenvironment cells (senders) to tumour cells (receivers). We did not find evidence of communication of immune cells with tumour cells; rather, the enriched interactions originated primarily from endothelial cell types (Fig. 4c). We found five significant ligand-receptor interactions enriched between microenvironment cells and tumour cells, where 4 out of 5 originated from endothelial cells. Importantly, the PTPRM-PTPRM interactions were found exclusively in patients with adenocarcinoma cells, while SEMA6A-PLXNA2 were only found in NE samples, suggesting that in end-stage disease, endothelial cells might be playing a role in shaping tumour phenotypes. PTPRM expression in other cancer types has been described as a tumour suppressor associated with reduced tumour migration and proliferation^32^. In this case, the interaction between tumours and mostly endothelial cells with PTPRM could be seen as a malignant strategy to stabilize intra-tumour angiogenesis vessel structure and indicate compact tumour structure of AD tumours as a less aggressive subtype^33,34^. SEMA6A-PLXNA2 has been associated with development of the nervous and vascular system^35,36^, and their downstream activation has been associated to tumour progression, metastasis, proliferation and differentially expressed genes in NEPC^37,38^. This suggests a potential link between neuronal signals from endothelial cells that might promote or support a NE phenotype via SME6A-PLXNA2.

We also investigated whether there were patterns in the tumour cell receptors involved in cell-cell communication that could be linked with organ of metastases (Fig. 4d). We found that the PTPRM receptor on tumour cells was more commonly found on liver samples (6/10) rather than LN samples (2/10) and was found in both dura samples of patient CA27. Endothelial cells were the most common cell types in livers (Fig. 4b). Thus, the enrichment of communication through PTPRM in livers could be linked to the enrichment of endothelial cells in this organ and their communication with AD cells, as shown in Fig. 4c.

Collectively, these findings suggest that the composition of the local microenvironment show no clear patterns linked with the organ of metastasis or with tumour phenotype. Furthermore, the crosstalk between microenvironment prostate tumour cells was rare and inconsistent between patients, while limited to primarily endothelial cells, without strong associations to the organ of metastasis. Even so, there was some evidence that communication of the microenvironment is closely tied to tumour pathology.

### Recurrent intra-tumour transcriptional heterogeneity revealed by archetype analysis

**Fig. 5:**
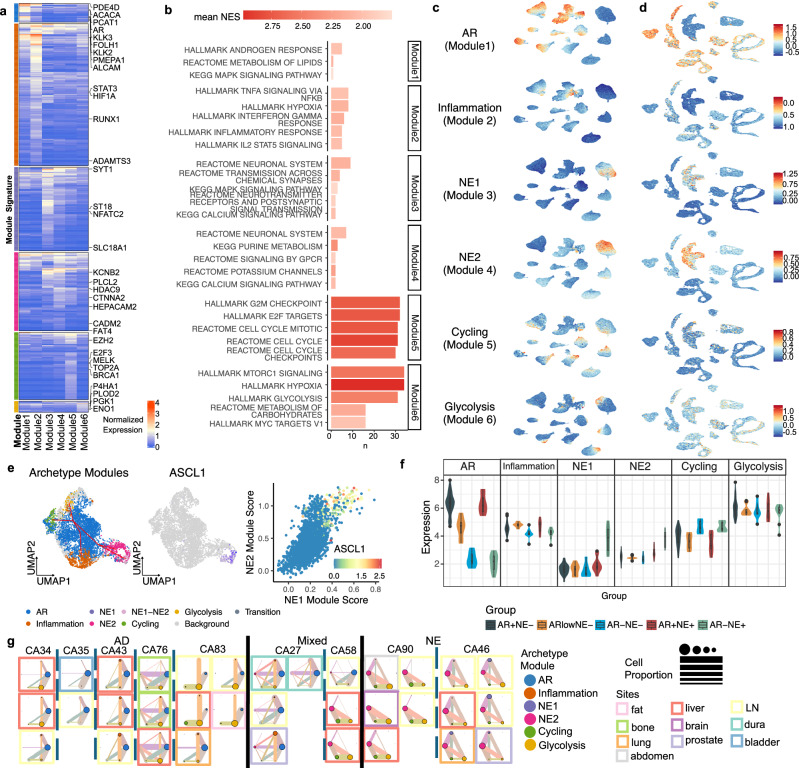
Archetype tumour signatures capture intra-tumour heterogeneity of mCRPC. **a** Heatmap of gene expression of the six modules derived from archetype analysis in tumour cells of 9 patients (n = 164,726 cells). **b** Pathway enrichment of archetype module signatures by GSEA of differentially expressed genes between cells expressing module signatures. **c** Expression of archetype module signatures in tumour cells (*n* = 164,726 cells). These signatures capture different populations of cells within lesions, enabling the dissection of inter-lesion and intra-lesion heterogeneity. **d** The gene activity of the corresponding module signatures inferred from ATACseq data integrating tumours from 7 patients (*n* = 24 samples, *n* = 120,708 cells). **e** Plasticity of NE is shown by NE1 and NE2 signature through pseudotime analysis in the CA27 dura 13 sample (*n* = 4,470 cells). Co-expression of NE1 and NE2 is the most distinct state relative to the AR Module population and agrees with *ASCL1* expression. **f** Expression of six module signatures in the CRPC bulk RNA expression study from Labrecque et al. with samples stratified into five classes based on AR and NE marker expression into AR + NE-(*n* = 360), ARlowNE-(*n* = 54), AR + NE + (*n* = 66), AR-NE + (*n* = 60). Archetype modules show similar expression trends, except for a higher prevalence of the Inflammation Module in a broader cohort and still showed its role as a marker for low AR-level tumour cells. NE1 and NE2 highlighted NE populations, while NE2 outperformed in the AR + NE+ class, and NE1 seemed more prevalent in the AR-NE+ class. The median is shown by the centre line of box plots. The box spans from the 25th to the 75th percentile. The whiskers extend to the most extreme values within 1.5 × IQR of the box hinges. Outliers are plotted individually beyond the whiskers as black dots. **g** Hexagon plot of cell proportions assigned to each module or transition state; node size and edge width are scaled to the proportion of cells per module or transitioning between modules. There is limited intra-patient heterogeneity, with all metastases of individual patients having a similar pattern in the distribution of cell populations. An exception is patient CA58, whose liver samples resemble the patterns found in patients with NE disease, while the LN sample resembles other AD samples.

Given the limited role of clonal evolution and the microenvironment in explaining mCRPC heterogeneity, we aimed to de novo identify tumour cell states. Archetype analysis was utilized to derive signatures of cell populations with most extreme phenotypes^39,40^. We identified a total of six archetype modules across samples (Fig. 5a-c, Supplementary Data 8). Module 1 (AR Module) included *AR*, *AR* regulators and downstream genes, and lipid metabolism genes (Fig. 5a), with an enrichment for androgen response and lipid metabolism pathways and a high correlation (rho = 0.82) with previous established *AR* signatures (Fig. 5b, Supplementary Fig. 9). Module 2 (Inflammation Module) was enriched in TNFa signaling through the NFkB pathway, hypoxia, interferon-gamma pathway and IL2-STAT5 interaction (Fig. 5a, b). Two modules, modules 3 and 4 (NE1 and NE2 Modules) included previously known NE markers (Fig. 5a), were positively correlated with *ASCL1* expression in NE samples (NE1 rho = 0.272, NE2 rho = 0.104) (Supplementary Fig. 9) and were enriched with signatures associated with neuronal signaling (Fig.5b). The NE2 signature included more genes involved in EMT through their roles in cell adhesion, migration, and extracellular matrix remodeling (eg. *CADM2*, *CTNNA2*, *FAT4*), suggesting a role in driving NE plasticity (Fig. 5a). Module 5 (Cycling Module) included typical cycling markers (Fig. 5a, b). Finally, Module 6 (Glycolysis) was a metabolic module complemented by mTORC1, glycolysis, HIF-a hypoxia-related genes, and *ENOS1* and *RAB1A*, which are related to EMT^41,42^ (Fig. 5a, b). The motif enrichment in the subpopulations with representative expression of this module signatures agreed with the functional enrichment found above, e.g. E2F family for Cycling Module, *FOS* and *JUN* family binding in Glycolysis and Inflammation Module, *HOX* family for AR Module, as well as typical active regulators like *NEUROG1*, *ASCL1*, *BHLHE22* for NE Modules (Supplementary Data 9).

Overall, 55.6% of tumour cells were assigned to a single archetype module (Fig. 5, Supplementary Fig. 10), while 30% could be assigned to multiple modules, and were considered to be in transitional states between those states (Fig. 5c, Supplementary Fig. 10). The data from cells assigned to a single module or a transitional state were of similar quality (Supplementary Fig. 11). Cells from the Cell Cycle Module constituted 0.03% to 15.4% of cells in each sample, but were more common in NE samples (mean = 3.9%) than AD samples (mean=0.5%) (one-sided Wilcoxon *p* value = 1.5 × 10^−6^), consistent with previous studies^43,44^ (Fig. 5c, Supplementary Fig. 10). The Glycolysis Module was the most prevalent module across all samples and was highly expressed in 10.2% of tumours cells (Supplementary Fig. 10). All AR-low CA83 samples were predominantly influenced by this module, while the lung lesion, marked by elevated *AR* expression, exhibited reduced levels (Fig. 2c-e, Supplementary Fig. 2). These findings are consistent with previously described changes in glycolysis associated with decreased *AR* expression^17,45,46^. The gene activity inferred from the matched open chromatin data showed strong correlation to the gene expression results except for the Cycling and Glycolysis Modules (Fig. 5d and Supplementary Fig. 9), which is consistent with the dynamic regulation of metabolites and cell cycle modulators on chromatin accessibility^47–49^.

Both NE1 and NE2 were expressed in NE samples (CA46, CA90 and liver lesions from CA58), however, the NE2 Module was more broadly expressed, with expression in all tumours of Mixed phenotypes including amphicrine cells (*AR*^+^*ASCL1*^+^), some AD tumours including from CA35 and CA76 (Fig. 4c,d, Supplementary Fig. 5). In contrast, NE1 was more co-expressed with *ASCL1* expression (Supplementary Fig. 10), suggesting its expression in cells already exhibiting NE phenotype. Pseudotime analyses of the dura13 of CA27 revealed that co-activation of both signatures represented a more differentiated state during NE transition, with multiple genes associated with the transition between NE1 and NE2 (Fig. 5e, Supplementary Note 2). High expression of the NE2 signature was also detected in a small subset of cells in the primary tumours of CA90, potentially suggesting that NE2 could be an early signature of NE development potentially supporting the development of metastasis (Supplementary Fig. 12). Thus, NE1 and NE2 describe different stages of the development of the NE phenotype, with NE2 being more likely to capture cells earlier or less committed to the NE transition (Supplementary Note 2). Similar trends were found in independent human and pre-clinical datasets (Supplementary Note 3).

To understand the expression of the archetype modules in larger mCRPC cohorts, we investigated their expression in previously published data^50^ (Fig. 5f), which stratified 98 mCRPC tumours into five subtypes: AR + NE-, ARlowNE-, AR-NE-, AR + NE+ and AR-NE + . As expected, our AR Module showed highest expression in AR + NE- samples, followed by that in AR + NE+ group (pair-wise Wilcoxon test p value all significant and below 1.2 × 10^−4^) (Fig. 5f). The Glycolysis Module was ubiquitously highly expressed, AR-NE- and *AR*-NE+ tumours showed the highest expression of the Cycling Module (pair-wise Wilcoxon test p value all significant and below <0.041) and the Inflammation Module was more highly expressed in ARlow tumours (pair-wise Wilcoxon test p value all significant and below <0.024) (Fig. 5f). NE1 and NE2 were the highest expressed in AR-NE+ tumours (pair-wise Wilcoxon test p value all significant and below <2.2 × 10^−5^) (Fig. 5f), consistent with the NE2 signature being linked to the early stages of NE transition, without yet being committed.

Our results suggest that the inter-lesion and intra-tumour heterogeneity in mCRPC is defined by six cell states with distinct transcriptional signatures, including signatures associated with early stages of lineage plasticity to NE disease.

### Metastases develop predictable patterns of intra-tumour transcriptional heterogeneity

We next reasoned that the observed intra-lesion heterogeneity could be the result of spontaneous transcriptional changes that offer little survival advantage, or it could be a fundamental process of tumour development. For the former, we would anticipate the presence of the transcriptional modules to show no pattern across tumours, whereas for the latter, we would anticipate recurrent patterns of intra-tumour heterogeneity. We therefore investigated the proportion of cells across our samples that belonged to each archetype module, as well as cells transitioning between two or more module signatures (Fig. 5g). Surprisingly, the proportion of cells belonging to the distinct cell states and transitioning was recapitulated across samples of individual patients. An example is the four lesions from patient CA27, where each developed NE disease across metastases, regardless of lesions and subclonal background (Supplementary Fig. 13), and the samples from other patients showed emergence of at least the other four modules at similar proportions (Fig. 5g). This observation indicates that metastases have the capability of independently and predictably developing patterns of intra-tumour heterogeneity.

Common patterns of intra-tumour heterogeneity across patients were identified (Fig. 5g). CA58 LN, CA76, and CA27 samples had a high prevalence of all modules with rare exceptions exhibiting NE1 and/or NE2 expression and transitioning cells, whereas CA34, CA35, and CA43 were primarily enriched with Inflammation, Glycolysis and *AR* modules. In contrast, NE tumours had a high prevalence of NE1 and NE2, Cycling and Glycolysis signatures, including transitioning cells. We also identified three intra-tumour heterogeneity patterns in samples with NE features: exclusive expression of either NE1 or NE2, or co-expression of both. CA46 showed the most exclusive expression of NE1 and NE2, while in the rest of the NE samples, co-expression of both with larger NE2 prevalence was more common (Supplementary Fig. 2, Supplementary Note 2).

### Recurrent tumour modules develop independently from clonal evolution and remodel the microenvironment

We subsequently investigated whether the emergence of cells with signatures of the archetype modules could be linked to subclonal evolution, as we had previously shown a strong association between CN subclones and transcriptional heterogeneity (Fig. 3).

**Fig. 6:**
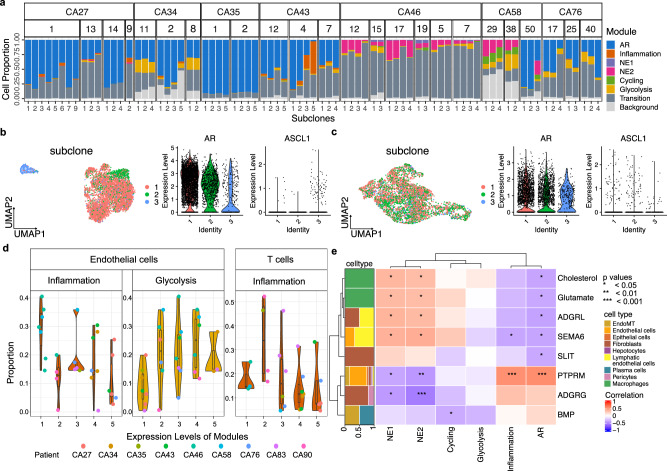
Recurrent intra-tumour heterogeneity is independent of subclonal evolution and has a remodelling effect on the TME. **a** Archetype module populations found in subclones. All subclones include a mixture of cell populations, with a similar proportion of cell populations across subclones of individual samples and patients. **b** Expression of *AR* and *ASCL1* in CA58 LN 50 subclones (*n* = 5431 cells: subclone 1 *n* = 4115, subclone 2 *n* = 883, subclone 3 *n* = 433). **c** Expression of *AR* and *ASCL1* in CA27 dura 13 subclones (*n* = 4423 cells: subclone 1 *n* = 2366, subclone 2 *n* = 1742, subclone 3 *n* = 315). For the former, *ASCL1* expression is largely confined to subclone 3, but for the CA27 dura 13 sample, there are cells highly expressing *ASCL1* in all subclones. **d** Association between the average level of expression of the archetype module signatures and the proportion of normal cells in the samples. Only significant results (*p*-value < 0.05) from the one-sided Jonckheere-Terpstra (JT) test are shown. Cell types were included in tests only when more than 10 cells present in certain samples. The proportion of endothelial cells decreases as the expression of the Inflammation Module increases (JT test *p* = 0.008, 29 samples), but their proportion increases as the Glycolysis Module increases (*p* = 0.0218, 29 samples). The proportion of T cells decreases as the Inflammation Module increases (*p* = 0.0428, 25 samples). The median is shown by the centre line of box plots. The box spans from the 25th to the 75th percentile. The whiskers extend to the most extreme values within 1.5×IQR of the box hinges. Outliers are plotted individually beyond the whiskers as black dots. **e** Spearman correlation between the probability score of enriched cell-cell communication pathways (in 30 samples of 9 patients) from microenvironment to tumours and the average expression of the module signatures. The cell types involved in each enriched the pathway are shown the left bar. The microenvironment communicated more actively with the AR, Inflammation and NE1/NE2 Modules than with the Cycling and Glycolysis Module. There are opposite patterns of communication between the AR, Inflammation Modules compared to the NE1/NE2 Modules.

Most subclones contained a mixture of cells from different archetype modules, indicating that their evolution was not tied to the genetic evolution of clones, but rather similar transcriptomic programs derived through other mechanisms (Fig. 6a). Using a linear mixed effects models, we found that the variability in the expression of the module signatures showed only limited association with the underlying subclone (percentage of variance explained is estimated to be 1.6% for the NE2 module, 4.2% for the Glycolysis module, 5.5% for the AR module, 11.2% for the NE1 module, 12.2% for the Inflammation module and 28.2% for Cycling module). Only in one case did we find a clear transcriptional phenotype linked to a unique clone (Fig. 6b). Here, the LN50 sample of CA58 had a subclone dominated by the expression of *ASCL1* and NE signatures in all cells, whereas the other two subclones of the samples only included AR + NE- cells (Fig. 6b). However, none of the typical NE signature genes (*ASCL1*, *INSM1*, *SOX2*, *SYP*, *CHGA*) were within the copy number regions exclusively amplified in the NE subclone, implicating other mechanisms, likely epigenetic, that lead to the activation of NE signatures tied to a specific clone. Another example is CA27 dura13, where all three subclones spontaneously experienced NE differentiation (Fig. 6c). Therefore, transcriptional intra-tumour heterogeneity predominantly arises independently of clonal evolution. Nonetheless, specific cellular states may be associated with distinct clones, even in the absence of genetic determinants.

Considering the microenvironment as a possible driver of intra-tumour transcriptional heterogeneity, we found higher expression of the Inflammation Module was associated with a lower proportion of endothelial cells and T cells (Fig. 6d), whereas Glycolysis Modules were associated with increased endothelial cell proportion, consistent with endothelial cells enabling a tumour immune-suppressive and hypoxic local microenvironment^51,52^. This is consistent with more blood vessel invasion in proliferating, metabolically active and hypoxic tumours but less in inflammatory tumours^6,51^. In addition, analysis of each immune population separately identified multiple B, T and macrophage subtypes, but did not identify further patterns (Supplementary Note 1). Correlation of the average module expression with cell communication pathways revealed that Modules had mutually exclusive associations with these. The expression of the Inflammation and AR Modules was positively and significantly correlated with PTPRM pathway expression. However, the expression of NE1/NE2 Modules was positively correlated with a series of synaptic adhesion (ADGRL, SEMA6), development (Cholesterol) and signaling (Glutamate) cell communication pathways. These “neuronal-like” signals from the microenvironment (Fig. 6e) were consistent with our results investigating cell-cell communication at the sample-level (Fig. 4c). Collectively, our results indicate that the recurrent cell-state patterns observed across metastases of individual patients are likely enabled by shaping of the microenvironmental composition by the tumour, enriching for stromal populations, primarily endothelial cells, that support, through cell-cell communication, the distinct tumour cell phenotypes.

In general, our findings suggest a model where patterns of intra-lesion transcriptional tumour heterogeneity are shared across metastases within an individual patient and even across patients, is independent to clonal evolution, and drives remodelling of the tumour microenvironment.

### Expression of therapeutic tumour receptors linked to transcriptional intra-lesion heterogeneity

Intrinsic resistance to new targeted therapies for metastatic disease contributes to disease lethality. This study provides a unique opportunity to investigate the role of clonal evolution, the microenvironment and plasticity when intra-tumour heterogeneity modulates treatment resistance. Receptors on the tumour surface have enormous therapeutic potential as they can be used as targets for CAR-T cells, Antibody-Drug Conjugates, Bi-specific T-cell Engagers and radioligand therapy. ROR1, STEAP1/2, B7-H3 are actively tested in clinical trials^53–55^. Lutetium-PSMA, a radioligand therapy approved by the FDA in 2022 for the treatment of mCRPC^56–58^, targets the highly expressed tumour surface marker Prostate Specific Surface Antigen (PSMA), delivering cytotoxic radiation to eliminate tumours cells^59^. Expression heterogeneity of these targets often leads to reduced therapeutic benefit due to treatment resistance^59,60^. Therefore, understanding the underlying drivers of the heterogeneity of these targets is critical for the development of strategies to increase treatment access and extend durability.

**Fig. 7:**
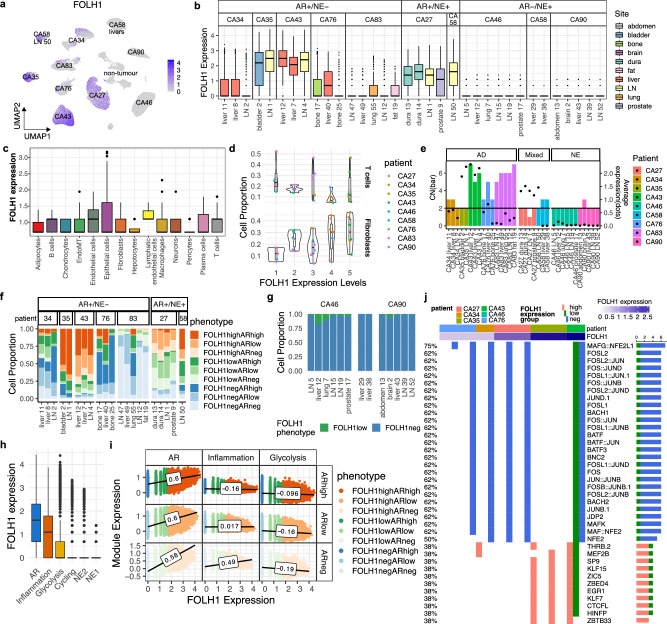
Recurrent intra-lesion expression heterogeneity of RLT targets. **a** FOLH1 expression across the tumour and normal cells (*n* = 185,588 cells, *n* = 34 samples) shows diversity between AD patients, with near-negative expression in NE samples. **b** High FOLH1 expression is commonly seen in liver and LN tumours (*n* = 34 samples; *n* = 9 patients), particularly in AR + /ASCL1− samples. **c** FOLH1 expression in microenvironmental cells (*n* = 36,108 cells; *n* = 33 samples; *n* = 9 patients) is rare, with limited expression in epithelial and endothelial cells. **d** T cell and fibroblast proportions were negatively and positively associated with FOLH1 expression, respectively (one-sided Jonckheere-Terpstra test; *p* = 0.037, *n* = 24; *p* = 0.014, *n* = 29). **e** FOLH1 copy number (CN) gains or deletions do not consistently alter expression levels across samples (*n* = 34), except in CA43. Horizontal line indicates diploid state. **f** The proportion of tumour cell types based on AR and FOLH1 expression is similar across patient lesions. **g** A small subset of NE cells shows low FOLH1 expression. **h** FOLH1 levels decrease across archetype populations of 9 patients: AR (*n* = 83,883), Inflammation (*n* = 10,726), Glycolysis (*n* = 40,890), Cycling (*n* = 15,766), NE2 (*n* = 34,140), and NE1 (*n* = 13,214). Pairwise two-sided Wilcoxon test *p* values: 1.7 × 10⁻²⁰⁴, ~0, 6 × 10⁻²²⁷, 7.7 × 10⁻¹⁵⁶, 1.3 × 10⁻²⁴, respectively. **i** Spearman correlation between FOLH1 and AR (*n* = 83,883 cells), Inflammation (*n* = 10,726 cells), Glycolysis (*n* = 40,890 cells) module scores under different AR contexts of 9 patients. The AR Module positively correlated with FOLH1 regardless of AR levels; the Inflammation Module correlated positively only in AR− cells; Glycolysis showed no consistent correlation. *P* values were Benjamini-Hochberg adjusted (see Source Data). **j** Motifs associated with differentially opened chromatin peaks between tumour cells with high, low, or negative FOLH1 expression within individual tumours. Significant motifs are found in 10 samples from 5 patients. FOLH1 expression status was determined by mean gene expression; negative cells had zero FOLH1 counts. Box plots show median (centre line), interquartile range (box), values within 1.5×IQR (whiskers), and outliers (black dots).

*FOLH1* expression, which encodes PSMA, was observed in both tumour and normal cells. Tumour expression varied between and within patients in our cohort (Fig. 7a), with no organ-specific expression patterns (Fig. 7b). We also observed expression of *FOLH1* in endothelial types, and rarely in epithelial cells (Fig. 7c). Tumour *FOLH1* levels increased with increasing fibroblast proportion (one-sided JTtest *p* value = 0.014) but with decreasing the proportion of T cells (one-sided JTtest *p* value = 0.037) (Fig. 7d). Nineteen samples from five patients had SNPs on FOLH1, but they occurred in intron regions, except for the missense variant rs75111588 in samples from CA46 and CA76 (Supplementary Data 10), but there was an unclear association between this variant and *FOLH1* expression (Supplementary Data 11). Most samples were diploid at the *FOLH1* locus, and samples with a *FOLH1* CN gain did not consistently show a higher *FOLH1* expression (Fig. 7e), even when considering intra-lesion clonality (Supplementary Data 11). No other structural variants involved *FOLH1*. Overall, genetic changes in the gene body of *FOLH1* have a limited association with *FOLH1* expression.

*FOLH1* levels showed great intra-tumour heterogeneity. Annotation of cells based on *FOLH1*, *AR* and *ASCL1* expression revealed that the proportion of cells in each phenotype was similar between sites of the same patient, with the largest variation being between patients (Fig. 7f, g), consistent with our model of recurrent intra-lesion heterogeneity. *FOLH1* expression also was tightly associated with our archetype modules (Fig. 7h). AR-module cells had the highest *FOLH1* levels (Fig. 7h). However, while there was a positive correlation between the *AR* gene and *FOLH1* (rho=0.48), high *AR* did not always correspond to high *FOLH1*, consistent with previous studies^8^ but the AR Module was more correlated (rho = 0.58–0.6) with *FOLH1* regardless of *AR* expression (Supplementary Fig. 14, Fig. 7i). This showed *AR* still has the strongest positive modulation of *FOLH1*. Importantly, the Inflammation and Glycolysis signatures were associated with lower *FOLH1* levels (Wilcoxon *p* value < 2.2 × 10^−16^) (Fig. 7h). Interestingly, for the Inflammation module, this association was found primarily in *AR* high cells, while for the Glycolysis module, downregulation effect on FOLH1 is the most in cells without *AR* (Fig. 7i), potentially consistent with distinct or complex underlying mechanisms for reduced *FOLH1* expression even within individual lesions. The expression of other tumour receptors of therapeutic interest, namely, *STEAP1/2, ROR1, and CD276 (B7H3)* was also associated with archetype module expression (Supplementary Fig. 14, 15). While *FOLH1, STEAP1/2* and *CD276* showed lower expression in the Glycolysis and Cycling Module high populations, *ROR1* showed medium levels of expression in these cell populations and in NE2 cells, suggesting that targeting of *ROR1* would be more effective across a broader range of tumours. *STEAP2* had a similar pattern of expression to *FOLH1*, but it was more highly expressed in the Glycolysis populations, also suggesting a broader utility (Supplementary Fig. 14).

We next investigated potential transcription factors associated with *FOLH1* expression using our chromatin accessibility data (Supplementary Data 12). We performed differential peak analysis followed by motif enrichment between *FOLH1* high, low and negative subpopulations within individual tumors (Fig. 7j). The AP-1 superfamily of transcription factors, including JUN, FOS, MAF and BATF, showed active roles in cells expressing low or no *FOLH1* (Fig. 7j). AP-1 activity plays critical roles in cell proliferation, immune regulation and stress responses during tumorigenesis^61–64^, consistent with archetype modules (Glycolysis and Inflammation module) associated with *FOLH1* downregulation (Fig. 7h-i, Supplementary Data 9). Upregulated *FOLH1* expression was associated with the *CTCFL* and *KLK* families of transcription factors, which are linked with androgen receptor regulation in previous studies^65,66^.

In conclusion, transcriptional plasticity was a major driving force associated with PSMA heterogeneity in mCRPC. The development of similar patterns of *FOLH1* heterogeneity across lesions of a patient suggests that response to LuPSMA is likely to be determined early in tumour evolution. Early clinical testing that captures archetype module expression could be a strategy to optimize patient selection for LuPSMA.

## Discussion

This study examines the impact of genomic and non-genomic evolutionary factors on transcriptional heterogeneity in mCRPC. We identified ample intra-patient heterogeneity, with transcriptional plasticity, the microenvironment and genomics differentially contributing to the development of this heterogeneity.

Our study suggests that intra-tumour heterogeneity is a highly regulated process in the tumour ecosystem. We identified six tumour archetype transcriptional gene modules related to *AR*, neuroendocrine development, cycling cells, inflammation, glycolysis and hypoxia. While other studies have identified modules with similar functions across cancers^4,39,67–70^, our work using multiple metastases per patient enabled identifying mCRPC-specific genes involved in these processes. This enabled detailed cell phenotyping of intra-tumour mCRPC heterogeneity, the characterisation of the recurrent composition of tumour transcriptional cell types across metastases of patients, as well as the description of how this composition varied across patients.

Our study demonstrates that across metastases of individuals, not only are tumour cell states stable, but their cell proportions are retained regardless of organs and clonal background. The spontaneous and recurrent ‘division of labour’ between tumour cells points towards the essential elements of the tumour ecosystem for tumour viability and treatment resistance. The optimal combination of ecosystem components is therefore likely tied with intrinsic tumour features acquired early in the disease evolution of the metastatic clones, which results in the observed convergence of intra-tumour phenotypic heterogeneity across metastases. This convergence is also likely shaped by the treatment histories of the patients, and will ultimately influence treatment resistance.

Our findings indicate that inter-lesion and inter-patient transcriptional heterogeneity is shaped by the expression changes linked to large-scale copy-number alterations. This could be considered ‘transcriptional noise’ as it does not contribute to the emergence of functional cell states. On the other hand, we found that in advanced metastatic disease, while immune components played a minor role in shaping transcriptional heterogeneity, there was evidence consistent with a reciprocal remodelling occurring between tumour and stroma cells. The microenvironment has been proposed as a factor influencing tumour heterogeneity^71,72^ and remodelling of the microenvironment in the development of pre-metastatic niches is well established^6,51,52,69,71^. However, our study shows that the contribution of the microenvironment in shaping late-stage disease is likely more limited but could still act as a support to tumour heterogeneity. The absence of organ-specific tumour signatures and the presence of recurrent intra-tumour heterogeneity across metastases, regardless of organ, suggest that tumour evolution, at this advanced disease stage, occurs largely independently of the initial local microenvironment of the organ. However, our results also show enrichment of endothelial cells and cell communication signals from the microenvironment closely tied to tumour pathology and tumour cell signatures, suggesting co-evolution and remodelling of tumour and stroma cells that supports intra-lesion heterogeneity. Treatment strategies that aim to modulate the microenvironment should therefore consider the underlying properties of the tumour. For example, anti-angiogenesis treatments in patients showing high Glycolysis Module signatures or targeting key cell-cell interactions with endothelial populations.

Understanding the driving forces of NE disease is critical for developing markers for early detection. Clinically, mCRPC that behaves as NE but retains AD morphology has been extensively reported^73^, however, its timely identification and treatment remain challenging. We uncovered two signatures of NE disease, one aligned with canonical NE markers and the other associated with earlier transitional states, delineating distinct stages of NE differentiation^74–77^. Our NE2 signature was found in a subset of patients with AD morphology and lower *AR* levels but without canonical NE markers or small-cell morphology, indicating that the adoption of NE-like features can occur without the adoption of markers used clinically and without eventual transition to NE. Therefore, our signature of early NE transition may represent a promising molecular signature for the identification of these patients. Of note, we found this signature in tumour cells of a primary AD sample from a patient who later developed NE disease, indicating that this signature could potentially be used in early-stage disease to identify patients at higher risk of developing NE mCRPC, potentially offering a window for earlier intervention.

Interestingly, we also identified that NE disease can reoccur across different clones within and between lesions, while others in other cases it can be tied to particular clones but not linked to acquired genetic variants, consistent with previous findings^78^. This suggests that some prostate cancers are intrinsically more plastic, leading to the recurrent development of NE disease, while in others, the development of NE disease might have been a single event that gets tied with a single clone, which then spreads and dominates the tumour landscape.

Finally, our findings open exciting opportunities for the prediction of patient treatment response early in the history of the disease based on transcriptional-level biomarkers, as well as classification systems of patients based on their tumour ecosystem composition. For example, we identified significant intra-tumour heterogeneity of *FOLH1* and other therapeutic targets linked with these tumour ecosystem components, indicating that early characterization of the composition of the tumour ecosystem might lead to more refined stratification of patients for emerging treatments. Studies classifying intra-tumour tumour ecosystems across large cohorts of patients, their association with treatment response, as well as how treatment resistance shapes these ecosystems, will be instrumental in driving better treatment options for prostate cancer patients.

Given the limited number of patients, we have focused our analysis on in-depth intra-patient tumour evolution across multiple lesions, and therefore our results are best interpreted in that context rather than in terms of broad inter-patient generalizability.

Overall, our study provides unique insight into late-stage mCRPC and has allowed uncovering key aspects of tumour evolution. Studies investigating how intra-lesion heterogeneity develops at earlier stages of metastases would shed further light on the history of tumour ecosystem convergence across metastases.

## Methods

### Ethics approvals

Metastatic lesions of mCRPC patients were selected from 9 patients from the CASCADE rapid autopsy program set up at the Peter MacCallum Cancer Centre^23^, with approval by the Human Research Ethics Committee of the Peter MacCallum Cancer Centre (HREC 11/102 and 15/98). A written informed consent was obtained from all patients participating the program. Metastases were collected 2–12 h after death and processed as fresh frozen and FFPE samples.

### Sample selection

Samples selected for profiling in this study had at least 50% tumour content and the high quality of individuals based on histology assessment of FFPE H&E slides. We aimed to maximize the diversity of organ representation and biomarker immunohistochemistry (see below Pathology review section) in each patient. In total, 34 samples from multiple sites of nine patients were selected for this study.

### Pathology review

A summary of pathology reviews, including purity quantifications and staining results is presented in Supplementary Data 1. H&E slides were reviewed by a pathologist, and the percentage of tumour, necrosis and fat was assessed and scored as 0, <1, <5, 10, 20, 30 up to 100% (in increments of 10%) of the total tissue area. Only slides with tumour purity >50% were included in the study. Immunohistochemical (IHC) stain was completed to show the PSMA, AR, INSM1, SYN, CGA levels of samples using Anti-PSMA Monoclonal Mouse Antibody (clone 3E6 from Dako with 1:50 dilution, stained by Ventana Discovery Ultra Automated Stainer from Ventana Medical Systems Inc., Oro valley, United States); Monoclonal Mouse anti-Human Androgen Receptor (clone AR41 from Dako with 1:50 dilution, stained by Bond RX Automated Research Stainer); INSM1 (clone A-8 from Santa Cruz Biotechnology sc-271408 with 1:400 dilution, stained by Ventana Benchmark Ultra platform, Ventana Medical Systems, Tucson, AZ); SYN: 1:50 (Ventana Benchmark Ultra platform, Ventana Medical Systems, Tucson, AZ); Monoclonal Mouse Anti-Human Chromogranin A (clone DAK-A3 from Dako with1:400 dilution, stained by Ventana Benchmark Ultra platform, Ventana Medical Systems, Tucson, AZ), respectively. The intensity and percentage of AR, INSM1, SYP, CGA positive IHC stain were quantified by a senior pathologist specialising in prostate cancer (Supplementary Data 1). H scores were calculated using the formula:

1 $${{{{\rm{H}}}}}_{{{{\rm{score}}}}}=\frac{{{{\rm{intensity}}}}\times {{{\rm{percentage}}}}}{{{\rm{max}}} \; {{\rm{score}}}=300}$$

IHC staining for CD3, CD8, CD11c, CD68, CD20 and HLA-ABC was performed for the entire cohort. The above stain was completed using the following antibodies respectively: Rabbit monoclonal [SP7] to CD3 (clone SP7, 1:500 dilution, Abcam, catalogue no. ab16669), Mouse Monoclonal CD8 (clone 4B11, 1:150 dilution, Invitrogen, catalogue no. MA1-80231), Rabbit monoclonal to CD11c - C-terminal (clone EP1347Y, 1:400 dilution, Abcam, catalogue no. ab52632), Mouse Monoclonal CD68 (clone 514H12, 1:100 dilution, Thermo Fisher Scientific, catalogue no. MA180133), Mouse Monoclonal CD20 (clone L26, 1:4000 dilution, Agilent Technologies, catalogue no. M075501-2), Mouse Monoclonal anti-HLA Class 1 ABC antibody (clone EMR8-5, 1:1000 dilution, Abcam, catalogue no. ab70328). Whole slide scans were obtained on a VS120 Slide scanner (Olympus) at 20x magnification. Scanned images were opened in the CellSens Image Analysis software (Olympus) and a region of interest (ROI) was created around the tissue area. Cells were then thresholded for brown ( + ve) cells and purple (all cells/tissue), enabling the determination of the percentage of positive (brown) cells as a percentage of the total (purple) area.

### Sample preparation and data generation

All metastatic samples used for single-nuclei preparation were fresh-frozen. Samples from CA27, CA34, CA35, CA43, CA46, CA58, and CA76 were processed for 10X Chromium multiome (ATAC+gene expression), CA83 and CA90 for 5’ Single Nuclei RNA Sequencing. For the primary samples of CA90, nuclei were isolated using the SnPATHO-seq protocol. Nuclei isolation and library generation were performed by the Molecular Genomics Core (MGC) at the Peter MacCallum Cancer Centre. Sequencing was performed in either MGC or the Australian Genome Research Facility (AGRF) as specified below.

### Multiome (ATAC+ gene expression) protocol^79^

#### Gradient solution preparation

Before tissue processing, the gradient solutions (GD, GH, GW, and G30) were prepared. GD consists of 150 mM KCl, 30 mM MgCl2, and 120 mM Tricine-KOH at pH 7.8; GH consists of 0.25 M sucrose, 25 mM KCl, 5 mM MgCl2, and 20 mM Tricine-KOH at pH 7.8 (kept at 4 °C); and GW is prepared by mixing 5 volumes of OptiPrep® (60% (w/v) solution of iodixanol in water) with 1 volume of GD to achieve a final concentration of 50% iodixanol, 25 mM KCl, 5 mM MgCl2, and 20 mM Tricine-KOH at pH 7.8. Gradient Solution G30 was prepared by mixing 6 volumes of GW with 4 volumes of GH, resulting in a final concentration of 30% iodixanol, 25 mM KCl, 5 mM MgCl2, and 20 mM Tricine-KOH at pH 7.8.

#### Nuclei preparation

Snap-frozen samples were cut into 100 µm sections. Tissue pieces were manually homogenized using a Douncer in chilled salty EZ lysis buffer (10 mM Tris-HCl at pH 7.5, 146 mM NaCl, 1 mM CaCl2, 21 mM MgCl2, 0.03% Tween-20 (Sigma Aldrich, P9416-50ML), 0.01% BSA (Miltenyi, 130-091-376), 1 mM DTT (Thermo Scientific), 10% EZ Lysis Buffer (Sigma Aldrich), and 0.2–1 U/µL Protector RNase inhibitor (Roche)) and incubated for 5 min on ice, then filtered using a 70 µm strainer (Martelotto et al.). This step removed undigested and fatty tissues. The nuclei were then centrifuged at 500 × *g* for 5 min at 4 °C. To clean the nuclei suspension from debris and cell membranes, Gradient Solution G30 was added, and the mixture was centrifuged at 3200 × *g* for 20 min at 4 °C (Martelotto et al.). The nuclei were washed in 1 mL of ice-cold Wash and Resuspension Buffer (WRB) (10 mM Tris-HCl at pH 7.5, 10 mM NaCl, 3 mM MgCl2, 1% BSA, 1 mM DTT (Thermo Scientific), and 0.2–1 U/µL Protector RNase Inhibitor (Roche)) before being centrifuged at 500 g for 5 min at 4 °C. The nuclei were resuspended in nuclei buffer (supplemented with DTT and 0.2–1 U/µL Protector RNase Inhibitor) and passed through a 40 µm Flowmi® Strainer^79^. The nuclei were then counted using a hemocytometer or an automated cell counter.

#### 10X genomics multiome library preparation

Nuclei were diluted to a final concentration that enabled a recovery of 10,000 nuclei. The Chromium Next GEM single Cell Multiome ATAC+ Gene Expression reagent (PN-1000283) was used. The nuclei were tagmented using ATAC buffer B and ATAC enzyme B and incubated at 37 °C for 60 min. Barcoding master mix was then added to the tagmented nuclei. We used Chip J and Multiome reagents to run 10X Chromium to generate GEMs (following the 10X User Guide CG000338 (CG000338_ChromiumNextGEM_Multiome_ATAC_GEX_User_Guide_RevF)). GEMs were cleaned up, and libraries were generated according to the 10X User Guide CG000338 manual.

#### Sequencing

Gene expression libraries were sequenced on the NextSeq 550 and NovaSeq 6000 (paired-end 150 bp reads, targeting 30,000–40,000 reads per cell). ATAC libraries were sequenced on the NextSeq 550 and NovaSeq 6000 (paired-end 150 bp reads, targeting 25,000 reads per cell).

### Single nuclei 5’ gene expression protocol

Nuclei were extracted (as above) and intact nuclei were sorted and then resuspended in PBS with 0.04% BSA with 0.2-1 U/µL Protector RNase inhibitor (Roche). The 10X Chromium 5’ Gene Expression Reagent Kit (CG000331) and Chip K were used. Gene expression libraries were sequenced on the NextSeq 550 and NovaSeq 6000 (paired-end 150 bp reads, targeting 30,000–40,000 reads per cell).

### SnPATHO-seq protocol^80^

#### Nuclei preparation

Each tissue was cut into two sections, 25 µm thick, in a 1.5 mL Eppendorf tube, then submerged in xylene for a total of 3 washes (10 min each). Rehydration was performed with sequential ethanol washes (100%, 70%, 50%, 30%) for 1 minute each, followed by 2 washes with 100% ethanol and 1 wash each with 70%, 50%, and 30% ethanol. The tissues were then digested in a digestion mix (1 mL of RPMI 1640 supplemented with 0.25–1 mg/mL Liberase TM, 0.25–1 mg/mL Collagenase D, 0.25–1 mg/mL Hyaluronidase, and 1 U/µL RNase Inhibitor) for 45–60 min at 37 °C^80^. After digestion, 400 µL of EZ Lysis Buffer was added to the sample, mixed by inversion, and centrifuged for 5 min at 850 × *g* at 4 °C. The pellet of released nuclei and undigested tissues was resuspended in 250 µL EZ Lysis Buffer with 2% BSA and 1 U/µL RNase Inhibitor, and the sample was homogenized using a Douncer 10–20 times. After homogenization, 750 µL of EZ Lysis Buffer with 2% BSA and 1 U/µL RNase Inhibitor was added, followed by 10 pipette mixes. The samples were incubated on ice for 10 min and then passed through a 70 µm PluriStrainer filter. The flow-through was centrifuged at 850 × *g* at 4 °C for 5 min. The nuclei were washed with 800 µL of EZ Lysis Buffer supplemented with 2% BSA and 1 U/µL RNase Inhibitor, then washed and resuspended in 0.5X PBS with 0.02% BSA^80^. The number of nuclei was counted using a Luna-FL dual fluorescence cell counter.

#### 10X FLEX and library preparation

The fixed nuclei were mixed with probes and hybridized overnight at 42 °C, then washed with Post Hyb washes using the Chromium Fixed RNA Profiling Reagent Kit. GEM generation was then performed using 10X Chromium. We used the Fixed RNA Profiling Reagent Kits (User Guide CG000527 Rev D) and Chip Q. The GEMs were cleaned, and libraries were generated following User Guide CG000527.

#### Sequencing

The libraries were generated and sequenced on the NextSeq 2000, paired end 28–90 bp using the P4 100 cycles kit, targeting 30,000 reads per cell.

### Whole-genome sequencing protocol

Frozen sections from the same block used for single-nuclei preparation were used. DNA was extracted with the QIAamp Fast DNA Tissue Kit (Cat#51404) from QIAGEN.150-300 ng of DNA was fragmented to ~200 bp using a focal acoustic device (Covaris S2, Sage Sciences). Libraries were prepared with NEB-Next Ultra II Kit (NEB) according to the manufacturer’s instructions. Indexed libraries were sequenced on an Illumina NovaSeq 6000 (Illumina) to generate on average, 300 million paired-end 150 bp reads per sample, if samples were sequenced by the MGC. Part of samples were sequenced by AGRF included all tumours and germline of CA90, CA83 LN 47, CA83 liver 49, CA83 LN 12, all tumours and germline of CA58, CA27 dura 14 with NovaSeq 6000 (Illumina) to generate paired-end 150 bp reads per sample. Whole-genome sequencing data from CA83 germline, CA83 lung 55, CA83 fat 19, CA76 germline, CA43 germline, all tumours and germline of CA35, CA34 germline plus two livers, CA27 germline were previous published^13^ and used in this study.

### Whole genome DNA sequencing pipeline and analysis

FASTQ quality control reports were generated for each germline and tumour sample using FASTQC v0.11.8^81^. If the FASTQC adapter content module returned a ‘fail’ for any FASTQ of a sample, then adapter trimming was performed for all FASTQs of this sample using Cutadapt v4.1^82^(command line options: -a AGATCGGAAGAGCACACGTCTGAACTCCAGTCA -A AGATCGGAAGAGCGTCGTGTAGGGAAAGAGTGT -m 25 -j 0). Reads were then aligned to the unpatched GRCh38 reference genome (accession number GCA_000001405.15) using BWA-MEM v0.7.17^83^ with post-processing for ALT-aware alignment. BAM files of the same sample were then merged, sorted, and had their PCR duplicates marked using Picard tools v2.17.3^84^.

Somatic SNVs and indels were called jointly from the germline and all tumours of the same patient using both Strelka2Pass v0.2 and FreeBayesSomatic v0.1.1^85^. For Strelka2Pass, the strelka.ini configuration file was modified to set tumour-in-normal contamination tolerance to 0 for both SNVs and indels. To remove non-passing variants which had been incorrectly retained by FreeBayesSomatic at multi-allelic loci, called variants were refiltered to those passing the default tumour and normal likelihood of difference thresholds using a custom R script. Additionally, called variants from FreeBayesSomatic with QUAL less than or equal to 30 were filtered out and the SOMATIC key in the INFO/FILTER VCF field was changed to PASS using BCFtools v1.9^86^. For both Strelka2Pass and FreeBayesSomatic, joint VCFs were split into per-sample VCFs using BCFtools v1.9 with called variants retained in the per-sample VCF only if their allele count was greater than one in that sample. For downstream analyses, only the intersection of each sample’s filtered Strelka2Pass and FreeBayesSomatic variants was retained, as obtained by the isec command in BCFtools v1.9. Per-sample SNVs and indels were then filtered to only those with a PASS flag using BCFTools v1.9, then merged into a single VCF file using GATK v3.6^87^.

SNVs and indels were annotated using VEP v98^88^ with the CADD v1.5^89^, Condel^90^, and DBNSFP v4.0a^91^ plugins and custom annotation with population allele frequency information from gnomAD v3.0^92^.

Somatic structural variants were called jointly from the germline and all tumours of the same patient using GRIDSS v2.13.2^93^ and the structural variant blacklist (sv_blacklist.bed) from 10X Genomics, then filtered using the gridss_somatic_filter R script (https://github.com/PapenfussLab/gridss/blob/master/scripts/gridss_somatic_filter) with the single-end and breakpoint panel of normal files from Hartwig Medical Foundation’s DNA pipeline v5.32^94^. Joint structural VCFs were split into per-sample VCFs using BCFtools v1.9 with called structural variants retained in the per-sample VCF only if their sample allele frequency was greater than 0.05 in that sample.

Somatic copy number variants were called for each individual tumour-normal pair using the PURPLE pipeline^95^ consisting of AMBER v3.5, COBALT v1.11, and PURPLE v3.1 with per-sample SNV/indel and structural variant VCFs as input.

CHORD^96^, HRDetect^97^, and COSMIC SBS v2 and v3.2, DBS v3.2, and Indel v3.2^98^ (available at https://cancer.sanger.ac.uk/signatures/downloads/) signature outputs were obtained via the tool wrappers implemented in the sigrap v0.1.0 R package^99^. GRIDSS structural variants were made consistent with the format expected by CHORD and HRDetect using a custom R script with the following steps. For CHORD, variants were loaded into R using the *variantsFromVcf* in the *mutSigExtractor* (v1.27) library [20], renamed to remove the ‘chr’ string from chromosome names, subsetted to only paired variants, classified using *getContextsSv* function in the *mutSigExtractor* (v1.27)^100^ library, then filtered for 0 or NA length variants. For HRDetect, variants were loaded into R using the *breakpointgr2bedpe* function in the *StructuralVariantAnnotation* (v1.10.1) library^101^, subsetted to the same variants as CHORD, then modified to add ‘sample’ and ‘sv_type’ columns, the latter taken from the CHORD input with the following value changes: ‘DEL’ -> ‘deletion’, ‘INS’ -> ‘insertion’, ‘INV’ -> ‘inversion’, ‘DUP’ -> ‘tandem_duplication’, and ‘TRA’ -> ‘translocation’.

Variants on Y chromosome and with an allele frequency in the global population less than 0.1 were excluded. Gene fusions were identified from the GRIDSS output based on genomics coordinates and gene annotations, and only fusion between two different gene bodies were considered. Genes were annotated on the copy-number segments based on hg38 gene coordinates. In cases where genes spanned multiple segments with difference copy number, the gene was assigned the copy-number of the segment that contained the largest section of the gene body. Such variants were used to build the following phylogenetic tree, while mutations with inferred impact from moderate to high by VEP were shown in oncoplot Fig. 3A.

Unweighted Pair Group Method with Arithmetic Mean (UPGMA) clustering method was used to build phylogenetic tree based on SNVs. The distance matrix was build based on alterations on single position and we kept only variants that have a meaningful functional impact (not MODIFIER), including HIGH, MODERATE and LOW impact variants, and are either rare in the population (frequency <10%) or have no known population frequency.

### snRNA data preprocessing

Initial pre-processing was performed using Cell Ranger v5.0.0 with the *cellranger count* command (command line options: --include-introns --chemistry=fiveprime) for the 5’ snRNA-seq experiments (CA83 and CA90 non-primary tumours) and Cell Ranger ARC v2.0.0 with the *cellranger-arc count* command for the multiome 3’ snRNA-seq + snATAC-seq experiments (all CA27, CA34, CA35, CA43, CA46, CA58, and CA76 tumours). For the snRNA-seq FLEX experiments (CA0090 primary tumours, v1.0.1 probe set), Cell Ranger v7.1.0 with the *cellranger multi* was used. All experiments were aligned to the GRCh38-2020-A reference genome from 10X Genomics. All subsequent analyses were conducted in the R (v4.1.0) programming language.

To reduce artefactual variation stemming from incomplete nuclear isolation, a custom analysis approach was taken for non-FLEX experiments. Using a custom R script, snRNA-seq UMI counts were classified into intronic and exonic counts based on the molecule_info hdf5 file generated by Cell Ranger to obtain separate intronic and exonic gene count matrices. Subsequent cell filtering, unsupervised clustering and differential expression steps were performed based only on the intronic gene count matrix.

First pass filtering aimed to remove droplets containing only ambient RNA. For the non-FLEX experiments, droplets containing only ambient RNA were removed using EmptyDrops^102^ with an FDR cutoff of 0.001. For the FLEX experiments, only droplets with more than 300 total RNA UMI counts in the raw gene count matrix from Cell Ranger were retained.

Normalisation, PCA dimensionality reduction, unsupervised Louvain clustering, and UMAP visualisation were performed using *Seurat* v4.1.0^103^. For the non-FLEX experiments, the 2000 most highly variable genes were selected based on their residuals from the expected mean-variance relationship using the *modelGeneVarByPoisson* function in the scran v1.26.2^104^. For the FLEX experiments, all genes were used. Additionally, for the non-FLEX experiments, the first 20 PCs were used, whereas the first 30 PCs were used for the FLEX experiments. This pre-processing was repeated after each filtering pass.

Second pass filtering aimed to remove doublets and non-nuclear debris. Doublets were removed using scDblFinder v1.9.2^105^. For the non-FLEX experiments, non-nuclear debris was removed by identifying clusters with high mitochondrial gene expression. For the FLEX experiments, non-nuclear debris was removed by identifying clusters with high expression of genes known to localise to the cytoplasm and low expression of genes known to localise to the nucleus, based on genes curated from an mRNA localisation experiment^106^.

Third pass filtering aimed to separate tumour cells from non-tumour cells. Cells were annotated using *SingleR* v1.8.1 and *celldex* v1.4.0^107^ using the *HumanPrimaryCellAtlasData*^108^ and *BlueprintEncodeData*^109,110^ reference datasets available in *celldex*. Clusters of cells annotated as epithelial cells were retained as likely tumour cells. The tumour cell identities were later verified using InferCNV, although the InferCNV plots were not included.

Normal cell clusters were primarily annotated when over 50% of cluster cells obtaining non-epithelial or non-neutron annotations and without tumour biomarkers (*AR* for prostate cancer). The division between normal and tumour cells was further validated using InferCNV and the other CNA inference tool (see below). Cycling scores calculated through G2M and S stage markers expression were provided through *CellCycleScoring()* function of Seurat to predict cells cycling stages. In summary, there were 527–13,929 cells in samples, 5551 on average and 188,744 cells in total. The tumour cells proportion across samples ranged from 41% to 100%.

### Gene set scoring

Gene sets were scored using AddModuleScore() in Seurat (v4.4.0). We included gene sets related to the following: cell morphology (e.g. basal, luminal, squamous and stemness); fundamental biological process (e.g. cell division, cell differentiation, apoptosis, quiescence, senescence and inflammation response), cancer hallmark pathways (e.g. hypoxia, stress, EMT, MYC signalling), DNA damage and repair^50,74,111–115^. Additionally, we included gene sets related to prostate cancer subtypes, such as genes in the AR signalling pathway, NE tumour markers, amphicrine signatures, gastric-intestinal subtypes, and WNT-related and stem-cell like subtypes^9,116,117^. Given the diversity of genes in each signature depending on source, we curated signatures to include genes recurrent in over 50% of the available signatures with similar functions (Supplementary Data 2).

### Gene set scores linear model tests

To determine whether sites of metastasis were associated with signature expression levels, linear models were fitted for each signature’s mean z-score. A base model regressing signature score on patient identity ( ~ patient) was compared to an extended model adding metastatic lesion ( ~ patient + organ) using likelihood ratio tests (ANOVA F-test) to determine significance (Supplementary Data 3).

### Integration of snRNA expression and scATACseq of samples within patients and across the patients

To integrate multiple snRNAseq samples, we used a two-tier approach. Data integration was first completed through simspec (v0.0.0.9)^118^ based on intrinsic reference on preliminary Louvain clusters to remove technical variance between sample experiments, which resulted in secondary unsupervised clustering results according to similarity between preliminary clusters. Since simspec does not correct the expression values for batch effects, batch effects were removed with RUV-III-NB^119^ using the preliminary simspec clusters as pseudo-replicates. This resulted in a normalized matrix without unwanted variants due to batches. Single-cell housekeeping genes were used as background controls^120^. Tumour only cells integration used tumour cells identified in previous sections (see above), the sample procedure was conducted. Later, UAMP was built with standard Seurat workflow with 20 principle components of PCA.

The integration of snATACseq samples followed a multi-step approach. The same tumour cells used for our snRNAseq analyses were extracted, and peaks were called with MAC3 (v.3.0.3) through Signac (v.1.16.0). Peak matrices were re-built using shard peaks and merged across all samples. The batch effect was again removed with RUV-III-NB^119^. Samples from the same patients were used as pseudo-replicates in the RUV-III. Stably open peaks across cell types and found in shared peaks were used as background control^47^. Single value decomposition (SVD) was performed using Signac with open peaks excluding background peaks. UMAPs were built using 2 to 30 components from SVD. Gene activity was inferred from the open peaks using *GeneActivity* through Signac (v.1.16.0).

### Normal cells annotation

The integrated dataset was used for normal cell annotation. SingleR (v1.8.1) was used to preliminarily annotate cells. Normal cells often clustered together, resulting in clear annotations of cells. A subset of normal cells clustered with tumour cells, but these were considered contaminated with tumour ambient RNA and removed. Within selected normal cells, cells showing high expression of markers of two major category cell types (e.g. fibroblasts and T cells) were also removed due to the possibility of being doublets or contaminated cells. In total, there are 13430 normal cells included for further microenvironment analysis. For accurate clustering to better subtype cells, Harmony (v0.1.0)^121^ was used to generate integrated clusters and overcome batch effects between experiments. Accurate normal cell annotations were achieved by combining preliminary annotation with SingleR and cross validation with expression of cell type specific markers from previous studies (Supplementary Note 1). This resulted in several major categories of cells identified, including fibroblast-like cells, endothelial cells, epithelial cells, T cells, B cells, and macrophages. A second layer of subtyping was performed for each major category. The annotations were given to Louvain clustering, obtained a resolution set at 1 in Seurat.

### Normal cell composition analysis

To determine whether the cell type distribution varied across patients and metastatic lesions, we used linear modelling and ANOVA embedded limma (v 3.66.0). The cell type proportion was calculated by dividing cell counts by the total normal cells number in samples. The proportions were logit transformed and fitted into design ( ~ patient + sties). topTable() was used to perform ANOVA across lesions with results recorded in Supplementary Data 7. All p-values were collectively BH-corrected across all cell types and sites. Before using this model, multiple nested models with patient, sites, and pathology either as random or fixed effect were tested while ( ~ patient +sites) was the model fitted the best. To assess patient-level differences in the abundance of specific cell types (e.g. neurons in CA90), the transformed proportions were fitted to a model with patient as the only variable ( ~ 0 + patient). Then, a contrast matrix comparing CA90 against all the other patients was fitted to the model, followed by ANOVA test through topTable() in limma.

### Association between cell type distribution and signature expression

To investigate the association between normal cell proportion and signature expression, we used a one-sided Jonckheere-Terpstra test with a 0.05 *p* value cutoff.

For each cell type, only samples had the number of the cell type greater than 10 counted in the tests and with samples tiled into 5 levels of genes/signatures expression, only tests with more than 3 samples in any expression level groups were done.

### Cell-cell communication

The cell-cell communication between tumours and normal tumour cells for individual samples was inferred using CellChat (v.2.2.0). Results were described as either specific ligand-receptor pairs or the pathways they involved. We focused our analysis on the interaction from non-tumour cells to tumour cells. Cell-cell interactions were considered only if adjusted *p* values < 0.05, probability >0.05, and there were more than 10 source (microenvironment) cells in the samples. Results were only included if they occurred in at least 3 samples and 2 patients. Enriched tumour receptors were considered regardless of ligand-receptor pairs; thus enriched receptors across ligand-receptor pairs are shown in Fig. 4d.

### ATAClone

We developed a method to derived copy-number information from single-nuclei ATACseq data^30^. To obtain a set of regions with maximum DNA copy number signal and minimum signal corresponding to transcriptional differences or ATAC-seq technical effects, a reference set of peak regions was created based on the Altius atlas of DNase I hypersensitive sites^122^. Only peaks flagged as present in all 15 organ systems contained in the atlas were retained, for a total of 76,951 peaks, henceforth referred to as ‘stable peaks.’

ATAC-seq fragments were de-duplicated based on their start and end position, filtered to only those intersecting stable peaks based on the ‘paired insertion count’ method^123^, then assigned to non-overlapping 10MB genomic bins.

Droplets initially included only those retained after the second pass filtering step of the snRNA-seq pre-processing workflow but were further filtered to exclude droplets with fewer than 300 total stable peak fragments or with a fraction of fragments in stable peaks less than 0.05.

Bin counts were normalised using the *acosh_transform* function in *transformGamPoi* v1.4.0^124^ with the *overdispersion* parameter set to 0.025 and the *size_factors* parameter set to FALSE. Dimensionality reduction was performed on the normalised values using the *prcomp* function in base R, with the first principal component, corresponding to total stable peak counts, discarded, and the number of leading principal components to keep estimated from the data.

A KNN graph was built from the PCA embeddings using the *buildKNNGraph* in *scran* v1.26.2 with k = 11. Graph-based clustering was performed using the Leiden algorithm^125^ as implemented in *igraph* v2.1.0.9008^126^. As a heuristic, the CPM resolution parameter was set to (k – 1) / (vcount(knn.graph) − 1). UMAP visualisations were computed using the *umap* v0.2.10.0 package^127^.

For DNA copy number estimation, a reference cluster consisting of normal cells was identified based on the SingleR annotation obtained during the snRNA-seq pre-processing. DNA copy number was then estimated for each bin at the per-cluster level by summing across all members of a cluster, dividing by the total bin counts in each cluster to obtain a proportion, dividing each cluster’s bin proportions by the bin proportions of the reference cluster, then multiplying by 2 for autosomes (all samples are male).

All ATAClone analysis was conducted in the R (v4.2.0) programming language. The code used to generate the ATAClone results is available at https://github.com/TrigosTeam/Recurrent-Tumour-Single-Cell/tree/ATAClone.

### Archetype analysis

To identify populations of cells across our cohort of tumour cells, we used archetype analysis. Here, a Pareto task inference method was used to infer biological tasks from snRNAseq. Principle Convex Hull Analysis algorithm was used to describe snRNAseq data as a polytopes using the ParetoTI R package (v0.1.13)^128^. The features enriched at the vertices (archetypes) of polytopes correspond to more extreme phenotypes of the populations. The Elbow method was applied to determine the number of archetypes present in each sample. Here, the inflexion point on the plots of explained variance versus number of archetypes was selected. Across the samples, 5-6 archetypes were determined in each sample through 200 times bootstrap with resampling 70% of cells. The top 10% cells nearest to the archetype position were assigned to the archetypes and used to generate enriched archetype gene sets. Differentially expressed genes (DEGs) in the cells closest to the archetypes were identified using the FindAllMarker() function with MAST tests. The identified DEGs for each archetype and sample were clustered using hierarchical clustering with *k* = 9. Genes recapitulated in over 25% of archetype groups were selected. Only gene modules composed of more the 10 genes while exclusively represented only one module were retained. Small modules (*n* < 10) which recapitulated similar signatures to other modules or which were only found in sample specific subpopulations were removed.

Biological pathways associated with the gene modules were investigated with gene set enrichment analysis using clusterProfiler (v4.4.2) with HALLMARK, REACTOME, KEGG gene sets from Molecular Signatures Database^129^. Cells were considered to belong to an archetype module if they were in the top 50% expression calculated with AddModuleScore() of the signatures in the entire dataset. Cells assigned to multiple modules using this procedure were considered as transitioning between states.

### Identification of differentially expressed genes and gene set enrichment analysis

Differential expressed genes and log-fold changes were calculated using FindMarker or FindAllMarker functions in Seurat using the MAST method and the *p* values were adjusted using Bonferroni correction.

Enrichment of gene sets was performed using the hallmark gene sets, oncogenic signature gene sets and ontology gene sets from Molecular Signatures Database (MSigDB)^129^ using the Gene Set Enrichment Analysis (GSEA) function in clusterProfiler(v4.2.2) in R.

### Identification of differentially open peaks and significant binding motifs

Differentially open peaks between tumour populations were identified using the FindAllMarker function in Seurat with the Wilcoxon rank test. Enriched motifs potentially binding on the differentially open peaks were inferred using the FindMotif function from Signac at *p* values cutoff at 0.05.

### Copy number subclone effect on archetype signature variance

We aimed to determine whether sample subclones were linked to (i.e. contributed to the variation of) archetype signature expressions beyond what could be explained by inter-patient and intra-patient heterogeneity. First, we calculated scored for the levels of expression of the archetype signatures at the single-cell level using AddModuleScore(). Next, we built nested random-effects models which were applied to the signature scores. The three nested linear mixed models of increasing complexity were compared using likelihood ratio tests (ANOVA) with REML = FALSE for model comparison.

The null model had patient identities and sample identities as random intercepts, which was compared to the alternative model adding subclone nested within samples as an extra random effect. A second alternative model added metastatic sites as another random intercepts. Through ANOVA F-test across three models, the alternative model with patient, sample, and subclone as the random intercepts showed best fitted (*p*-value < 2.2 × 10^−16^). Then, the proportion of variance attributable to subclone-level random effect was extracted from the variance components of the subclone model. All likelihood ratio test p-values were BH-corrected.

### Statistics and reproducibility

Sample selection was based on the sample quality of available rapid autopsies collected through the CASCADE program based on the criteria stated in the “*Sample selection”* section. Additional sample information is recoded in Supplementary Data 1. The samples were solely from male patients diagnosed with mCRPC as it is a sex-limited disease.

All analysis and figures were made in R (4.2.0) except for Fig. 1A and Fig. 1D which were made with Biorender (Created in BioRender, Weng. S. (2025) https://BioRender.com/o10z063). *P* values of 0 or <2.2×10^−16^ represent values below the minimum representable number in base R. Adjusted *p* values were computed using Bonferroni correction or Benjamini-Hochberg methods as default if not specified for multiple testing. Later additional analysis was conducted in R (4.5.0), including all analysis for ATACseq and CellChat. No data were excluded from the analyses after standard cell quality control.

### Reporting summary

Further information on research design is available in the Nature Portfolio Reporting Summary linked to this article.

## Supplementary information

Supplementary Information

Description of Additional Supplementary Information

Supplementary Dataset 1-12

Reporting Summary

Transparent Peer Review file

## Data Availability

The whole-genome DNA sequencing data and single-nuclei RNA-seq and ATAC-seq data generated in this study are available from the European Genome-phenome Archive under accession number EGAS50000001312 with no time limit. Since the data includes patient genetic information, researchers who wish to apply for access require human ethics approval. The average response time to the access request is 14 business days. Previously published whole-genome sequencing data used in this study can be found under accession number EGAS00001006598^13^. The publicly available bulk RNA-seq data shown in Fig. 5f can be found at GSE126078^50^. The publicly available data used in Supplementary Note 2 includes single cell data from GSE137829^74^, bulk RNA-seq data from E-MTAB-9930^130^ and Xenograft bulk RNA-seq data from GSE41193^131^. The remaining data are available within the Article, Supplementary Information or Source Data file (10.5281/zenodo.20019864)^132^.

## Code availability

The code to reproduce all the results of the paper can be found at: https://github.com/TrigosTeam/Recurrent-Tumour-Single-Cell. The same custom R scripts used have been deposited in Zenodo (10.5281/zenodo)^133^.
