# Supplementary material for: Recurrent intra-tumour heterogeneity is a hallmark of metastatic prostate cancer
Source: Nat Commun. 2026 Jun 19;17:7727. doi: 10.1038/s41467-026-74334-z (PMC13434311; doi:10.1038/s41467-026-74334-z)
Supplement: Supplementary file 1 [file 41467_2026_74334_MOESM1_ESM.pdf]

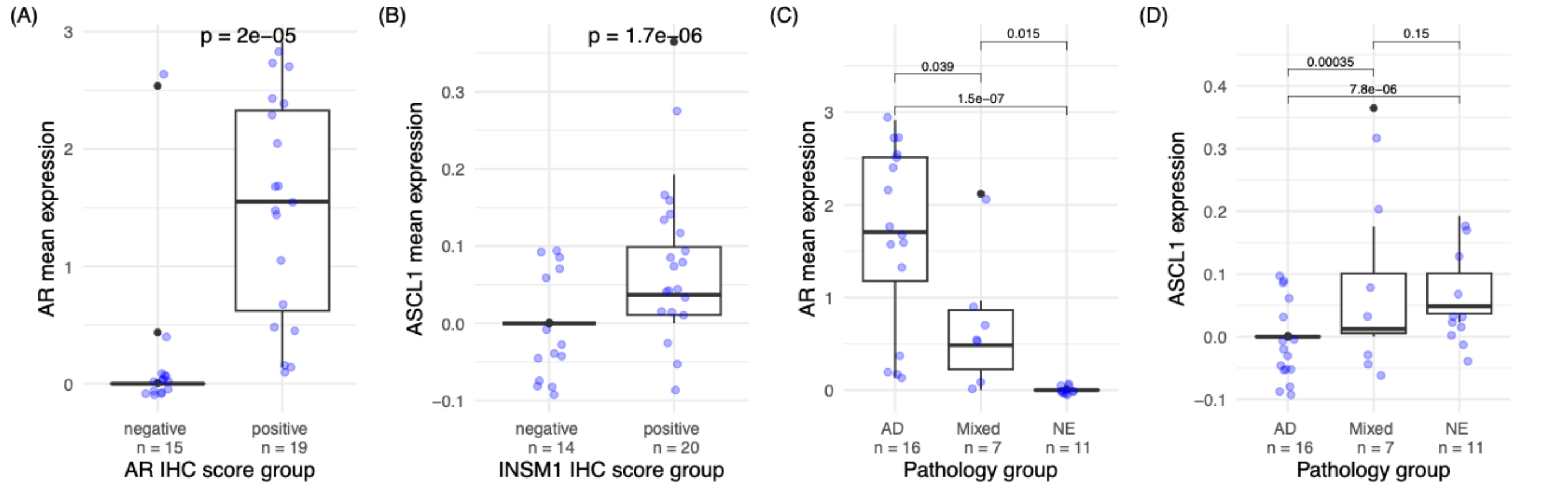

Supplementary Figure 1: AR and ASCL1 gene expression in histology and pathology groups.

(A) AR expression is significant higher in samples with positive AR IHC stain (n=19, mean = 1.51) . There is little but not no expression of AR in the samples with no AR staining (n =15, mean = 0.199).

(B) ASCL1 expression is significantly higher in samples with positive INSM1 IHC stain (n=20, mean = 0.0725) comparing to negative INSM1 group (n=14, mean = $8.16x10^{-5}$).

(C) AR expression is highest in AD tumours (n=16, mean = 1.68), followed by Mixed (n = 7, mean = 0.672), then NE tumours (n = 11, mean = 0.00113). ,

(D) ASCL1 expression is comparable between Mixed (n = 7, mean = 0.0842), and NE tumours (n = 11, mean = 0.0782) while rare in AD ones (n=16, mean = 0.000133).

The statistical test used was two-sided Wilcoxon rank test. P values were adjusted by Bonferroni method. The median is shown by the centre line of box plots. The box spans from the 25th to the 75th percentile. The whiskers extend to the most extreme values within 1.5×IQR of the box hinges. Outliers are plotted individually beyond the whiskers as black dots.

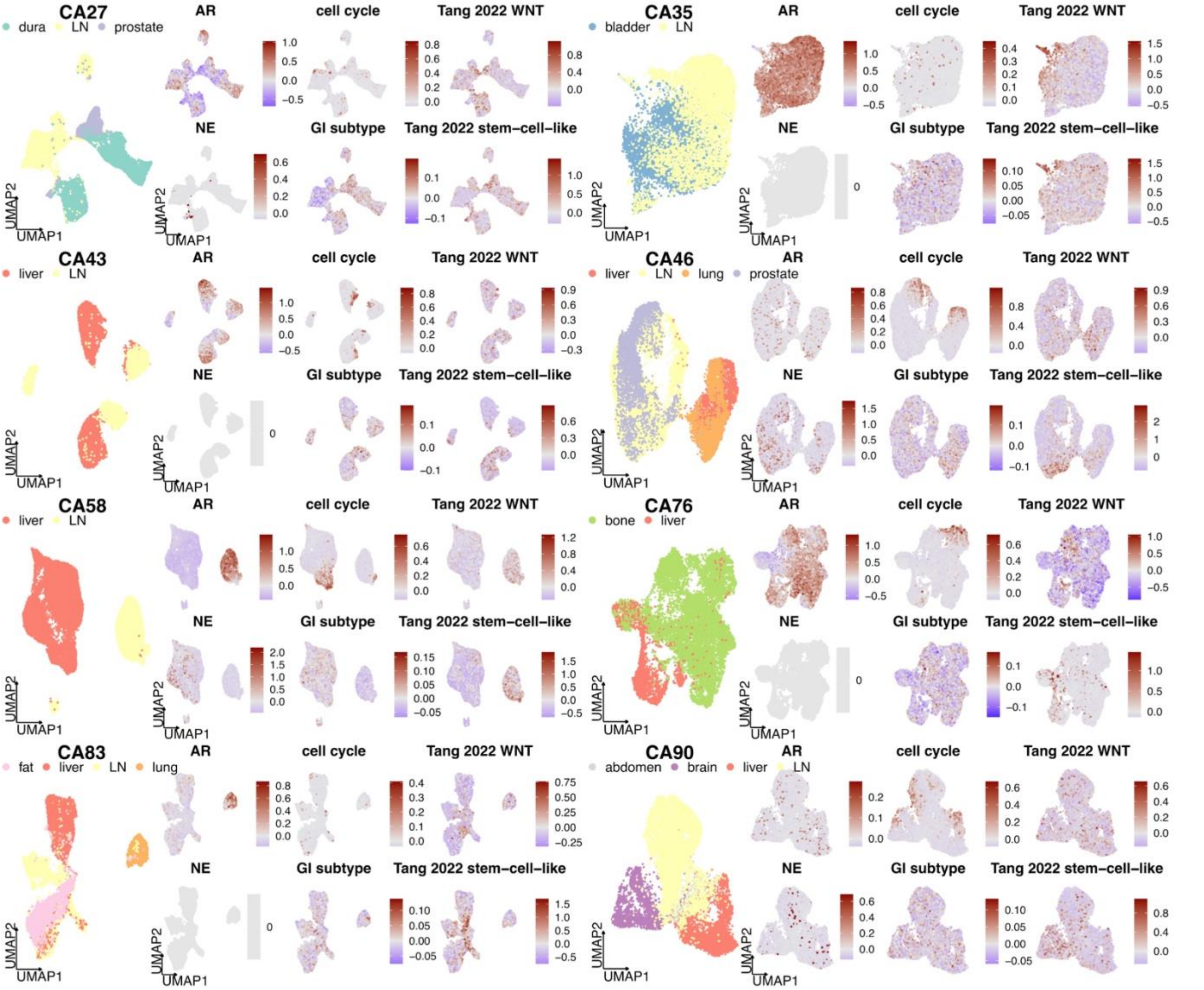

Supplementary Figure 2: Expression of AR related, NE phenotype, cell cycle, GI, WNT subtype, and stem-cell-like subtype in tumour cells across all lesions of individuals.

Signature expression in CA27 (*n* = 25,488 cells across 4 samples: dura = 12,532, LN = 9,883, prostate = 3,073), CA35 (*n* = 9,083 cells across 2 samples: bladder = 4,293, LN = 4,790), CA43 (*n* = 21,936 cells across 3 samples: liver = 14,750, LN = 7,186), CA46 (*n* = 21,491 cells across 6 samples: liver = 3,143, LN = 11,995, lung = 2,648, prostate = 3,705), CA58 (*n* = 26,151 cells across 3 samples: liver = 18,893, LN = 7,258), CA76 (*n* = 12,387 cells across 3 samples: bone = 10,416, liver = 1,971), CA83 (*n* = 16,583 cells across 5 samples: fat = 3,167, liver = 5,023, LN = 6,362, lung = 2,031), and CA90 (*n* = 13,300 cells across 5 samples: abdomen = 484, brain = 2,459, liver = 3,908, LN = 6,449) tumours, respectively. The genes belonging to each signature is presented in Supplementary Table S2. Limited inter-lesion heterogeneity was shown by the six signatures. Expression of signatures was uniform except proliferating tumour cells clustered. Importantly, the cell populations with low AR or NE signatures were not further explained by the rest signatures, showing no distinguishment between CA83 lung and other CA83 tumours in E. The NE signature has zero expression in all AD samples, little in Mixed samples and more in NE samples while interestingly, AR signature expression was found in NE tumours.

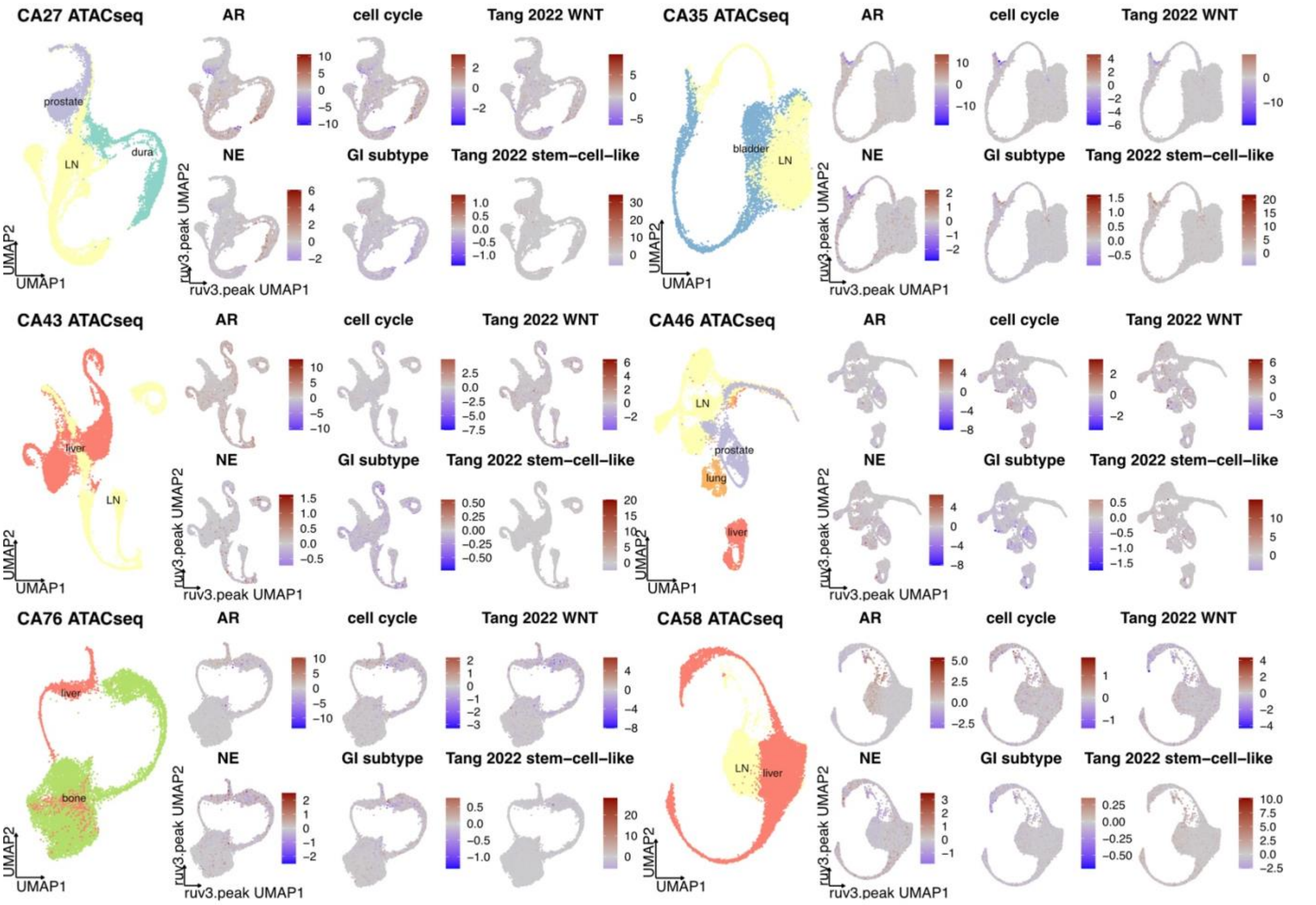

Supplementary Figure 3: UMAPs derived using snATACseq showing the levels of AR-related, NE phenotype, cell cycle, GI, WNT subtype, and stem-cell-like subtype signatures in tumour cells across all patient lesions. The signature scores were derived using the inferred gene activity from snATACseq in CA27 (n = 17,261 cells across 4 samples: dura = 4,730, LN = 9,620, prostate = 2,911), CA35 (n = 8,647 cells across 2 samples: bladder = 4,116, LN = 4,531), CA43 (n = 21,463 cells across 3 samples: liver = 14,469, LN = 6,994), CA46 (n = 19,989 cells across 6 samples: liver = 3,024, LN = 11,405, lung = 1,911, prostate = 3,649), CA58 (n = 24,442 cells across 3 samples: liver = 17,642, LN = 6,800), and CA76 (n = 11,410 cells across 3 samples: bone = 9,507, liver = 1,903) tumours, which have snATACseq available. The same signature gene list used for Supplementary Figure 2 was used. Signature levels are overall weaker to those obtained with the snRNAseq data. Compared to level of clustering and number of clusters obtained with the gene expression data, chromatin accessibility showed less heterogeneity, especially between lesions.

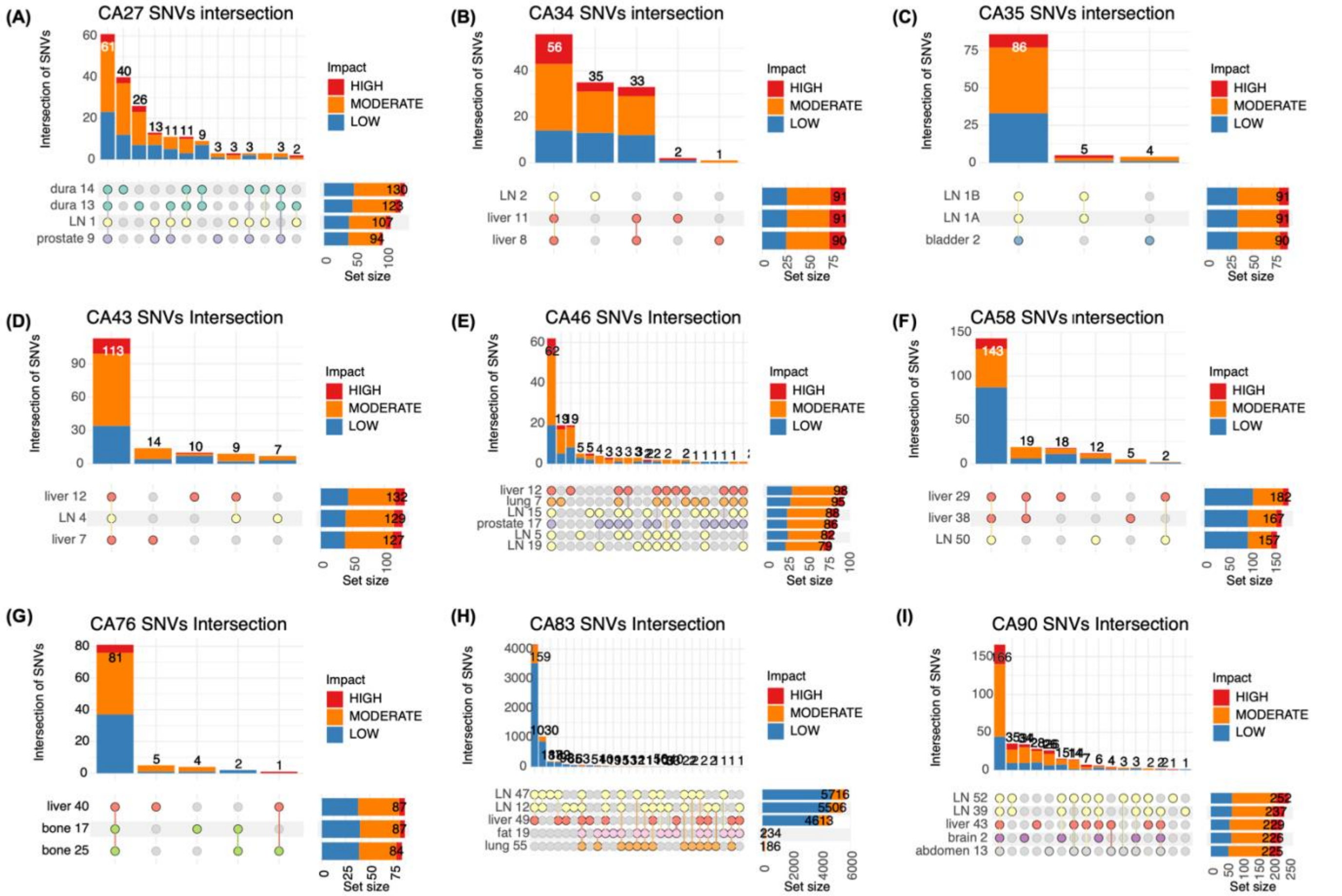

Supplementary Figure 4: UpSet plots showing the SNVs intersection between lesions of individuals.
(A-I) UpSet plots of all patients. Little genetic inter-lesion heterogeneity was observed as most SNVs were shared between all samples within patients, similar number of SNVs were called among lesions and a similar proportion of SNV impacts were identified, with the only exception being the fat and lung samples of CA83. The height of the bar represents the number SNVs overlapped between samples linked in the below panel. The colours of the bar are split by impacts annotated by VEP. Besides SNVs shared by all lesions, tumours from the same organ were more likely to share SNVs.

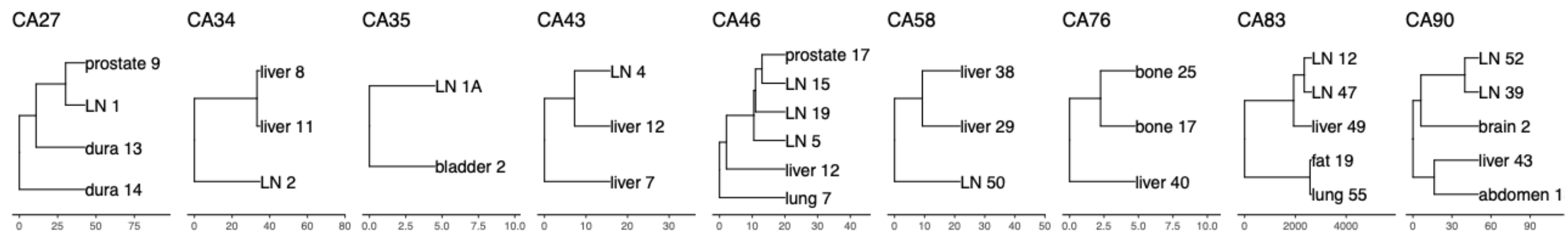

Supplementary Figure 5: Phylogenetic trees based on genetic variants for tumours within patients. Phylogenetic trees of samples within each patient built based on SNVs. Samples collected from the same organ are more closely phylogenetically related, even though the location of these sites is not likely the result of clone expansion within an organ (e.g. right and left liver lobe in CA34). Exceptions are linked to inter-tumour differences in variants in Fig, 3a where significant mutations occurred. For example, whole-genome doubling was a branching point of tumour evolution in Mixed patients CA27 and CA58, leading to distinct clones between lesions of the same organ.

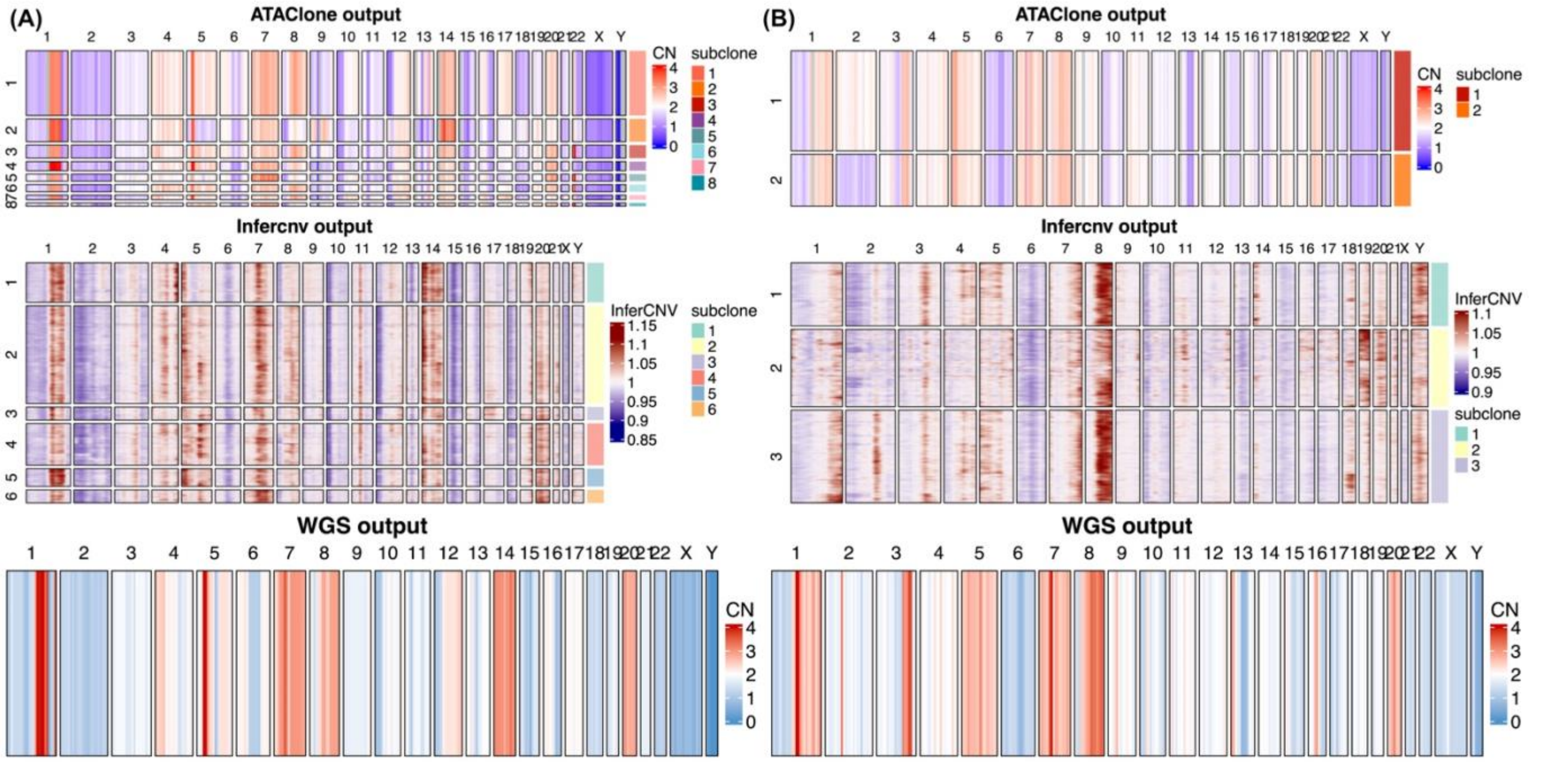

Supplementary Figure 6: Tumour CN subclones inference results from ATAClone and InferCNV compared to CN calls from WGS data.

(A) CN subclones (n = 8) inferred by ATAClone (top), InferCNV (middle) and called from the WGS pipeline (bottom) of the CA27 LN 1 sample. The sample is an example of a tumour with great clonal diversity. The relative CNV levels inferred by both tools agree with WGS results, with large amplifications on chromosomes 1, 7, 8, 14 and 20 correctly inferred. However, the CN difference between subclones is clearer by ATAClone estimation, especially for subclones with fewer cells. For example, in subclone 2 in ATAClone, which corresponds to subclone 1 in InferCNV, there loss of an amplification on chromosome 8 and 20, which can be easily observed in ATAClone but difficult in InferCNV. ATAClone estimated eight subclones, while InferCNV could only identify six.

(B) CN subclones (n = 2) inferred by ATAClone (top), InferCNV (middle) and called from the WGS pipeline (bottom) of CA76 vertebrae 25. Here, chromosome 19 is diploid according to WGS results, which ATAClone correctly inferred but an amplification was found by InferCNV in a large proportion of cells. Thus, in general, ATAClone outperforms InferCNV with less noise enabling easier subclone identification.

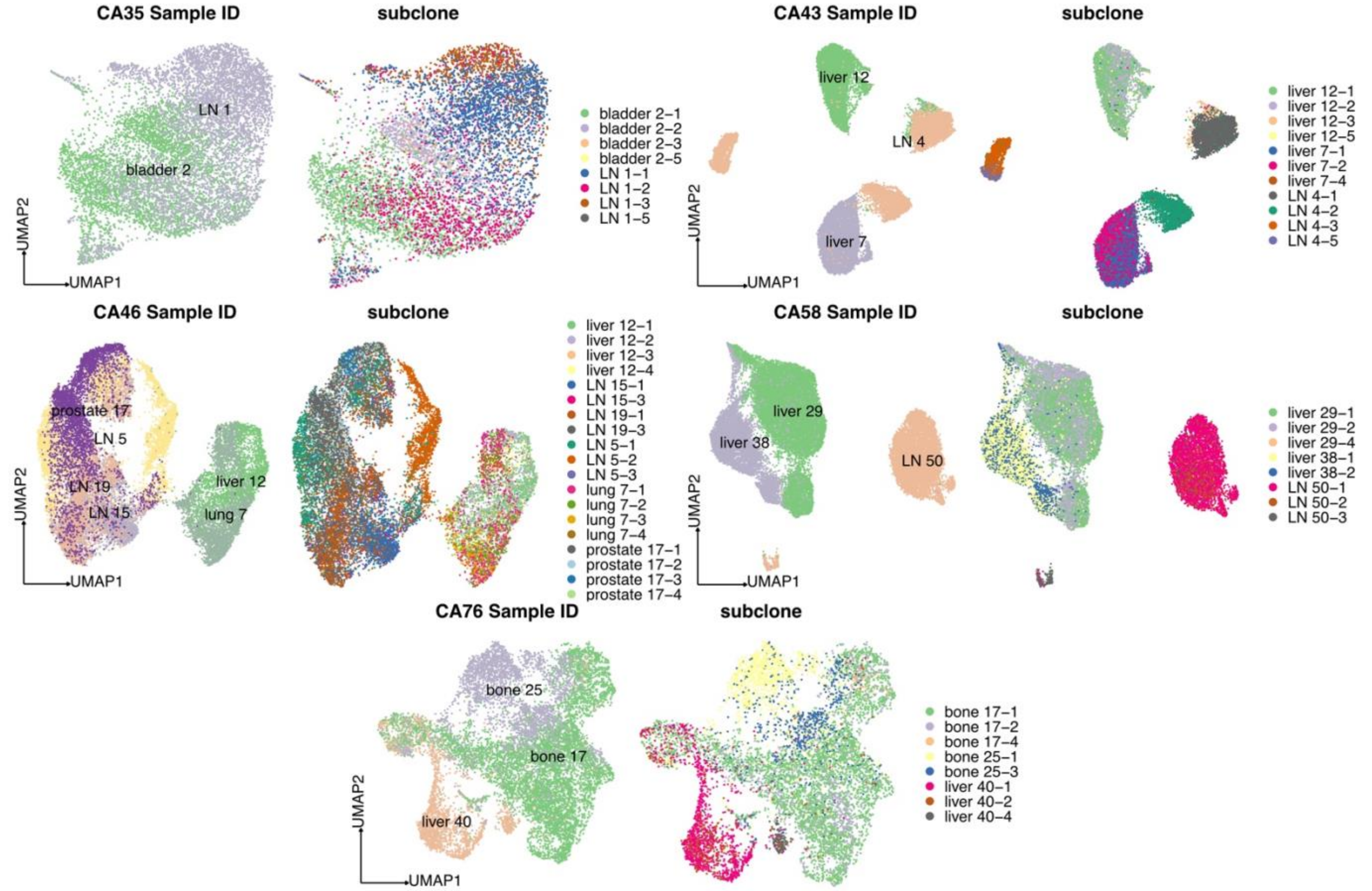

Supplementary Figure 7: CN subclones in the samples from patients of CA35, CA43, CA46, CA58 and CA76. Tumour cells integrated across patients CA35 (*n* = 7905 cells across 8 subclones: LN = 4075 [4 subclones]; bladder = 3830 [4 subclones]); CA43 (*n* = 20311 cells across 11 subclones: liver = 13683 [7 subclones]; LN = 6628 [4 subclones]); CA46 (*n* = 19902 cells across 16 subclones: liver = 2747 [3 subclones]; LN = 10081 [8 subclones]; lung = 1704 [4 subclones]; prostate = 3426 [4 subclones]); CA58 (*n* = 21218 cells across 8 subclones: liver = 15787 [5 subclones]; LN = 5431 [3 subclones]); and CA76 (*n* = 8977 cells across 8 subclones: bone = 7321 [5 subclones]; liver = 1656 [3 subclones] are shown in UMAPs built with integrated gene expression. Samples from the same patient often separated into different clusters. Clusters were linked to distinct CN subclones either within the same sample or between samples. Therefore, separation of lesions from different organs is likely tied to underlying CN differences. UMAPs shown were generated using the snRNAseq data.

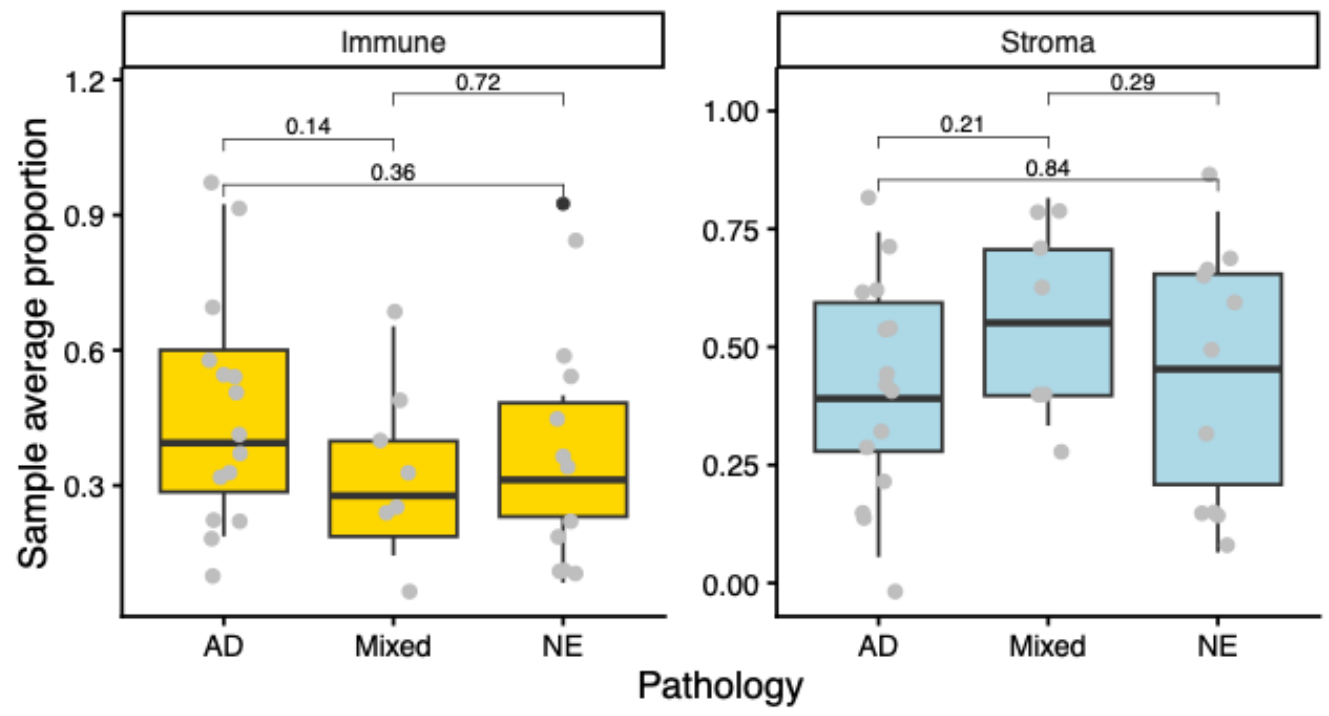

Supplementary Figure 8: Comparison of immune and stromal sample composition between pathology groups. Wilcoxon rank two-sided test between immune and stroma proportions in individual samples (n = 33 samples) across histological group. The p values were adjusted through Bonferroni method and there is no significant p values. The median is shown by the centre line of box plots. The box spans from the 25th to the 75th percentile. The whiskers extend to the most extreme values within 1.5×IQR of the box hinges. Outliers are plotted individually beyond the whiskers as black dots. Means of immune composition in AD (n = 15 samples), Mixed (n = 7 samples), and NE (n = 11 samples) are 47.4%, 32.1% and 37.1% respectively. Means of immune composition in AD (n = 15 samples), Mixed (n = 7 samples), and NE (n = 11 samples) are 41.8%, 55.8% and 44.0% respectively. CA83 fat 19 does not have any non-tumour cells.

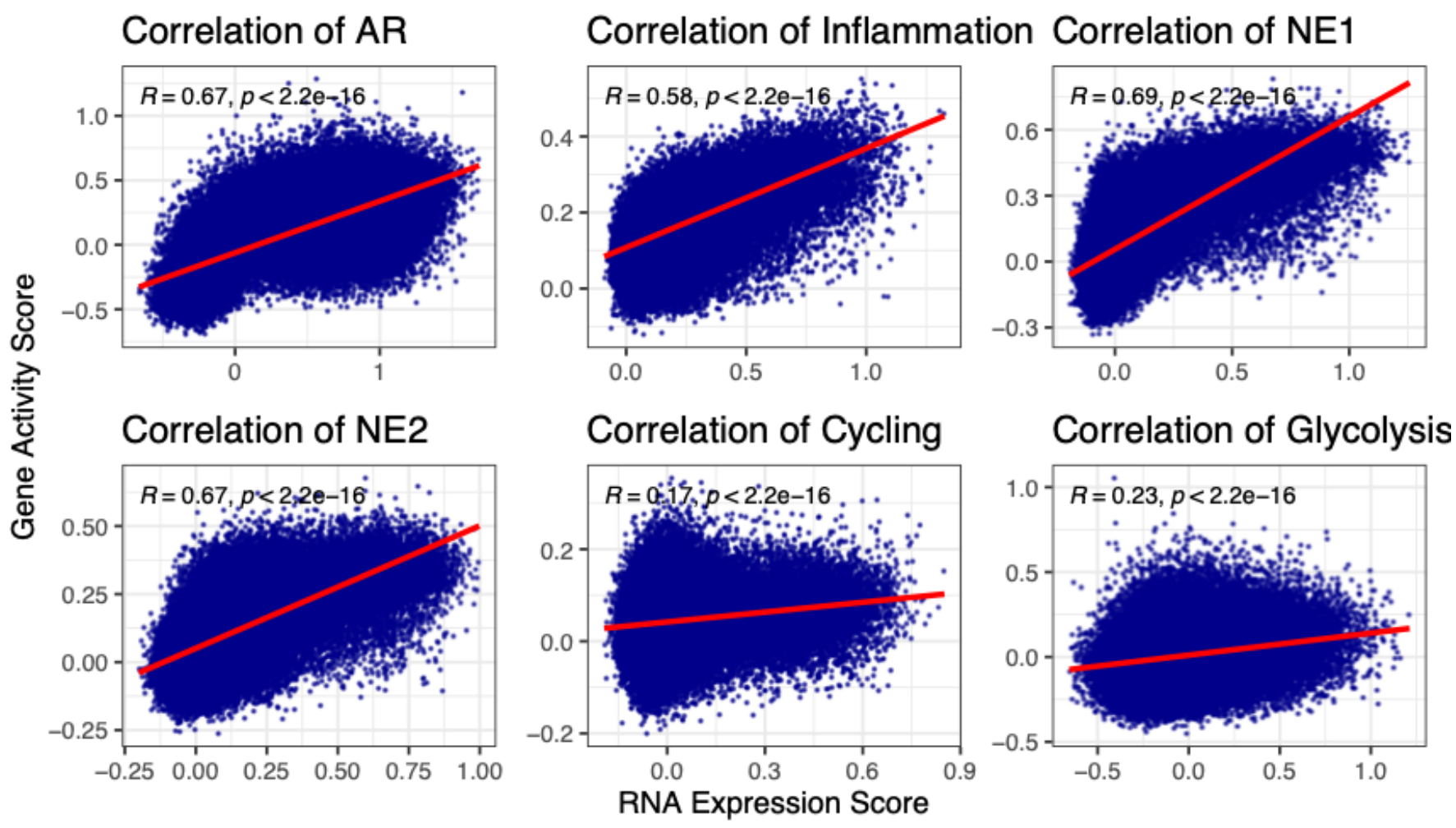

Supplementary Figure 9: Correlation between gene expression of archetype modules and gene activity inferred from snATACseq data across the cohort .

The cells available in both RNAseq and ATACseq are included (n = 120708 cells, 24 samples, 7 patients). The inferred gene activity from snATACseq data was used to calculate the signature scores. The Spearman correlation between these scores and those obtained using the snRNAseq results was above 0.5 for all modules except the Cycling and Glycolysis Modules. All adjusted p-values by Bonferroni method were significant indicated as $p < 2.2x10^{-16}$.

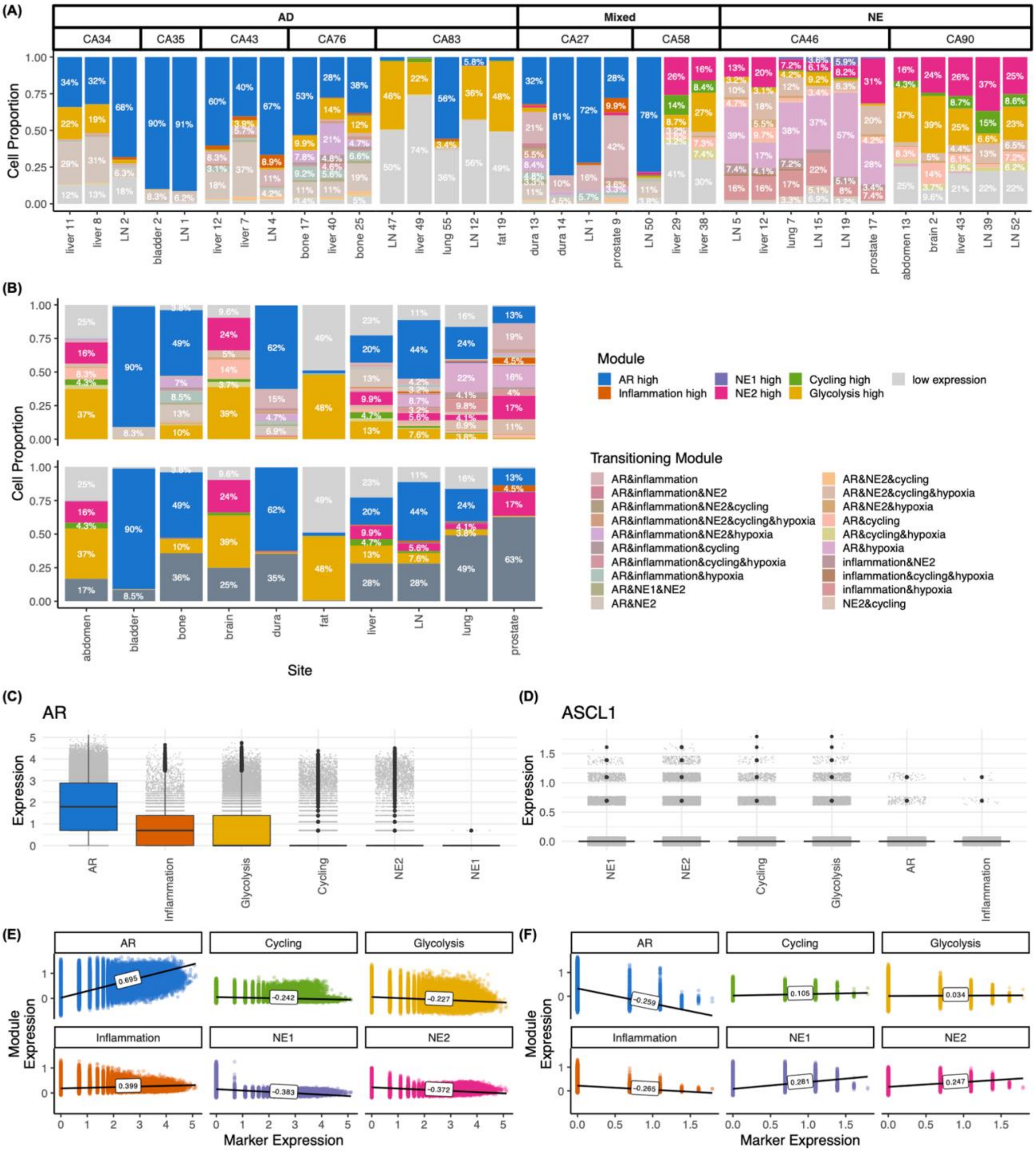

Supplementary Figure 10: Composition of archetype modules across samples (n = 34) and sites, and association with *AR* and *ASCL1* gene expression.

(A) Composition of archetype modules across samples. (B) Composition of archetype modules across organs. There was no organ specific pattern of module composition in cells with an absolute module annotation (top) or in a transition state (bottom). (C-D) Expression AR and ASCL1 in tumour cells based on their module annotation. Only tumour cells with exclusive top 50% expression of modules represents absolute archetypes. There are 57,151 AR Module cells, 4,007 Cycling Module cells, 16,721 Glycolysis Module cells, 1,753 Inflammation Module cells, 434 NE1 Module cells, and 11,498 NE2 Module cells, summing up to 91,564 tumour cells. The *AR* gene has the highest expression in cells of the AR module group, followed by those from the Inflammation and Glycolysis Modules. *ASCL1* has highest expression in cells of the NE1 Module, and lower in those of the NE2 and Cycling Module. The median is shown by the centre line of box plots. The box spans from the 25th to the 75th percentile . The whiskers extend to the most extreme values within 1.5×IQR of the box hinges. Outliers are plotted individually beyond the whiskers as black dots. (E) Spearman correlation between module signatures expression and expression of *AR* in all tumour cells (n = 164726). *AR* is most positively correlated with AR Module expression, followed by the Inflammation Module, and is negatively correlated with the rest modules. The Spearman correlation adjusted p values are less than 2.2×10^{-16}. (F) Spearman correlation between module signatures expression and expression of *ASCL1* in all tumour cells (n = 164726). NE1 is more correlated with *ASCL1* expression than NE2. The Spearman correlation adjusted p values are less than 2.2×10^{-16}.

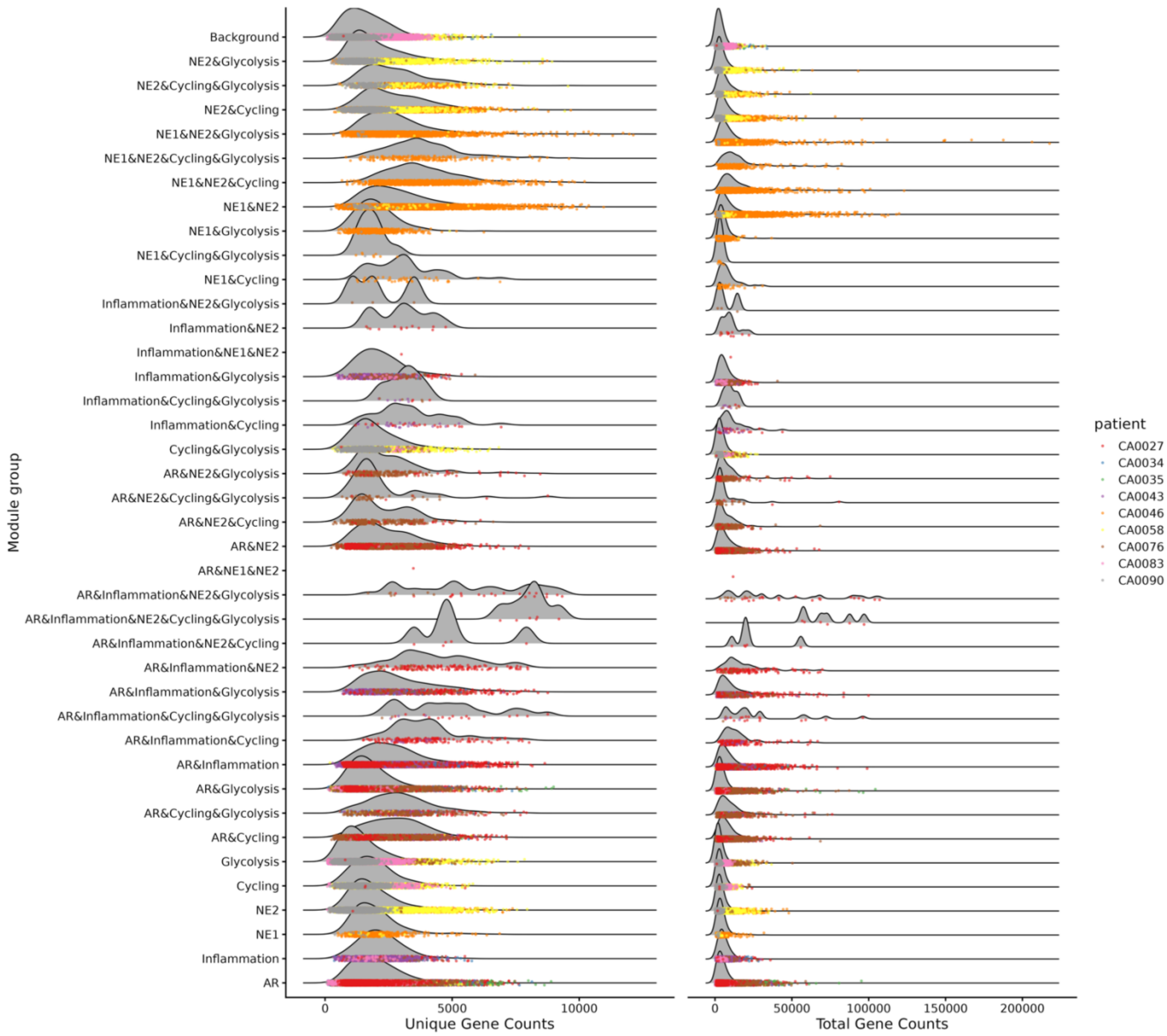

Supplementary Figure 11: Unique gene counts and total gene counts of cells with high expression of an archetype module signature and transitioning cells.

Comparing unique gene counts and total gene counts of cells classified as being of a particular archetype module (bottom) and cells with multiple signatures, i.e. transitioning cells (top 2 - 34) have similar quality metrics. The number of cells and patient origin are shown by the points beneath the ridges. Transitioning cells with outlier quality metrics consisted of groups of cells with small cell numbers and transitioning between all four modules.

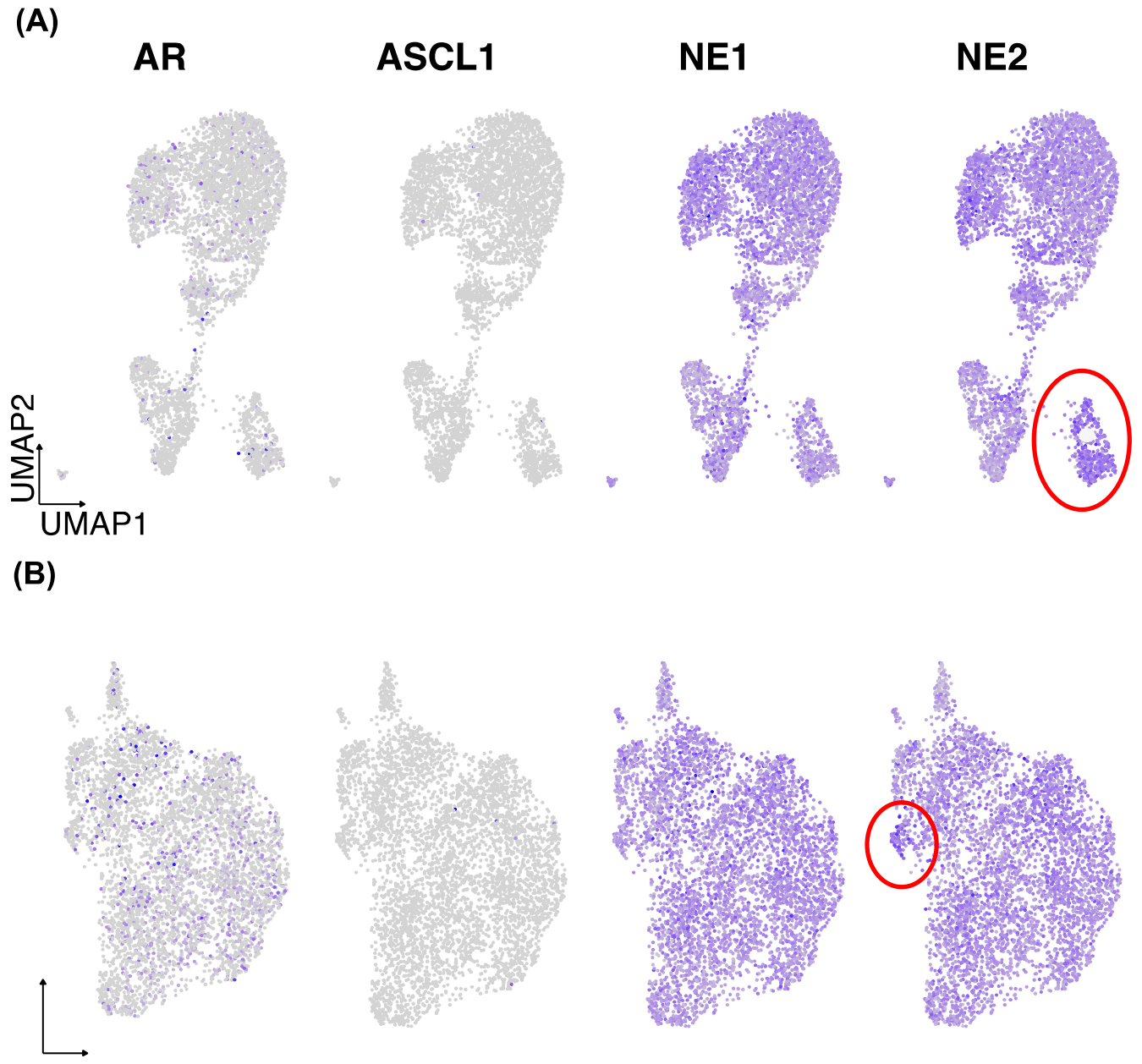

Supplementary Figure 12: Expression of NE1 and NE2 signatures in tumour cells of CA90 primary tumours.

(A) Expression of AR, ASCL1, NE1 and NE2 signatures in tumour only cells of CA90 primary prostate tumour site 13 (n = 4,736 cells). NE2 expression was higher in the right small cluster than in the rest of the cells.

(B) Expression of AR, ASCL1, NE1 and NE2 signatures in tumour only cells of CA90 primary prostate tumour site 18 (n = 4,715 cells), showing homogenous expression of NE1 and NE2 signatures.

Tumour cells were separated from normal cells through SingleR annotations and InferCNV results.

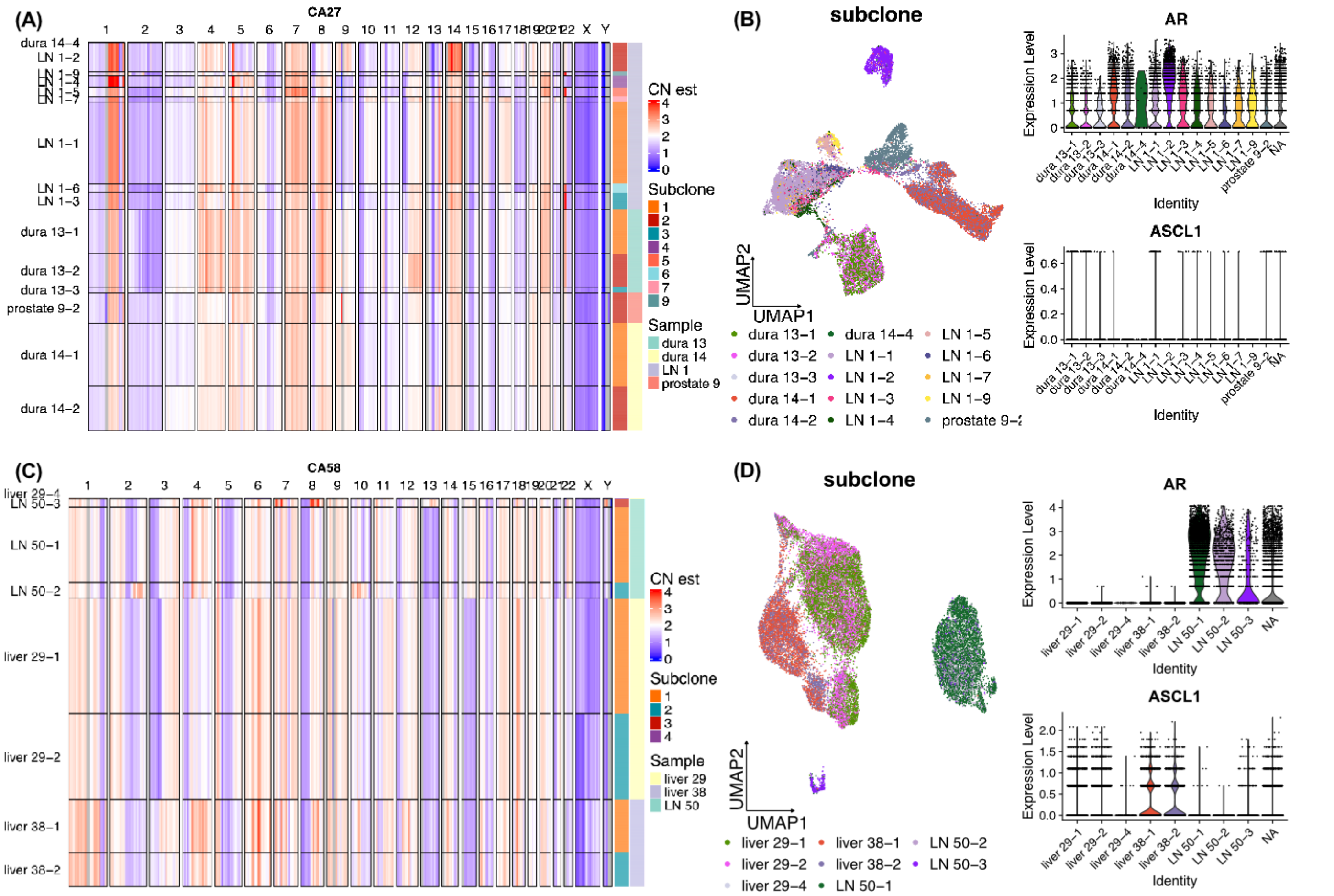

Supplementary Figure 13: Subclone overview for CA27 and CA58 with AR and ASCL1 expression.

(A) Joint subclones inferred by ATAClone for all lesions of CA27 (n = 4 samples). The subclones shared most background CNVs originated from the same ancestor. The evolutionary relationship of metastases was not linear, with subclones in the different metastases not being direct daughters of one another. The LN sample has the greatest number of subclones and distinct amplifications.

(B) UMAP of tumour cells of CA27 (n = 20,585 cells: dura = 2 samples of 10,131 cells [6 subclones], LN = 8,834 cells [8 subclones], prostate = 1,620 cells[1 subclone]) with expression of *AR* and *ASCL1* grouped by subclones. All the subclones have comparable *AR* expression. *ASCL1* expression is also shared across subclones, suggesting the emergence of NE subpopulations was spontaneous across subclones. Inter-lesion clusters are mostly, but not always, dominated by one subclone.

(C) Joint subclones inferred by ATAClone for all lesions of CA58. Livers and LN 50 subclone 3 cells have more CNV than the rest subclones. Although the liver samples have NE signatures, they are distinct clones. The LN 50 subclone 3 is the most distinct subclone.

(D) UMAP of integrated tumour cells of CA58 (*n* = 21,218 cells across 8 subclones: liver = 15,787 [5 subclones]; LN = 5,431 [3 subclones]) with expression of *AR* and *ASCL1* grouped by subclones. There is a cluster in NE cells in the LN sample, which all belong to subclone 3. This subclone has both *AR*+ and *ASCL1*+ cells. Their CNV profile is more similar to the other subclones of LN 50 rather than the liver clones, indicating that emergence of NE features in this subclone was separate to the emergence of NE in the liver samples.

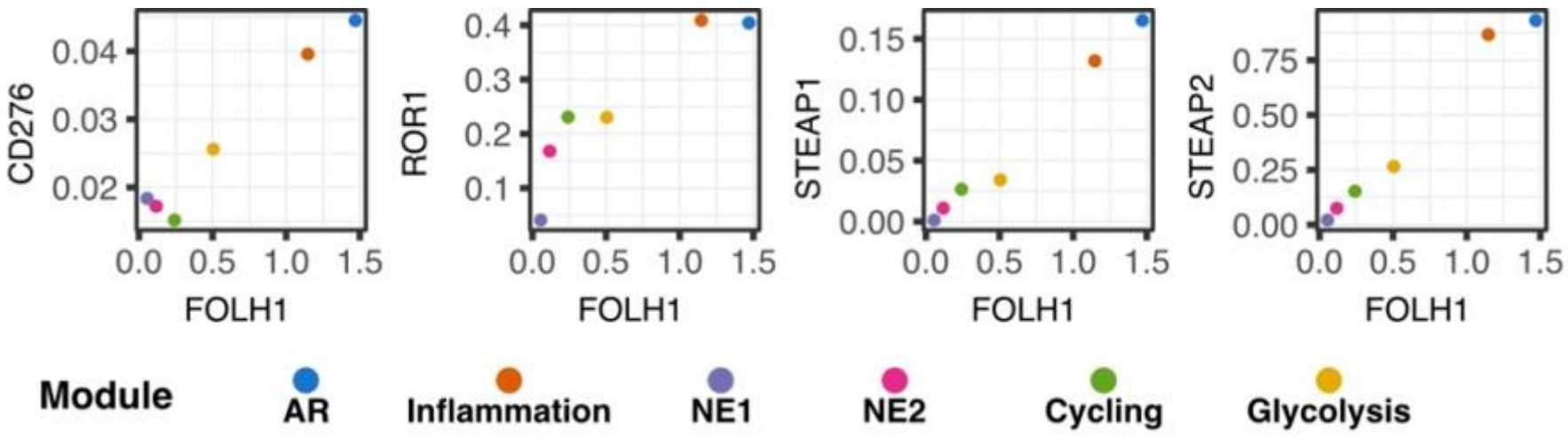

Supplementary Figure 14: Expression of *STEAP1, STEAP2*, *ROR1* and *CD276* relative to *FOLH1* across archetype module cell populations.
While *STEAP1*, *STEAP2* and *CD276* have similar patterns to *FOLH1*, *ROR1* is also expressed in the NE2, Glycolysis and Cycling modules, suggesting agents using *ROR1* as a target would potentially have an effect across a broader range of cells.

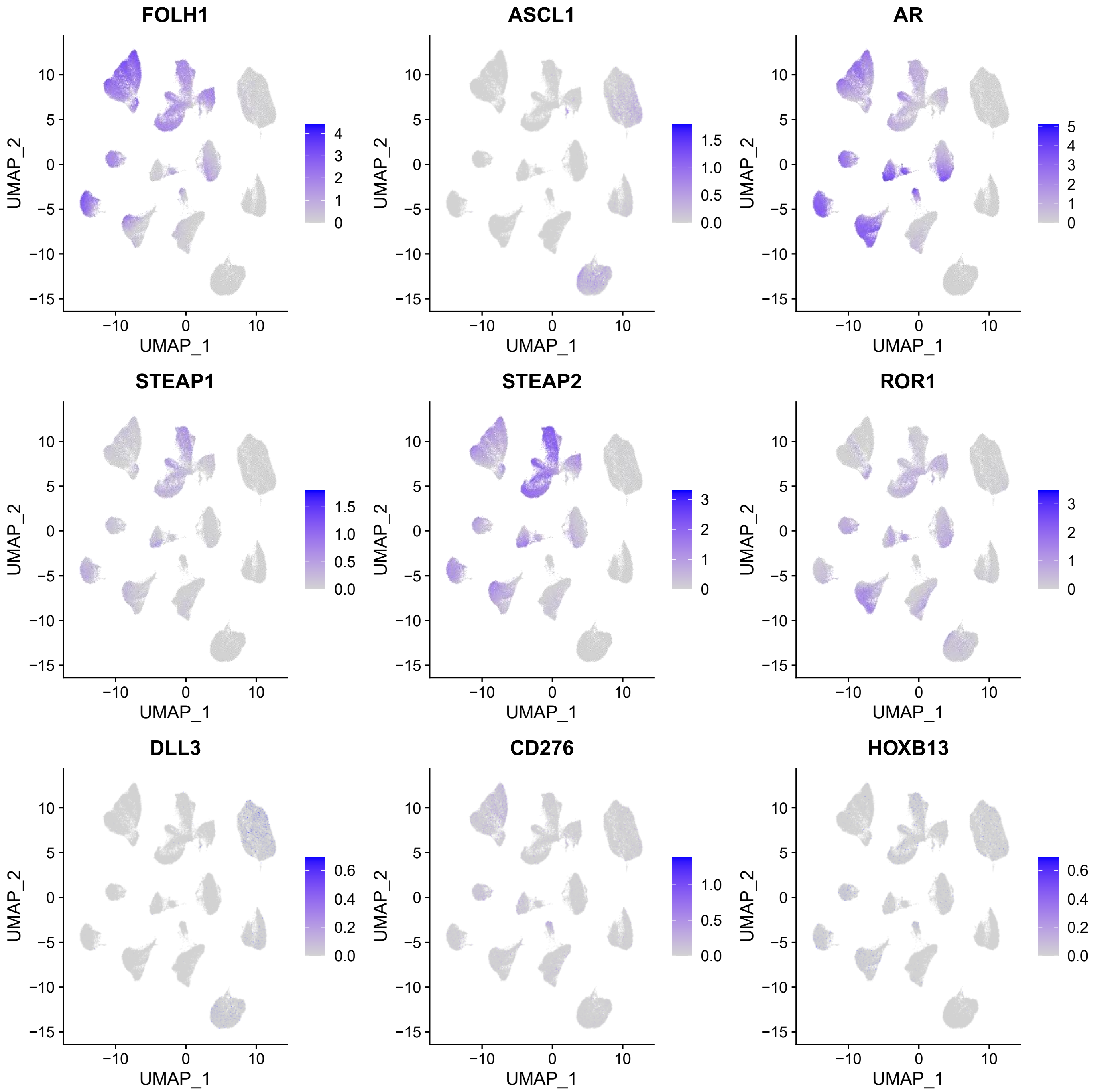

Supplementary Figure 15: Expression of therapeutic targets including *FOLH1*, *STEAP1/2*, *ROR1*, *DLL3*, *CD276*, *HOXB13* and mCRPC phenotypic markers (*AR* and *ASCL1*) across tumour cells (n = 164,726 cells) in our cohort (n = 34 samples).

FOLH1 had the strongest expression, followed by *STEAP2* and *STEAP1*. *STEAP2/1* had high expression in ARlow samples (e.g. CA27), which have the most consistent expression to *FOLH1*. *ROR1* had limited expression except CA76 samples with low *FOLH1* expression. *CD276* and *HOXB13* had very weak expression across cells with their relation to *FOLH1* expression remains unclear in this cohort. *DLL3*, a potential NE marker, had a sparse low expression in NE samples. Except *CD276* and *HOXB13* with barely any expression, *STEAP1/2* had a largest potential to be complementary to *FOLH1* therapeutic range while *DLL3* could target *FOLH1* negative populations (NE samples).

Supplementary Note 1
Identifying and subtyping non-tumour cells

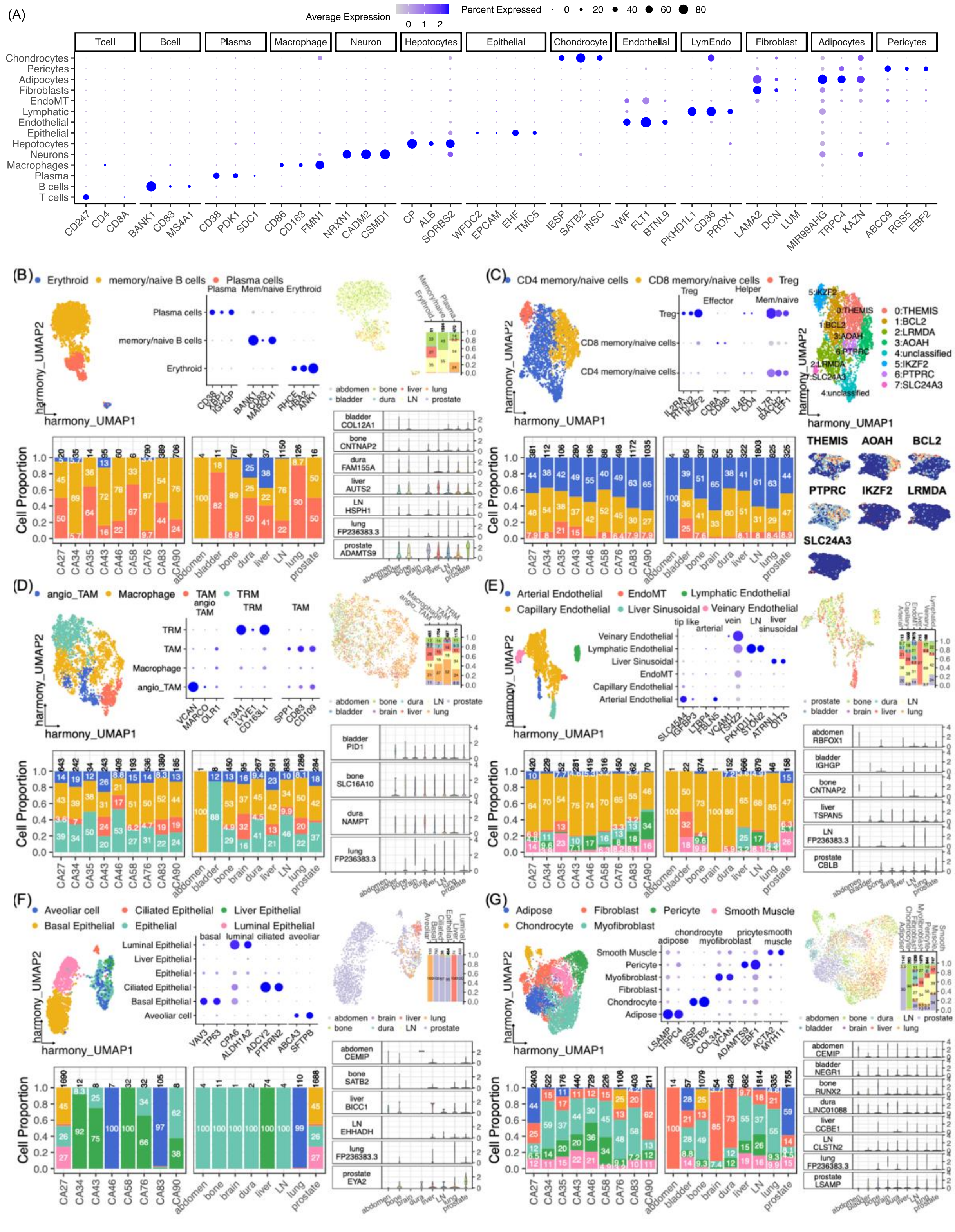

There were in total 36,108 normal cells across all our samples, which included immune populations, such as T cells, B cells, plasma cells, and macrophages, stromal populations, such as fibroblasts, adipocytes, endothelial cells and pericytes, as well as epithelial cells and neurons, and organ-specific normal cells such as hepatocytes, chondrocytes (Fig. 3E).

The cell types were identified through typical markers explained in next sections and shown in (A). B cells (n = 2,115 cells) were characterized by *MS4A1* (CD20), *CD83* expression and *BANK1* which involves in B cell signalling pathway[1,2]. T cells (n = 3868 cells) remained poorly differentiated as there was a weak signal from the cytotoxic marker *CD8A* and the CD4+ T cell marker *CD4,* but there was overexpression of *CD247*[3]. We also identified macrophage cells (n = 3865 cells) based on the expression of *CD86*, *CD163* and *FMN1*. The proportion of T cells and B cells was concordant with

the cell proportion estimate from histological staining with CD3, CD8 and CD20 but a non-significant correlation was found between the proportion of macrophages and CD68 stain intensity (Supplementary Note 4).

We also identified large clusters of cells (n = 2299 cells) expressing typical fibroblast markers *DCN*, *LUM*, and *LAMA2*. Collagen genes were largely expressed in fibroblasts but also in other fibroblast-like cells such as adipocytes and pericytes[4,5] (A). Adipocytes were characterized by having a high expression of *LAMA2*, *MIR99AHG*, *TRPC4* and *KAZN,* while pericytes upregulated *ABCC9*, *RGS5* and *EBF2* instead. *VWF* was used to annotate endothelial cells (n = 2299 cells) and the expression of *BTNL9* and *FLT1* highlighted the vascular endothelial growth and angiogenesis associated with tumour growth[6–8]. Lymphatic endothelial cells had specific marker expression (*PKHD1L1*, *CD36*, *PROX1*) and separated from common endothelial cells (A).

Hepatocytes showed distinct typical signatures like *CP*, *ALB* and *SORBS2* (A). Neurons were enriched with astrocytes makers such as *NRXN1*, and also genes associated with neuron development (*CSMD1*), and excitatory signaling (*CADM2*) [9–12]. Epithelial cells (n = 1894 cells) were identified as expressing with *WFDC2* and *EPCAM* but also upregulated cancer-associated genes like *EHF*, *TMC5* [13,14]. Chondrocytes, or osteoblasts, if considering the source of this cell type was bone, upregulated *IBS, P SATB2* and *INSC* (A).

To dive deeper into the functional status and immune subtypes, we extracted and re-analysed each immune population separately (B-D). As B cells (n = 2,115 cells) primarily expressed naïve and memory markers like *BANK1*, *CD83*, and *MARCH1*, subclustering did not enable the identification of cell subtypes other than plasma cells (n = 470 cells) (B) [2,15]. T cells (n = 3868 cells) also overexpressed naïve markers, and fully differentiated CD4 and CD8 T cells were not revealed by subclustering, but regulatory T cells with markers *IL2RA*, *RTKN2*, and *IKZF2* were identified and mostly found in CA83 and CA90[3,16] (C). Although lack of canonical T cell subtypes, DEA between T cells subclusters found signatures linked with defective immune response and immune-suppressive behaviour like *THEMIS* and *BCL2* [17,18] (C). Tumour-associated macrophages (TAM) (n = 497 cells), with markers *SPP1*, *CD83* and *CD109* and even a small group of cells (n = 485) with additional angiogenesis markers like *VCAN*, dominated the macrophage population (D). The rest of cells were either tissue-resident macrophages (TRM) (*F13A1*, *LYVE1* and *CD163L1*) or macrophages with no clear subtype markers[19]. The majority of macrophages were found in CA83 (36%) and mostly in lung samples (33%) (D).

We followed a similar procedure with stromal cells, and, contrary to immune populations, we identified organ-specific subtypes and distribution (E-G). For endothelia cells (n = 2299 cells), liver and LN had specific endothelial cell types while artery endothelial cells were found to be enriched in LN and bone, suggesting a role on immune modulation with active angiogenesis (E). Epithelial cells (n = 1894 cells) in the dataset primarily belonged to the prostate of CA27 sample. We identified a number of epithelial subtypes, including basal (*VAV3*, *TP63*) (n = 760 cells), luminal (*CPA6*, *ALDH1A2*) (n = 459 cells) and ciliated (*ADCY2*, *PTPRN2*) (n = 38 cells) subtypes[20] (F). Organ specific epithelial cells, although a small population, were also found in liver and lung as liver epithelial cells (*BICC2*, *GLIS3*, *CTNND2*) (n = 74 cells) and alveolar epithelial cells (*ABCA3*, *SFTPB*) (n = 109 cells), respectively (F). The cluster of fibroblast cells was the largest stromal cell population, and included several subtypes, including adipose (n = 1141 cells), chondrocytes (n = 283 cells), pericytes (n = 844 cells), myofibroblasts (n = 1973 cells) and smooth cells (n = 747 cells). Except adipocytes (*LSAMP*, *TRPC4*) and chondrocytes (*IBSP*, *SATB2*), which were primarily in CA27 prostate and CA76 bone, other subtypes such as pericytes (*ADAMTS9*, *EBF1*), myofibroblasts (*COL3A*, *VCAN*), and smooth muscle (*ACTA2*, *MYH1*) were seen mostly seen in liver and LN (F) [4,5,21]

Supplementary Note 2
NE1 and NE2 dissect NE phenotype plasticity

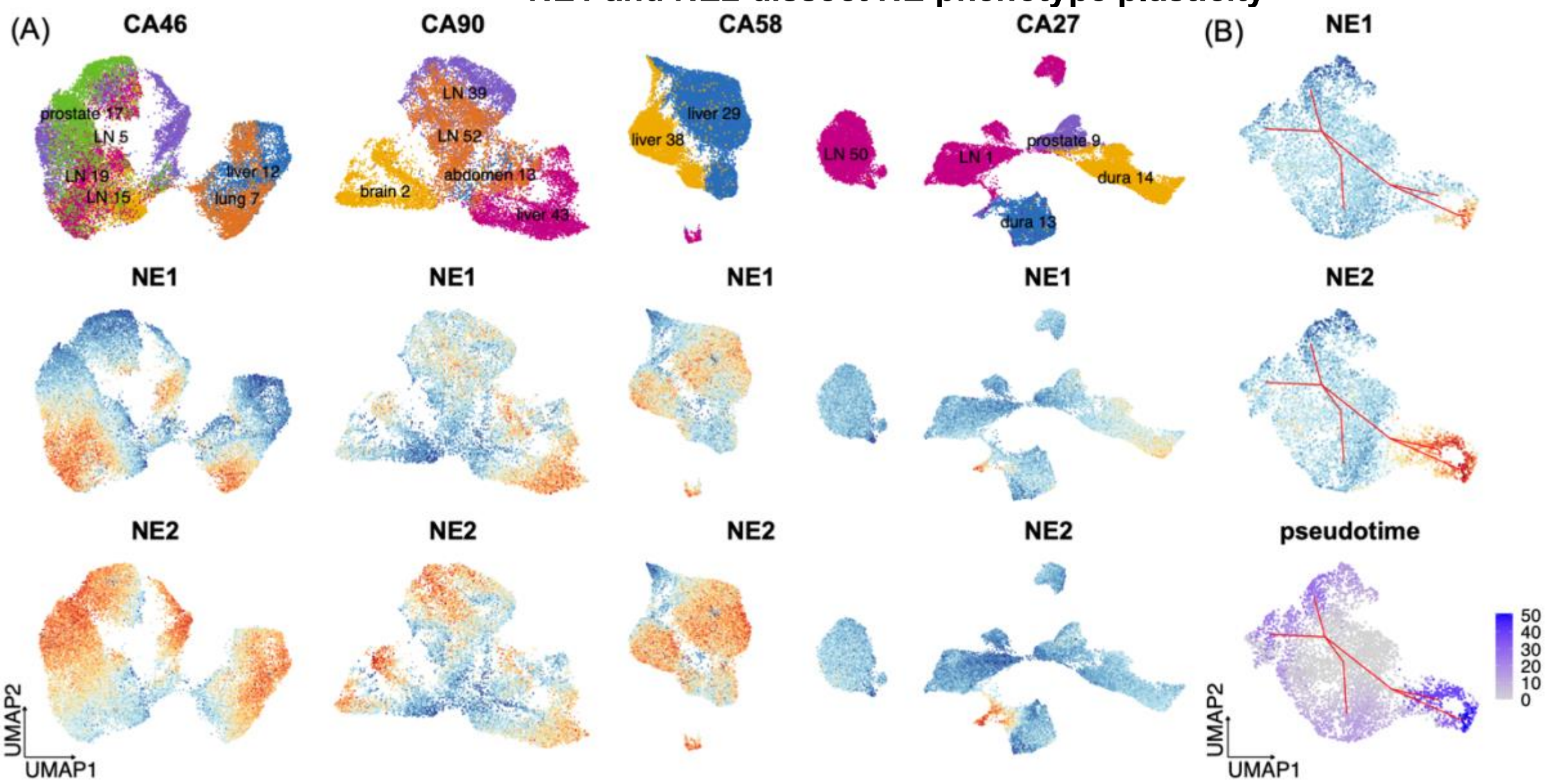

We aimed to investigate whether the NE1 and NE2 Modules represented different stages of NE development. There was high expression of both NE1 and NE2 Modules in purely NE samples such as lesions from CA46 and CA90 and liver lesions from CA58 (A). Same cells populations of the 4 patients as in Supplementary Figure 2 were shown. However, there were marked differences in the patterns of expression within each lesion, with solely high expression of NE1 or 2 ($NE1^{high}$ or $NE2^{high}$), or a combination in a subset of cells (NE^{mixed}). We therefore hypothesized that NE1 might represent a more differentiated NE state better correlated with *ASCL1*, while NE2 might represent a transitory, plastic state from adenocarcinoma. We checked the expression of the signatures in primary tumours of CA90 prostate and found positive expression of NE1 and NE2 Modules in the low AR and ASCL1 negative context (Supplementary Figure 6). Here, the NE2 Module showed specifically higher expression in a small group of cells which could be undergoing a transition process similar to CA27 (A).

To dissect this, we investigated the lesions from CA27, as all lesions had a combination of both phenotypes. We first focused on the CA27 dura 13 sample since the development of NE phenotype from amphicrine cells was free from subclone evolution in this sample (B). To capture the developmental expression trajectory from adenocarcinoma cells to NE cells, we performed pseudotime analyses and estimated the trajectory paths between archetype module cells, using the AR module cells as the starting point. Cells were divided into $NE2^{high}$, NE^{mixed} and $NE1^{high}$ groups. We found that NE2 had a high expression level in a larger cell population than the NE1 signature (B). Pseudotime analyses identified that NE^{mixed} cells showed the furthest distance from the AR module, suggesting a more matured state when there was co-expression of both signatures (Fig. 4D). In contrast, $NE2^{high}$ cells were positioned between the AR module and NE^{mixed} cells while $NE1^{high}$ cells were rare in this sample, considering its expression in other NE samples in patients with clinical diagnosed NE like CA46 and CA90. Overall, NE1 and NE2 signatures indicate different NE states.

Supplementary Note 3
Evaluation of NE1 and NE2 signatures in external and pre-clinical public data sets

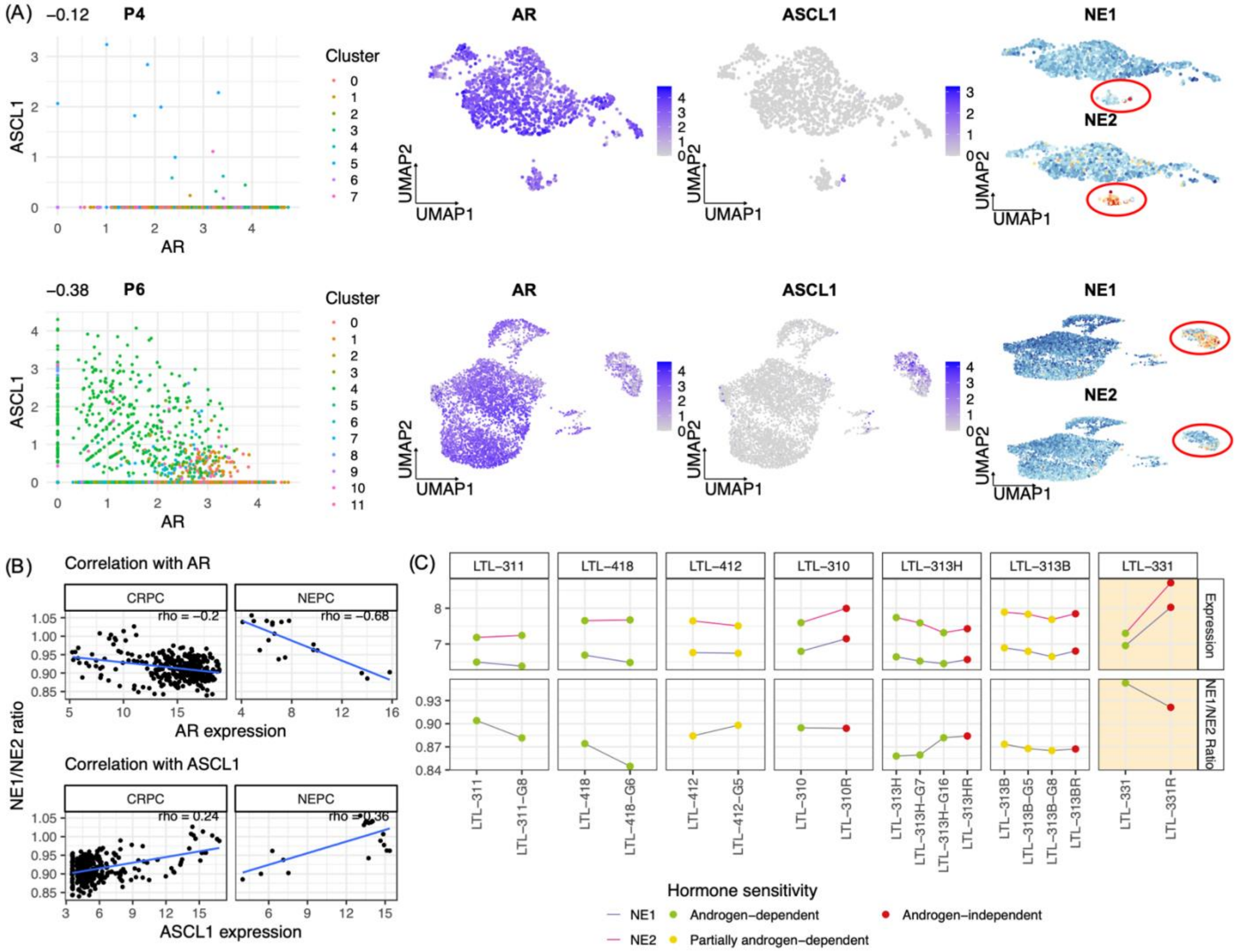

We investigated the expression of NE1 and NE2 signatures in external datasets. First, we investigated the single-cell study by Dong et al. who profiled six mCRPC patients, including two tumours (patients 4 and 6) that were positive for AR and INSM1 on histology[22] (A). As expected, in both patients there were cells co-expressing *AR* and *ASCL1*, suggesting a transition state. In cells of Patient 4 (n = 1319 cells), where there were fewer amphicrine cells and lower *ASCL1* levels, with overexpression of NE2. In contrast, in Patient 6 (n = 5197 cells), cells with higher *ASCL1* had higher expression of NE1 than NE2. The observation was concordant with our hypothesis that NE1 and NE2 describe two NE subtypes with NE1 more linked with *ASCL1* expression, while NE2 is more linked with transitioning, and NE^{mixed} is the most differentiated state (A).

We also investigated the expression of these signatures in CRPC samples from Prostate Cancer Atlas[23] (B). NE1/NE2 ratios were higher in low AR samples, in both CRPC (n = 632 samples) and NEPC (n = 38) samples, with the trend being strongest in NEPC samples. Similarly, NE1/NE2 ratios increased with *ASLC1* levels. This is consistent with NE1 showing its highest expression at end-stages of NEPC development (low/no AR and high *ASCL1*), while NE2 is higher during NE transitioning stage where both positive *AR* and *ASCL1* (lower than end-stage NE) was observed, consistent with emerging earlier in neuroendocrine development.

In the xenograft models developed by Living Tumour Lab[24] treated with testosterone (GSE41193), a subset develops androgen-independence, and one model developed NEPC (LTL-331) (C). The model that developed NEPC showed the highest NE1/NE2 ratio at baseline. This ratio subsequently decreases with androgen independence but is still the highest of all xenografts. This suggest that NE1 and NE2 are sensitive to detect NEPC transformation at early stages of disease development.

Supplementary Note 4
Correlation between cell type proportions inferred from snRNA data and from immunohistochemistry (IHC)

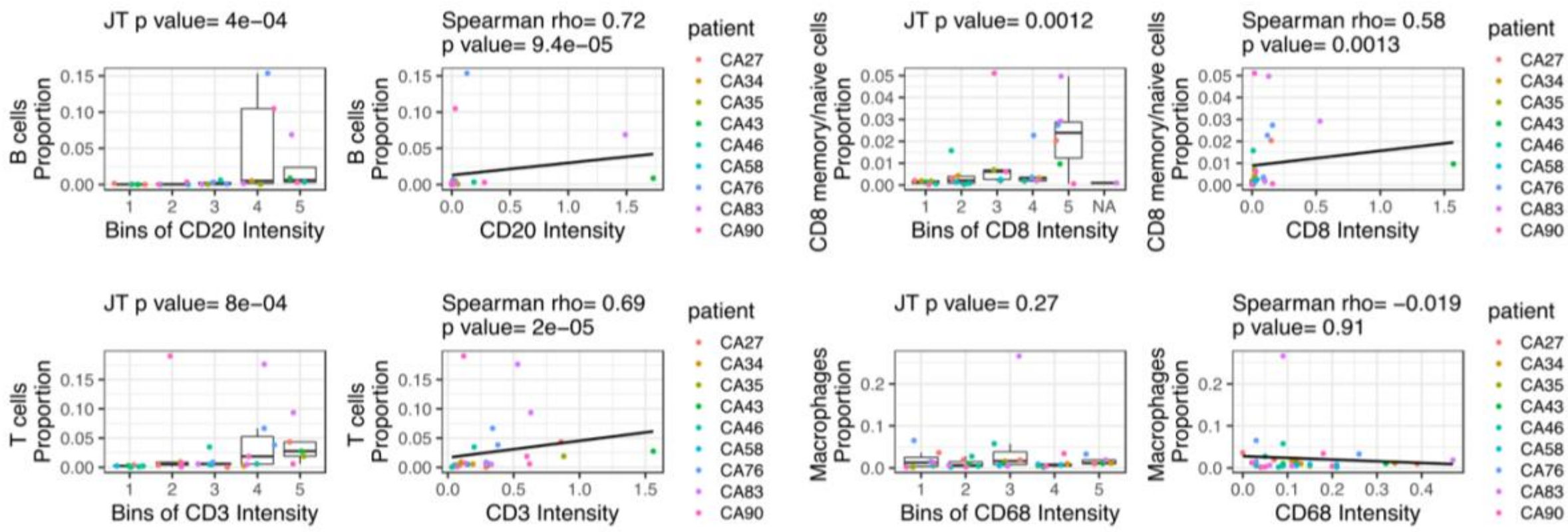

To validate the proportion of microenvironment cells in our data that were derived from the snRNAseq data, we performed staining of CD20, CD8, CD3 and CD68, to quantify B cells, CD8 T cells, T cells and macrophages on matched FFPE tissue sections. These show significant positive correlations, except between CD68 staining and macrophages. The IHC staining was done on slides of the same tissue samples as the fresh frozen samples used to generate the snRNAseq data. Therefore, complete matched cell composition was not expected. The Jonckheere-Terpstra (JT) and Spearman correlation tests were used to quantify the relationship between the IHC stain positive area proportion and the corresponding cell type proportion to the total cells of tissue.

Supplementary References

1. Morgan, D. & Tergaonkar, V. Unraveling B cell trajectories at single cell resolution. *Trends Immunol.* **43**, 210–229 (2022).
2. Gómez Hernández, G., Morell, M. & Alarcón-Riquelme, M. E. The Role of BANK1 in B Cell Signaling and Disease. *Cells* **10**, 1184 (2021).
3. Szabo, P. A. *et al.* Single-cell transcriptomics of human T cells reveals tissue and activation signatures in health and disease. *Nat. Commun.* **10**, 4706 (2019).
4. Lendahl, U., Muhl, L. & Betsholtz, C. Identification, discrimination and heterogeneity of fibroblasts. *Nat. Commun.* **13**, 3409 (2022).
5. Single-cell analysis reveals prognostic fibroblast subpopulations linked to molecular and immunological subtypes of lung cancer | Nature Communications. https://www.nature.com/articles/s41467-023-35832-6.
6. Akil, A. *et al.* Notch Signaling in Vascular Endothelial Cells, Angiogenesis, and Tumor Progression: An Update and Prospective. *Front. Cell Dev. Biol.* **9**, (2021).
7. Lee, H. K., Chauhan, S. K., Kay, E. & Dana, R. Flt-1 regulates vascular endothelial cell migration via a protein tyrosine kinase-7–dependent pathway. *Blood* **117**, 5762–5771 (2011).
8. A Guide to Endothelial Cell Markers. https://www.biocompare.com/Editorial-Articles/598462-A-Guide-to-Endothelial-Cell-Markers/.
9. Kraus, D. M. *et al.* CSMD1 is a novel multiple domain complement-regulatory protein highly expressed in the central nervous system and epithelial tissues. *J. Immunol. Baltim. Md 1950* **176**, 4419–4430 (2006).
10. Pasman, J. A. *et al.* The CADM2 Gene and Behavior: A Phenome-Wide Scan in UK-Biobank. *Behav. Genet.* **52**, 306–314 (2022).
11. PubChem. KCNIP4 - potassium voltage-gated channel interacting protein 4 (human). https://pubchem.ncbi.nlm.nih.gov/gene/KCNIP4/human.
12. Tan, C. X. & Eroglu, C. Cell adhesion molecules regulating astrocyte-neuron interactions. *Curr. Opin. Neurobiol.* **69**, 170–177 (2021).
13. Zhang, H., Zhang, X., Xu, W. & Wang, J. TMC5 is Highly Expressed in Human Cancers and Corelates to Prognosis and Immune Cell Infiltration: A Comprehensive Bioinformatics Analysis. *Front. Mol. Biosci.* **8**, 810864 (2022).
14. Wang, L. *et al.* EHF promotes colorectal carcinoma progression by activating TGF-β1 transcription and canonical TGF-β signaling. *Cancer Sci.* **111**, 2310–2324 (2020).
15. Krzyzak, L. *et al.* CD83 Modulates B Cell Activation and Germinal Center Responses. *J. Immunol. Baltim. Md 1950* **196**, 3581–3594 (2016).

16. Luo, Y. *et al.* Single-cell transcriptomic analysis reveals disparate effector differentiation pathways in human Treg compartment. *Nat. Commun.* **12**, 3913 (2021).
17. Liu, L. *et al.* BCL-2 expression promotes immunosuppression in chronic lymphocytic leukemia by enhancing regulatory T cell differentiation and cytotoxic T cell exhaustion. *Mol. Cancer* **21**, 59 (2022).
18. Prasad, M. *et al.* Themis regulates metabolic signaling and effector functions in CD4+ T cells by controlling NFAT nuclear translocation. *Cell. Mol. Immunol.* **18**, 2249–2261 (2021).
19. Ma, R.-Y., Black, A. & Qian, B.-Z. Macrophage diversity in cancer revisited in the era of single-cell omics. *Trends Immunol.* **43**, 546–563 (2022).
20. Nguyen, Q. H. *et al.* Profiling human breast epithelial cells using single cell RNA sequencing identifies cell diversity. *Nat. Commun.* **9**, 2028 (2018).
21. Xie, T. *et al.* Single-Cell Deconvolution of Fibroblast Heterogeneity in Mouse Pulmonary Fibrosis. *Cell Rep.* **22**, 3625–3640 (2018).
22. Dong, B. *et al.* Single-cell analysis supports a luminal-neuroendocrine transdifferentiation in human prostate cancer. *Commun. Biol.* **3**, 1–15 (2020).
23. Bolis, M. *et al.* Dynamic prostate cancer transcriptome analysis delineates the trajectory to disease progression. *Nat. Commun.* **12**, 7033 (2021).
24. Lin, D. *et al.* High fidelity patient-derived xenografts for accelerating prostate cancer discovery and drug development. *Cancer Res.* **74**, 1272–1283 (2014).
