# Supplementary material for: Recurrent intra-tumour heterogeneity is a hallmark of metastatic prostate cancer
Source: Nat Commun. 2026 Jun 19;17:7727. doi: 10.1038/s41467-026-74334-z (PMC13434311; doi:10.1038/s41467-026-74334-z)
Supplement: Supplementary file 2 [file 41467_2026_74334_MOESM2_ESM.pdf]

Description of Additional Supplementary Information

Title: Supplementary Data 1: Pathology and histology review records.
Description: All the samples collected for the study with the pathology and histology review results. The best quality samples based on low fat and necrosis percentage, with high tumour purity and organ diversity were selected for the study indicated in the “Selected” column. The "Labels" column lists the naming used in the main text for the samples included in the study.

Title: Supplementary Data 2: Public prostate cancer signatures.
Description: Given the diversity of genes in each signature depending on source, we curated signatures to include genes recurrent in over 50% of the available signatures with similar functions, The signatures finally used are beneath the gene set categories.

Title: Supplementary Data 3: Likelihood ratio test to determine the effect of organ on the variance of the expression of signatures.
Description: Linear regression between the sample average expression of signatures (Table S2) and patient and organ was compared using likelihood ratio tests. The null hypothesis was that only the patient explained the variance was tested against the alternative that both patient and organ explained the variance better. The results showed that adding the organ variable to the model did not significantly improve the models to predict signature expression levels for any the signatures. Global p values were ajusted using the BH method.

Title: Supplementary Data 4: Determining copy number affected differentially expressing genes between intra-patient lesions.
Description: Differential expression analysis was conducted between different samples within patients by the FindAllMarker() function with MAST method in Seurat (v4.3) with the MAST method. The differentially expressing genes (DEGs) were identified as CN affected if the sign of log fold-change agreed with the CN ratio (sample vs patient average). "Nodeg" column shows the number of DEGs for a certain sample while "degnoCN" shows the number of DEGs not covered by WGS CN calling regions. The "sign" column is the sign of avg_log2FC while "CN" shows whether "CN_ratio" is greater than 1 or not. "id" column combines "sign" and "CN" column and detemines the CN effect on DEGs. "pos >1" and "neg <1" are the cases of positive effect of CNV on gene expression. Correlation between "CN_ratio" and "avg_log2FC" was calculated for each sample and recored in the "cor" column. Log2FC cutoff was 0.25 and adjusted p value cutoff was 0.05.

Title: Supplementary Data 5: DEGs between subclones within samples.
Description: Differntial expression analysis was conducted through the FindMarkers() function with MAST method of Seurat (v4.3) in a pairwise manner between subclones within individual sample. The comparision between subclones was recorded in the "comp" column with subclones IDs seperated by "|". Log2FC cutoff was 0.25 and adjusted p value cutoff was 0.05.

Title: Supplementary Data 6: Significant DEGs between tumour lesions at different organs of metastasis.
Description: Differential expression analysis using FindAllMarkers() with MAST method of Seurat (v4.3) was conducted between different lessions using integrated tumour-only cells. Only significant DEGs are shown. Log2FC cutoff was 0.25 and adjusted p value cutoff was 0.05.

Title: Supplementary Data 7: Testing the association between cell type proportions and metastatic lesions through linear modelling.
Description: Sample cell type proportions were calculated by dividing cell type counts by total sample normal cell counts. The proportion was logit transformed and tested against ~ patient + sites through linear regression test. All other nested models were tested also, but this one showed the least mean AIC and median sigma. The global p values were corrected with BH. Adjusted p values smaller than 0.05 indicate the cell types distributing differentially across metastatic lesions.

Title: Supplementary Data 8: Archetype module signatures.
Description: Signatures for each module generated from recurrent DEGs through FindMaker() MAST method in the top 30% cell populations of each archetype in each sample. Only gene modules composed of more than 10 genes while exclusively representing only one module were retained. Small modules that recapitulated similar signatures of other modules or only found in sample-specific subpopulations were excluded.

Title: Supplementary Data 9.1: Average motif fold enrichment across samples between tumour cells with high and low expression of archetype modules.
Description: Differentially open peaks found between tumour cells with the top 30% expression of an archetype module vs the rest of the cells, and potential motifs associated to the peaks. The analysis was done per sample. Only the motifs found significant in more than 3 samples were shown in the table. The cutoff for adjusted p values was 0.05. The average fold enrichment was calculated across all 22 samples regardless of p values.

Title: Supplementary Data 9.2: Number of samples where the motifs were found to be significant.
Description: There were 22 samples found signifcant differentially expressing peaks between tumour subpopulations with various levels of archetype module expression. Differentially open peaks were found between subpopulations with top 30% expression and the rest and potential motifs attaching to the peaks. Only the motifs found significant in more than 3 samples were kept. The cutoff for adjusted p values was 0.05.

Title: Supplementary Data 10: FOLH1 gene SNVs
Description: Variants were written in the order of chromosome, position, reference nucleotide, and altered nucleotide. Variants were called with both Strelka2Pass v0.2 and FreeBayesSomatic v0.1.1 as described in the method section.

Title: Supplementary Data 11: ATAClone binned regions with differential CN estimate between subclones within samples.

Description: Regions with differential CN estimate were determined through the FindMarkers() function with MAST method of Seurat (v4.3) in a pairwise manner between subclones within individual samples. The single-cell CN estimate output from ATAClone were used. The comparison between subclones were recorded in the "comp" column with two subclones separated by "|". Log2FC cutoff was 0.25 and adjusted p value cutoff was 0.05. The genes located in the corresponding regions were listed in "gene" column.

Title: Supplementary Data 12: Significant motifs enriched on differentially open peaks between cells with different levels of FOLH1 expression groups for individual samples.

Description: In each sample, tumour cells were seperated by FOLH1 expression into "high", "low", and "neg" groups. Cells in the "high" group have expression of FOLH1 higher than the mean expression while those in the "low" group have lower expression than the mean but greater than 0. Negative group has 0 FOLH1 expression. The motifs were found using FindMotif() through permutation test on the differentially open peaks between different FOLH1 groups.
