# Supplementary material for: Recurrent intra-tumour heterogeneity is a hallmark of metastatic prostate cancer
Source: Nat Commun. 2026 Jun 19;17:7727. doi: 10.1038/s41467-026-74334-z (PMC13434311; doi:10.1038/s41467-026-74334-z)
Supplement: Supplementary file 3 [file 41467_2026_74334_MOESM4_ESM.pdf]

nature portfolio

Corresponding author(s): Anna Trigos, Shahneen Sandu

Last updated by author(s): 08-05-2026

Reporting Summary

Nature Portfolio wishes to improve the reproducibility of the work that we publish. This form provides structure for consistency and transparency in reporting. For further information on Nature Portfolio policies, see our Editorial Policies and the Editorial Policy Checklist.

Statistics

For all statistical analyses, confirm that the following items are present in the figure legend, table legend, main text, or Methods section.

n/a	Confirmed	
☐	☒	The exact sample size (n) for each experimental group/condition, given as a discrete number and unit of measurement
☐	☒	A statement on whether measurements were taken from distinct samples or whether the same sample was measured repeatedly
☐	☒	The statistical test(s) used AND whether they are one- or two-sided<br>*Only common tests should be described solely by name; describe more complex techniques in the Methods section.*
☐	☒	A description of all covariates tested
☐	☒	A description of any assumptions or corrections, such as tests of normality and adjustment for multiple comparisons
☐	☒	A full description of the statistical parameters including central tendency (e.g. means) or other basic estimates (e.g. regression coefficient) AND variation (e.g. standard deviation) or associated estimates of uncertainty (e.g. confidence intervals)
☐	☒	For null hypothesis testing, the test statistic (e.g. F, t, r) with confidence intervals, effect sizes, degrees of freedom and P value noted<br>*Give P values as exact values whenever suitable.*
☒	☐	For Bayesian analysis, information on the choice of priors and Markov chain Monte Carlo settings
☐	☒	For hierarchical and complex designs, identification of the appropriate level for tests and full reporting of outcomes
☐	☒	Estimates of effect sizes (e.g. Cohen's d, Pearson's r), indicating how they were calculated

Our web collection on statistics for biologists contains articles on many of the points above.

Software and code

Policy information about availability of computer code

Data collection

Genomics and single-cell data was generated from rapid autopsy samples using protocols described in the methods.

WGS processing pipeline and variants extraction:
FASTQC v0.11.8
Cutadapt v4.1
BWA-MEM v0.7.17
Picard tools v2.17.3
Strelka2Pass v0.2 (with strelka.ini 711 configuration file)
FreeBayesSomatic v0.1.19
BCFtools v1.9
GATK v3.61
VEP v98
CADD v1.5
Condel
DBNSFP v4.0a
gnomAD v3.0
GRIDSS v2.13.2
gridss_somatic_filter R script (https://github.com/PapenfussLab/gridss/blob/master/scripts/gridss_somatic_filter)
Hartwig Medical Foundation's DNA pipeline v5.32

AMBER v3.5
COBALT v1.11
PURPLE v3.1
CHORD20, HRDetect21
sigrap v0.1.0
mutSigExtractor v1.27
StructuralVariantAnnotation v1.10.1

Single-nucleus RNA-seq processing:
Cell Ranger v5.0.0
Cell Ranger ARC v2.0.0
Cell Ranger v7.1.0
EmptyDrops
Seurat v4.1.0
scran v1.26.2
scDblFinder v1.9.2
SingleR v1.8.1
celldex v1.4.0

Data analysis

The data analysis was done with R 4.2.0 using customized codes available to public in https://github.com/TrigosTeam/Recurrent-Tumour-Single-Cell

Data Preprocessing:
dplyr v1.1.4
data.table v1.15.4
readr v2.1.5
readxl v1.4.3
dbplyr v2.5.0
stringr v1.5.1
purrr v1.0.2
tibble v3.2.1
reshape2 v1.4.4
plyr v1.8.9
tools v4.2.0
parallel v4.2.0
Matrix v1.6.5
DelayedArray v0.24.0
rlist vNA
matrixStats v1.1.0
R.utils v2.12.3
tidyverse v2.0.0

Visualization Plotting:
ggplot2 v3.5.1
ggpubr v0.6.0
ggsignif v0.6.4
ggrepel v0.9.5
ggstance v0.3.7
ggh4x v0.2.8
ggExtra v0.10.1
ggridges v0.5.6
hrbrthemes v0.8.7
wesanderson v0.3.7
colorspace v2.1.0
pals v1.8
RColorBrewer v1.1.3
randomcoloR v1.1.0.1
scales v1.3.0
patchwork v1.2.0
cowplot v1.1.3
ggsci v3.2.0
geomtextpath v0.1.4
gridExtra v2.3
grid v4.2.0
ComplexHeatmap v2.14.0
heatmaply v1.5.0
swimplot v1.2.0
plotly v4.10.4

htmlwidgets v1.6.4
dendextend v1.18.0
UpSetR v1.4.0
ggvenn v0.1.15
ComplexUpset v1.3.3
circlize v0.4.16
phylogram v2.1.0
phangorn v2.11.1
vegan v2.6.6.1

Genomic analysis:
cluster v2.1.3
rankdist v1.1.4
vcfR vNA
Hmisc v5.1.3
mixtools v2.0.0
biomaRt v2.54.1
CONICSmat v0.0.0.1
GenomicRanges v1.50.2
IRanges v2.32.0

Single cell analysis:
Seurat v4.4.0
igraph v2.0.3
mmand v1.6.3
clusterProfiler v3.21
enrichplot v1.28.2
dorothea v1.10.0
decoupleR v2.4.0
matrixStats v1.1.0
ParetoTI v0.1.13
scater v1.26.1
scran v1.26.2
msigdbr v7.5.1
MAST v1.24.1
cluster v2.1.3
DescTools v0.99.49
harmony v1.2.3
SingleR v1.10.0
DOSE v3.5
scCustomize v2.1.2
dittoSeq v1.10.0
simspec v0.0.0.9000
ruvIIInb v0.7.8.8
scuttle v1.8.4
celldex v1.8.0
uwot v0.2.2
scMerge v1.14.0
reticulate v1.38.0
Rmagic v2.0.3
TSCAN v1.28.0

Analysis of external gene expression data:
limma v3.54.2
DESeq2 vNA
SummarizedExperiment v1.28.0
edgeR v3.40.2

Additional analysis for rebuttal edition in R4.5.0
broom.mixed v0.2.9.7
BSgenome.Hsapiens.UCSC.hg38 v1.4.5
CellChat v2.2.0
chromVAR v1.32.0
circlize v0.4.17
ComplexHeatmap v2.26.0
cowplot v1.2.0
data.table v1.18.0
DelayedArray v0.36.0
dorothea v1.22.0

dplyr v1.1.4
emmeans v2.0.1
EnsDb.Hsapiens.v86 v2.99.0
future v1.68.0
GenomicRanges v1.62.1
ggplot2 v4.0.1
ggpubr v0.6.2
ggrastr v1.0.2
ggridges v0.5.7
glmmTMB v1.1.14
harmony v1.2.4
infercnv v3.2.0
JASPAR2020 v0.99.10
JASPAR2024 v0.99.7
limma v3.66.0
lme4 v1.1.38
lmerTest v3.2.0
Matrix v1.7.4
motifmatchr v1.32.0
pals v1.10
patchwork v1.3.2
purrr v1.2.1
qs v0.27.3
ruvIIInb v0.9.2.1
scran v1.38.0
Seurat v5.4.0
Signac v1.16.0
speckle v1.10.0
TFBSTools v1.48.0
tidyverse v2.0.0

For manuscripts utilizing custom algorithms or software that are central to the research but not yet described in published literature, software must be made available to editors and reviewers. We strongly encourage code deposition in a community repository (e.g. GitHub). See the Nature Portfolio guidelines for submitting code & software for further information.

Data

Policy information about availability of data

All manuscripts must include a data availability statement. This statement should provide the following information, where applicable:

- Accession codes, unique identifiers, or web links for publicly available datasets
- A description of any restrictions on data availability
- For clinical datasets or third party data, please ensure that the statement adheres to our policy

The whole-genome DNA sequencing data and single-nuclei RNA-seq and ATAC-seq data generated in this study are available from the European Genome-phenome Archive under accession number EGAS50000001312 (https://ega-archive.org/studies/EGAS50000001312) with no time limit. Since the data includes patient genetic information, researchers who wish to apply for access require human ethics approval. The average response time to the access request is 14 business days. Previously published whole-genome sequencing data used in this study can be found under accession number EGAS00001006598 (https://ega-archive.org/studies/EGAS00001006598) [13].
The publicly available bulk RNA-seq data shown in Figure 5f can be found at GSE126078 (https://www.ncbi.nlm.nih.gov/geo/query/acc.cgi?acc=GSE126078)[51]. The publicly available data used in Supplementary Note 2 includes single cell data from GSE137829 (https://www.ncbi.nlm.nih.gov/geo/query/acc.cgi?acc=GSE137829)[76] , bulk RNA-seq data from E-MTAB-9930 (https://www.ebi.ac.uk/biostudies/arrayexpress/studies/E-MTAB-9930)[133] and Xenograft bulk RNA-seq data from GSE41193 (https://www.ncbi.nlm.nih.gov/geo/query/acc.cgi?acc=GSE41193)[134]. The remaining data are available within the Article, Supplementary Information or Source Data file (https://doi.org/10.5281/zenodo.20019864)[132].

Research involving human participants, their data, or biological material

Policy information about studies with human participants or human data. See also policy information about sex, gender (identity/presentation), and sexual orientation and race, ethnicity and racism.

Reporting on sex and gender	All patients included in the study were male as the focus was on metastatic prostate cancer.
Reporting on race, ethnicity, or other socially relevant groupings	Information not available
Population characteristics	Metastatic tissue was collected through the rapid-autopsy CASCADE (CAncer tiSsue Collection After DEath) program at the

	Peter MacCallum Cancer Centre in Melbourne, Australia between 2013 and 2019 from nine donors who died from metastatic castration-resistant prostate cancer. The patients were aged between 56 and 71 years old.
Recruitment	Patients were approached by their treating clinician to ask whether they would like to participate, then consented. Patients were selected based on clinical history. Criteria included patients with soft tissue disease who had previously received abi/enza, and/or patients with neuroendocrine disease.
Ethics oversight	Human Research Ethics Committee of the Peter MacCallum Cancer Centre

Note that full information on the approval of the study protocol must also be provided in the manuscript.

Field-specific reporting

Please select the one below that is the best fit for your research. If you are not sure, read the appropriate sections before making your selection.

[x] Life sciences [ ] Behavioural & social sciences [ ] Ecological, evolutionary & environmental sciences

For a reference copy of the document with all sections, see nature.com/documents/nr-reporting-summary-flat.pdf

Life sciences study design

All studies must disclose on these points even when the disclosure is negative.

Sample size	2-6 samples collected via rapid autopsy from 9 patients.
Data exclusions	Only samples with high tumour purity, low necrosis and fat content and of diverse metastatic sites were selected to be included in the study. All generated data that passed technical QC were included in the study.
Replication	Due to the nature of the work and uniqueness of the tissue, results are presented for individual patients, and common trends are highlighted.
Randomization	Not applicable to our analyses, as it is an observational, non-interventional multi-omics analysis
Blinding	Not applicable to our analyses, as it is an observational, non-interventional multi-omics analysis

Reporting for specific materials, systems and methods

We require information from authors about some types of materials, experimental systems and methods used in many studies. Here, indicate whether each material, system or method listed is relevant to your study. If you are not sure if a list item applies to your research, read the appropriate section before selecting a response.

Materials & experimental systems

n/a	Involved in the study	
[ ]	[x]	Antibodies
[x]	[ ]	Eukaryotic cell lines
[x]	[ ]	Palaeontology and archaeology
[x]	[ ]	Animals and other organisms
[x]	[ ]	Clinical data
[x]	[ ]	Dual use research of concern
[x]	[ ]	Plants

Methods

n/a	Involved in the study	
[x]	[ ]	ChIP-seq
[x]	[ ]	Flow cytometry
[x]	[ ]	MRI-based neuroimaging

Antibodies

Antibodies used	Immunohistochemical (IHC) stain was completed to show the PSMA, AR, INSM1, SYN, CGA levels of samples using Anti-PSMA Monoclonal Mouse Antibody (clone 3E6 from Dako with 1:50 dilution, stained by Ventana Discovery Ultra Automated Stainer from Ventana Medical Systems Inc., Oro valley, United States); Monoclonal Mouse anti-Human Androgen Receptor (clone AR41 from Dako with 1:50 dilution, stained by Bond RX Automated Research Stainer); INSM1 (clone A-8 from Santa Cruz Biotechnology sc-271408 with 1:400 dilution, stained by Ventana Benchmark Ultra platform, Ventana Medical Systems, Tucson, AZ); SYN: 1:50 (Ventana Benchmark Ultra platform, Ventana Medical Systems, Tucson, AZ); Monoclonal Mouse Anti-Human Chromogranin A (clone DAK-A3 from Dako with1:400 dilution, stained by Ventana Benchmark Ultra platform, Ventana Medical Systems, Tucson, AZ), respectively.

Validation	PSMA (clone 3E6): https://www.agilent.com/en/product/immunohistochemistry/antibodies-controls/primary-antibodies/prostate-specific-membrane-antigen-%28concentrate%29-76591<br>Androgen Receptor (clone AR41): https://www.agilent.com/en/product/immunohistochemistry/antibodies-controls/primary-antibodies/androgen-receptor-(concentrate)-76575<br>INSM1 (clone A-8): https://www.scbt.com/p/insm1-antibody-a-8?srsltid=AfmBOop5hqxZSJJdj40EalGWmC07HllKAbWMJwtUKVqBx25lUroE7VJj<br>Chromogranin A (clone DAK-A3): https://www.agilent.com/en/product/immunohistochemistry/antibodies-controls/primary-antibodies/chromogranin-a-(concentrate)-76546

Plants

Seed stocks	*Report on the source of all seed stocks or other plant material used. If applicable, state the seed stock centre and catalogue number. If plant specimens were collected from the field, describe the collection location, date and sampling procedures.*
Novel plant genotypes	*Describe the methods by which all novel plant genotypes were produced. This includes those generated by transgenic approaches, gene editing, chemical/radiation-based mutagenesis and hybridization. For transgenic lines, describe the transformation method, the number of independent lines analyzed and the generation upon which experiments were performed. For gene-edited lines, describe the editor used, the endogenous sequence targeted for editing, the targeting guide RNA sequence (if applicable) and how the editor was applied.*
Authentication	*Describe any authentication procedures for each seed stock used or novel genotype generated. Describe any experiments used to assess the effect of a mutation and, where applicable, how potential secondary effects (e.g. second site T-DNA insertions, mosiacism, off-target gene editing) were examined.*
