# Supplementary material for: Recurrent intra-tumour heterogeneity is a hallmark of metastatic prostate cancer
Source: Nat Commun. 2026 Jun 19;17:7727. doi: 10.1038/s41467-026-74334-z (PMC13434311; doi:10.1038/s41467-026-74334-z)
Supplement: Supplementary file 4 [file 41467_2026_74334_MOESM5_ESM.pdf]

nature portfolio

Peer Review File

Recurrent intra-tumour heterogeneity is a hallmark of metastatic prostate cancer

Corresponding Author: Dr Anna Trigos

This manuscript has been previously reviewed at another journal. This document only contains information relating to versions considered at Nature Communications.

This file contains all reviewer reports in order by version, followed by all author rebuttals in order by version.

Version 1:

Reviewer comments:

Reviewer #1

(Remarks to the Author)
In the revised version of the manuscript titled "Recurrent intra-tumour heterogeneity is a hallmark of metastatic prostate cancer," Weng et al. have addressed many of the reviewers' comments; however, several aspects of the manuscript still require revision, as they could be misleading to readers.

1. The assertion that copy number variations (CNVs) primarily contribute to transcriptional noise is overly simplistic and potentially misleading. In many cases, it is difficult to identify a single driver gene within a CNV region, as multiple genes may be simultaneously altered and contribute to the observed phenotype. While certain CNVs may have clear biological relevance linked to specific loci, many involve broader genomic regions with complex, multi-gene effects. The authors should therefore temper this claim and provide a more nuanced discussion of the interplay between genomic alterations and transcriptional states.

2. A similar concern applies to the interpretation of microenvironmental influences on cell states. The cohort analyzed here is small (9 patients), which substantially limits the generalizability of the conclusions. There is ample evidence in the literature that specific tumor cell states are enriched in particular metastatic niches (for example, neuroendocrine prostate cancer in liver metastases). The absence of such patterns in this limited dataset does not negate their biological relevance. The authors should exercise caution in making broad or generalized statements and more clearly acknowledge the limitations imposed by sample size.

3. Throughout the manuscript, several statements lack precision or are potentially misleading. The overall writing would benefit from greater clarity and more careful wording. Specific examples include:

"The evolution from low-grade to metastatic tumour is a major determinant of cancer mortality"
This statement is not broadly accurate. In prostate cancer and many other tumor types, low-grade tumors do not typically progress to metastatic disease.

"…deeply profile 34 metastatic lesions from 9 patients by rapid autopsy"
The phrasing is inaccurate. While samples were obtained through a rapid autopsy program, the profiling itself was conducted using downstream analytical methods.

"We found that intra-tumour heterogeneity is an indicator of key evolutionary processes…"
As written, this statement is vague and does not clearly convey a specific or actionable insight.

"Unexpectedly, microenvironments only played a limited role while clonal evolution primarily promoted transcriptional noise"
This conclusion appears overstated and should either be substantially revised or removed.

"…into units of intra-tumour heterogeneity…" The term "units" is not defined and remains unclear in this context.

“In metastatic castration-resistant prostate cancer (mCRPC), patients have acquired resistance to androgen deprivation therapy (ADT) and androgen receptor pathway inhibitors (ARPI), leading to limited survival”
This sentence should be rephrased for clarity and grammatical correctness.

“Our findings shed light on patterns in the development of non-genetic heterogeneity…”
The argument here is difficult to follow. It remains unclear which layer(s) of biological information—genomic, epigenetic, or microenvironmental—are proposed to drive the observed heterogeneity. A more precise and clearly articulated interpretation of the findings is needed.

4. Overall, the manuscript would benefit from more cautious interpretation of the data, clearer acknowledgment of limitations, and improved precision in language throughout.
A separate limitations section should be included to clearly outline the key shortcomings of the study, particularly the small sample size and the fact that the analyses are based on end-stage disease samples.

(Remarks on code availability)
I have not assessed the code.

Reviewer #4

(Remarks to the Author)
I was not an initial reviewer. I was asked to provide my opinion if the authors had satisfactorily responded to Reviewer 2's comments. After reading the revised manuscript and the comments from Reviewer 2, it is my opinion that the authors for the most part addressed Reviewer 2's comments. One thing to consider, Reviewer 2 (comment 7) noted that results from other metastatic studies indicate that the metastatic niche shapes tumor cell heterogeneity. The authors responded that those studies were from earlier stages of metastatic disease or animal models and that this was a study of end-stage disease. This is a reasonable response, and the authors added text to the discussion. I think it would be helpful to edit the abstract as well. The abstract indicates rapid autopsy, which is clearly end stage disease, however, it also states “To define mechanisms underpinning metastatic development”. I think that “development” gives the impression of earlier stage disease when their samples were therapy resistant end stage disease. Overall, this paper is intriguing and will be of interest to the field.

While evaluating the manuscript, I noted minor issues with a few figure panels that would benefit from editing.
1. It is difficult to evaluate the AR and INSM1 expression levels by the key in Figure 1a. It is difficult to see the IHC staining Figure 1c.
2. In Figure 2b, there are three unlabeled clusters, and it is not clear to which patient(s) they belong. The text indicates that CA34 had homogenous high AR expression, but Figure 2b does not reflect that.
3. In Figure 5c, the labels “Glycolysis” and “Cycling” are mixed up.
4. In Figure S2, the “0” expression for NE is all blue which makes the clusters appear uniformly high for NE markers when that is not the case.

(Remarks on code availability)

Open Access This Peer Review File is licensed under a Creative Commons Attribution 4.0 International License, which permits use, sharing, adaptation, distribution and reproduction in any medium or format, as long as you give appropriate credit to the original author(s) and the source, provide a link to the Creative Commons license, and indicate if changes were made.
In cases where reviewers are anonymous, credit should be given to 'Anonymous Referee' and the source.
The images or other third party material in this Peer Review File are included in the article's Creative Commons license, unless indicated otherwise in a credit line to the material. If material is not included in the article's Creative Commons license and your intended use is not permitted by statutory regulation or exceeds the permitted use, you will need to obtain permission directly from the copyright holder.
To view a copy of this license, visit https://creativecommons.org/licenses/by/4.0/

REVIEWERS' COMMENTS

The line number in the file refers to the lines of "Weng et al-Manuscirpt+Method-tracked.docx"

Reviewer #1 (Remarks to the Author):

In the revised version of the manuscript titled "Recurrent intra-tumour heterogeneity is a hallmark of metastatic prostate cancer," Weng et al. have addressed many of the reviewers' comments; however, several aspects of the manuscript still require revision, as they could be misleading to readers.

1. The assertion that copy number variations (CNVs) primarily contribute to transcriptional noise is overly simplistic and potentially misleading. In many cases, it is difficult to identify a single driver gene within a CNV region, as multiple genes may be simultaneously altered and contribute to the observed phenotype. While certain CNVs may have clear biological relevance linked to specific loci, many involve broader genomic regions with complex, multi-gene effects. The authors should therefore temper this claim and provide a more nuanced discussion of the interplay between genomic alterations and transcriptional states.

We thank reviewer for their perspective . We have toned down statements and added discussion about the complex interplay of multi-gene effects, CNVs across different regions and the involvement of regulatory regions affected by CNVs, as well as clarity and add caveats to our definition of "transcriptional noise".

We have added these caveats to lines 173 ("Clonal evolution leads to transcriptional noise but not de novo functional states"), 240-244 ("Overall, this suggest that transcriptional heterogeneity is linked to large-scale copy number clonal evolution, consistent with CNVs affecting multiple genes as well are regulatory regions. On a global level, largely contributes to inter- and intra-patient heterogeneity by introducing what could be considered "transcriptional noise", increasing expression variation to cells, without resulting in the emergence of novel cell functional states."), and 503-505 ("Our findings indicate that inter-lesion and inter-patient transcriptional heterogeneity is shaped by the expression changes linked to large-scale copy-number alterations. This could be considered 'transcriptional noise' as it does not contribute to the emergence of functional cell states.").

2. A similar concern applies to the interpretation of microenvironmental influences on cell states. The cohort analyzed here is small (9 patients), which substantially limits the generalizability of the conclusions. There is ample evidence in the literature that specific tumor cell states are enriched in particular metastatic niches (for example, neuroendocrine prostate cancer in liver metastases). The absence of such patterns in this limited dataset does not negate their biological relevance. The authors should exercise caution in making broad or generalized statements and more clearly acknowledge the limitations imposed by sample size.

We have added explicit acknowledgement of the small sample size to our discussion and stated that our results are best interpreted as an in-depth analysis rather than conclusions that are generalisable across patients (lines 550-552 ("Given the limited number of patients, we have focused our analysis on in-depth intra-patient tumour evolution across multiple lesions, and therefore our results are best interpreted in that context rather than in terms of broad inter-patient generalizability.")).

3. Throughout the manuscript, several statements lack precision or are potentially misleading. The overall writing would benefit from greater clarity and more careful wording. Specific examples include:

"The evolution from low-grade to metastatic tumour is a major determinant of cancer mortality"
This statement is not broadly accurate. In prostate cancer and many other tumor types, low-grade tumors do not typically progress to metastatic disease.

The sentence has now been edited to "The evolution to metastatic disease is a major determinant of cancer mortality".

"...deeply profile 34 metastatic lesions from 9 patients by rapid autopsy"
The phrasing is inaccurate. While samples were obtained through a rapid autopsy program, the profiling itself was conducted using downstream analytical methods.
We have modified to "deeply profile 34 metastatic lesions obtained from 9 patients through rapid autopsy".

"We found that intra-tumour heterogeneity is an indicator of key evolutionary processes..."
As written, this statement is vague and does not clearly convey a specific or actionable insight.
We have modified to "We find that there is evolutionary convergence of intra-tumour heterogeneity"

"Unexpectedly, microenvironments only played a limited role while clonal evolution primarily promoted transcriptional noise"
This conclusion appears overstated and should either be substantially revised or removed.
We have modified to "We find little evidence of the microenvironment driving transcriptional heterogeneity, but there are signatures of co-adaptation between the microenvironment and tumour cells. In contrast, clonal evolution primarily fostered widespread transcriptional changes that did not result in novel functional states.."

"...into units of intra-tumour heterogeneity..." The term "units" is not defined and remains unclear in this context.
We have modified to "Our findings reveal functional evolutionary convergence of metastatic disease into distinct intra-tumour subpopulations, identifying critical

determinants for therapeutic targeting."

"In metastatic castration-resistant prostate cancer (mCRPC), patients have acquired resistance to androgen deprivation therapy (ADT) and androgen receptor pathway inhibitors (ARPI), leading to limited survival"
This sentence should be rephrased for clarity and grammatical correctness.

We have modified to: "In patients with metastatic castration-resistant prostate cancer (mCRPC), cancers have progressed despite androgen deprivation therapy (ADT) and, in many cases, androgen receptor pathway inhibitors (ARPIs), leading to poor clinical outcomes."

"Our findings shed light on patterns in the development of non-genetic heterogeneity..."
The argument here is difficult to follow. It remains unclear which layer(s) of biological information—genomic, epigenetic, or microenvironmental—are proposed to drive the observed heterogeneity. A more precise and clearly articulated interpretation of the findings is needed.

The sentence was modified to "Our findings shed light on how transcriptional changes underlie this recurrent intra-tumour plasticity in end stage prostate cancer."

4. Overall, the manuscript would benefit from more cautious interpretation of the data, clearer acknowledgment of limitations, and improved precision in language throughout. A separate limitations section should be included to clearly outline the key shortcomings of the study, particularly the small sample size and the fact that the analyses are based on end-stage disease samples.
We have made the requested changes and added an extra paragraph to the Discussion section to clarify the sample size limitations and end-stage disease context (lines 679-681).

Reviewer #1 (Remarks on code availability):

I have not assessed the code.

The latest code is publicly available through the Github link in the "Data availability" section (deposited in https://doi.org/10.5281/zenodo.20019863).

<u>Reviewer #4 (Remarks to the Author):</u>

I was not an initial reviewer. I was asked to provide my opinion if the authors had satisfactorily responded to Reviewer 2's comments. After reading the revised manuscript and the comments from Reviewer 2, it is my opinion that the authors for the most part addressed Reviewer 2's comments. One thing to consider, Reviewer 2 (comment 7) noted that results from other metastatic studies indicate that the metastatic niche shapes tumor cell heterogeneity. The authors responded that those studies were from earlier stages of metastatic disease or animal models and that this

was a study of end-stage disease. This is a reasonable response, and the authors added text to the discussion. I think it would be helpful to edit the abstract as well. The abstract indicates rapid autopsy, which is clearly end stage disease, however, it also states “To define mechanisms underpinning metastatic development”. I think that “development” gives the impression of earlier stage disease when their samples were therapy resistant end stage disease. Overall, this paper is intriguing and will be of interest to the field.

We thank the reviewer for their support. We have updated the suggested sentence to “To define mechanisms underpinning metastatic heterogeneity in late-stage disease”

While evaluating the manuscript, I noted minor issues with a few figure panels that would benefit from editing.

1. It is difficult to evaluate the AR and INSM1 expression levels by the key in Figure 1a. It is difficult to see the IHC staining Figure 1c.

The resolution of AR and INSM1 intensity has been improved, and the background color was reduced to make the intensity easier to appreciate. Some expression might be hard to distinguish since its intensity is close to zero. This part of the results is explained in the figure legend. Figure 1c provides additional information, as samples later found to be NE, did not have strong INSM1 stain. Figure 1c shows microscopy images without any adjustment, that intentionally show negative expression of those markers.

2. In Figure 2b, there are three unlabeled clusters, and it is not clear to which patient(s) they belong. The text indicates that CA34 had homogenous high AR expression, but Figure 2b does not reflect that.

The labels positions have been changed to make the patient identities clearer. The expression is complementary to the one shown in Figure 2c. As the reviewer has pointed out, there was a typo in the patient ID. It has now been fixed to indicate this comment refers to patient CA35.

3. In Figure 5c, the labels “Glycolysis” and “Cycling” are mixed up.

We have fixed this.

4. In Figure S2, the “0” expression for NE is all blue which makes the clusters appear uniformly high for NE markers when that is not the case.

We have now unified the color scale of the expression plots to reduce the confusion. (Fig. S2-3)
